# Interacting Emission Species among Donor and Acceptor Moieties in a Donor-Grafted Polymer Host/TADF-Guest System and Their Effects on Photoluminescence and Electroluminescence

**DOI:** 10.1021/acsami.4c15933

**Published:** 2024-10-24

**Authors:** Yi-Hen Mao, Miao-Ken Hung, Shang-Ting Chung, Sunil Sharma, Kuen-Wei Tsai, Show-An Chen

**Affiliations:** Department of Chemical Engineering, National Tsing-Hua University, Hsinchu 30013, Taiwan, Republic of China

**Keywords:** organic light-emitting diode (OLED), polymer light-emitting diode (PLED), thermally activated delayed fluorescence (TADF), exciplex, excimer, aggregate, intramolecular charge transfer (ICT), donor−acceptor (D−A)

## Abstract

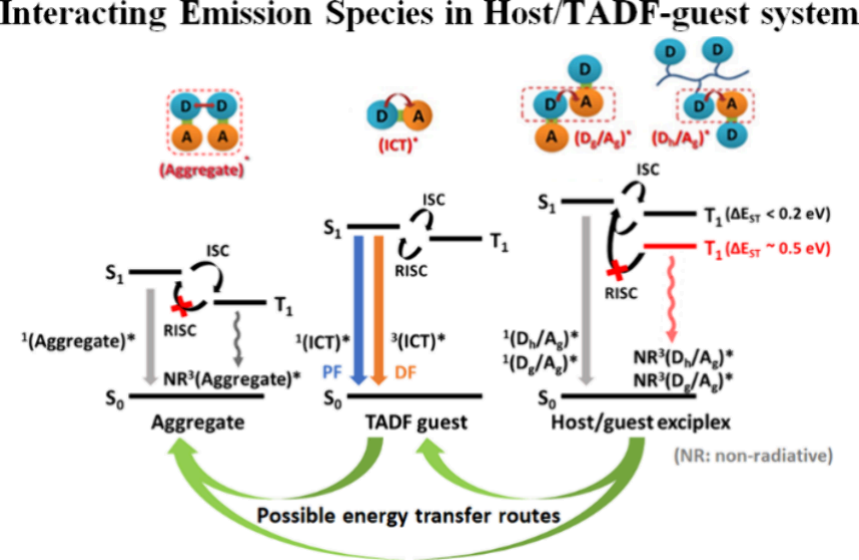

Thermally activated delayed fluorescence (TADF)-based electroluminescence (EL) devices adopting a host/guest strategy in their emitting layer (EML) are capable of realizing high efficiency. However, TADF emitters composed of donor and acceptor moieties as guests dispersed in organic host materials containing a donor and/or an acceptor are subject to donor–acceptor (D–A) interactions. In addition, electron delocalization between neighboring emitter molecules could form different species of aggregates. Here, we investigate the effects of intermolecular interacting emission species on the optoelectronic properties of sky-blue/green/red (sB/G/R) TADF emitters as guests using poly(biphenyl-Si/Ge) grafted with various donor moieties as hosts. We found the presence of guest/guest exciplex (D_g_/A_g_)*, host/guest exciplexes (D_h_/A_g_)*, and aggregates through the exploration of interactions between neighboring TADF guest molecules and between host and TADF-guest molecules. The nonradiative ^3^(D_h_/A_g_)* (Δ*E*_ST_ ≈ 0.5 eV) could increase the internal conversion rate (k_IC_) and reduce delayed luminescence, and both of them could cause a decrease in PLQY. The luminescence of ^3^(D_h_/A_g_)* may have a positive or negative effect on PLQY depending on its triplet energy. As the singlet and triplet energies of (aggregate)* are lower than those of (ICT)*, energy transfer from (ICT)* to (aggregate)* could occur. The low PLQY nature of (aggregate)* means that it is more likely to cause quenching in device emission. The emissions from (D_h_/A_g_)* and (aggregate)* are found to have increased full width at half-maximum and lead to lower emission color purity. Such intermolecular interactions should also occur in host/guest (TADF) systems and nondoped TADF emitter systems and thus are important factors for the molecular design of the TADF emitter and/or its accompanying host for high device efficiency and emission color purity.

## 1 Introduction

Third-generation light-emitting materials, known as metal-free thermally activated delayed fluorescence (TADF) emitters in organic light-emitting diodes (OLEDs), consist of donor and acceptor moieties interconnected by a spacer. This strategy represents an effective approach for realizing near 100% exciton harvesting by converting triplets to singlets via reversed intersystem crossing (RISC) when the difference in its singlet and triplet energy levels (Δ*E*_ST_) is small (below 0.2 eV).^1,2^ To achieve high-performance TADF OLEDs, the host/guest strategy for the emitting layer (EML) is commonly used, in which the TADF material as a guest is dispersed in the host matrix for suppressing unfavorable triplet–triplet annihilation (TTA) and triplet-polaron quenching,^3−5^ thus leading to a promotion of the device efficiency. Nowadays, the majority of TADF OLEDs with high efficiency (external quantum efficiency, EQE, over 30%) adopt the host/guest strategy in the EML.^6−12^ In the host/guest system, a typical molecular structure of the TADF emitter^3,13,14^ is composed of a donor–acceptor (D-A), donor–acceptor–donor (D–A–D), or multidonor–acceptor (mD–A) configuration, in which its D or A moiety molecule structure is similar to that of its host material. Therefore, interactions between donors and acceptors in host–guest and guest–guest molecules could occur. For example, various D and/or A moieties on the host can interact with the counter moiety in the TADF guest and produce different levels of (1) polarities that affect the emission color and Δ*E*_ST_ value of the TADF guest,^15−22^ (2) host–guest dipole–dipole interactions generated by the excited-state dipole moment between the host and guest that leads to charge-exchange-induced exciton quenching as the dipole of the host increases,^15,23^ and (3) exciton quenching of the TADF guest due to the shallower HOMO of the host than that of the TADF emitter.^24,25^ In addition, electron delocalization between two neighboring molecules with the same structure could form different species of aggregates. These emission species have lower energy levels than those of the monomeric emitter^26^ and give a significant red-shift emission relative to the TADF emitters.^27^

Donor-type small molecules as hosts have been successful in achieving the highest efficiencies for red, green, and blue TADF OLEDs using the vacuum deposition process along with the host/guest strategy in the EML, with maximum EQEs of 36.1, 39.1, and 38.8%, respectively.^28−30^ Additionally, our research group has also developed an effective σ–π conjugated polymer host, poly(acridan grafted biphenyl germanium) P(DMAC-Ge), which has the highest triplet energy (E_T_) of 2.86 eV among conjugated polymers for highly efficient full-color electroluminescence (EL) devices via the solution process.^22^ This polymer host/guest device has achieved a record-high EQE of 24.1% in sky-blue TADF PLEDs and a state-of-the-art EQE of 22.5% in T-P (TADF and phosphorescence emitters) hybrid white PLEDs.^22,31^ As mentioned before, these results highlight the importance of the host/guest strategy in the fabrication of OLED devices.

Furthermore, the presence of a host/TADF-guest exciplex should be the other crucial issue to affect the luminescence properties of the TADF emitter. Generally, it is easy to form exciplexes for the molecules with D and A moieties in bicomponent and multicomponent solid films; similarly, exciplexes should also emerge in the host/TADF-guest system containing D and/or A moieties. However, studies on exciplex formation by host/TADF-guest interaction and guest/guest intermolecular interaction are rare.^32^ It was found that exciplex emission from host/TADF-guest interaction and from aggregate emission in the solid-state film could occur, but such effects on EL by interaction emission species have not been reported. Therefore, it is highly desirable to explore the underlying mechanisms of host–guest and guest–guest intermolecular interactions in solid films for further improving the performance of TADF OLEDs. When host/TADF-guest exciplex and aggregate formations occur, competitive processes between TADF emission and exciplex/aggregate formation would affect the PL quantum yield (PLQY) of the TADF emitter and consequently the EQE of devices.

**Scheme 1:**
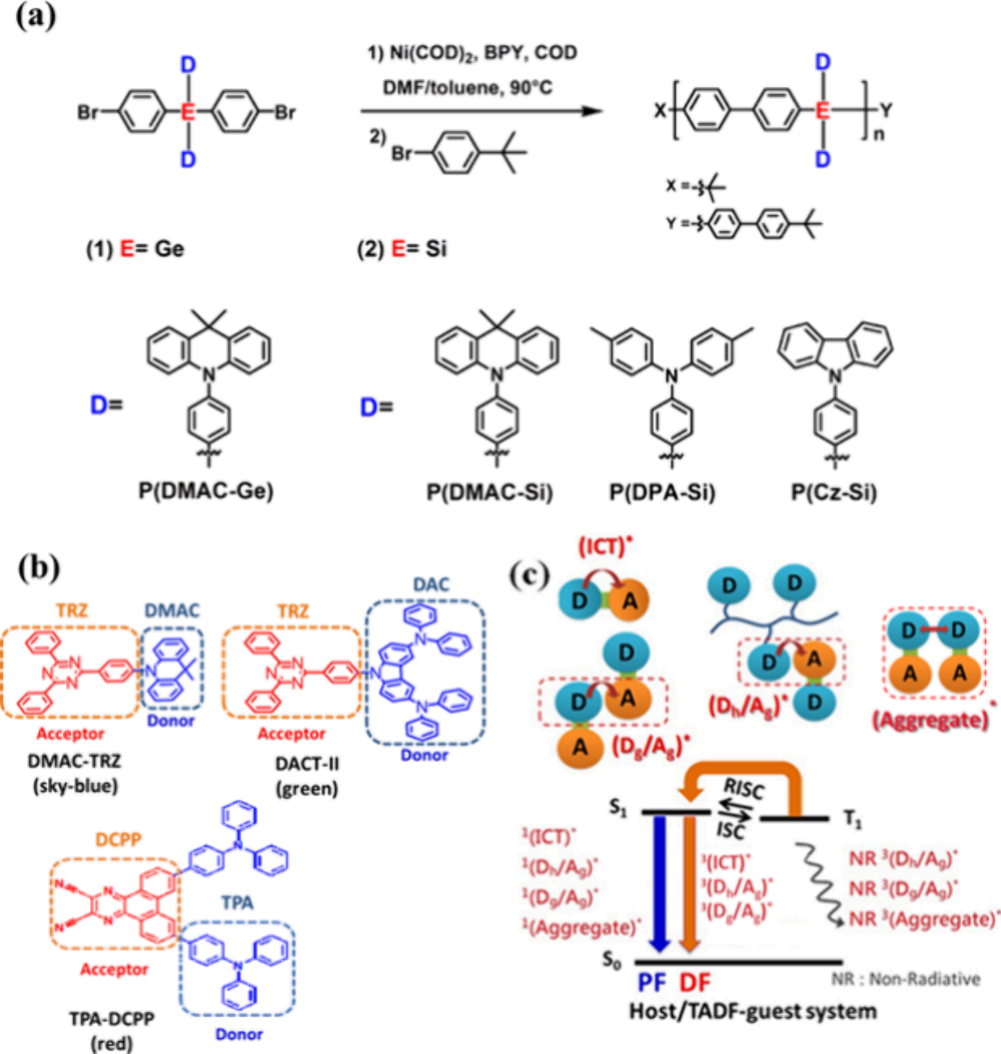
Chemical Structures of (a) D-Polymer Hosts: P(DMAC-Ge), P(DMAC-Si), P(DPA-Si), and P(Cz-Si), (b) the sB/G/R TADF Emitters Adopted Here, and (c) Possible Emitting Species in the Host/TADF-Guest System and Their Jablonski Diagram The donor moieties and the acceptor moieties of emitters are 9,9-dimethyl-9,10-dihydroacridine (DMAC), diphenylaminocarbazole (DAC), triphenylamine (TPA), 2,4,6-triphenyl-1,3,5triazine (TRZ), and 2,3-dicyanopyrazino phenanthrene (DCPP).

Here, we propose a series of σ–π conjugated polymers as hosts with Si- and Ge-biphenyl as backbone repeat units and amine-type donor (DPA), acridan-type donor (DMAC), and carbazole-type donor (Cz) moieties as side arms linked to Si and Ge with phenyl as spacers (Scheme 1a,b), to explore the structural effects of donor moieties on the optoelectronic characteristics of specific sky-blue/green/red (sB/G/R) TADF emitters, resulting from the formations of D/A interacting species (Scheme 1c). The singlets and triplets of aggregates and host/guest exciplexes from D/A interactions between the donor-grafted polymer (D-polymer) host and TADF emitters and between two neighboring guest molecules are identified. Their influences on PLQYs, internal conversion rates (k_IC_), and reverse internal-system crossing (RISC) rates (k_RISC_) of specific sB/G/R TADF emitters are thoroughly investigated. The devices using these D-polymers as hosts and the sB/G/R TADF emitters as guests show a high correlation between their EQEs and PLQYs. We also explore how structure tuning of the host for a specific TADF emitter can lead to high device performance. The D/A interactions explored here should also be applicable to a small-molecule host/guest system as well as to a nondoped TADF emitter system.

## 2 Materials and Methods

### 2.1 Materials

**Scheme 2:**
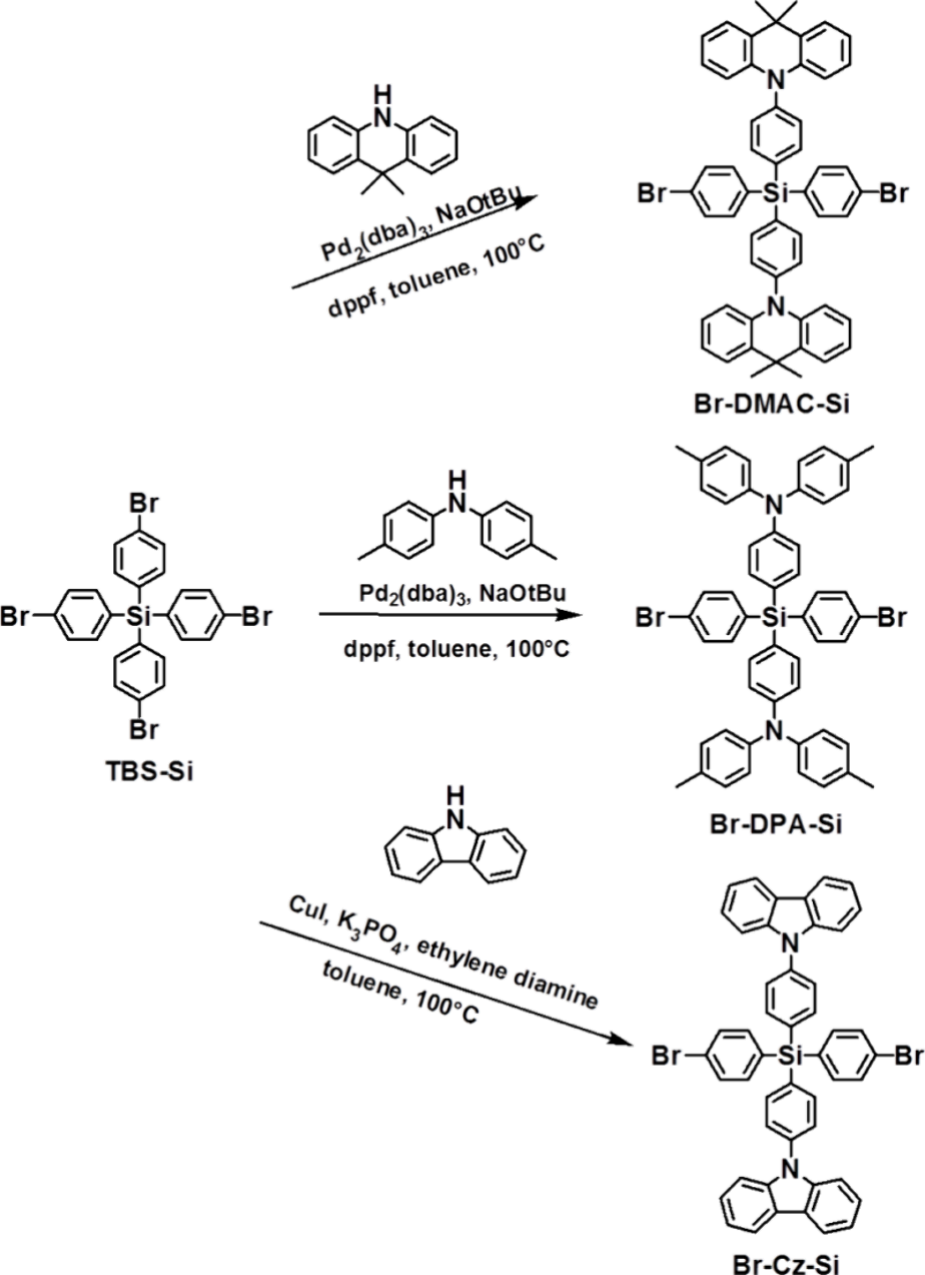
Synthetic Route for Monomers

**Scheme 3:**
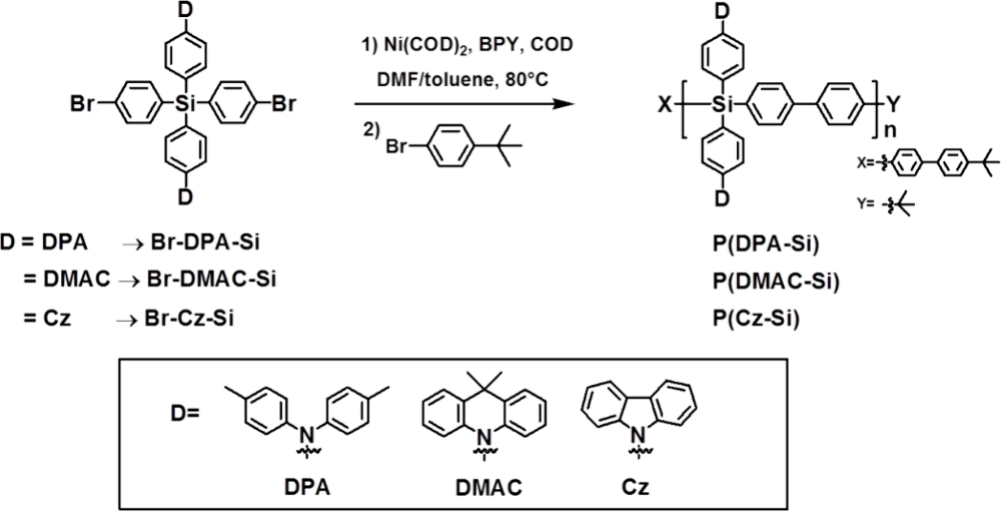
Synthetic Route for D-Polymer Hosts

9H-Carbazole, di-*p*-tolylamine, silicon tetrachloride, (SiCl_4_), BPY, ethylene diamine, copper(I) iodide (CuI), and 1,4-dibromobenzene were purchased from Alfa-Aesar (99.0% purity); *n*-butyllithium was purchased from Chemetall Taiwan Co. Ltd.; sodium tert-butoxide, tris(dibenzylideneacetone)dipalladium(0) (Pd_2_(dba)_3_), and COD were bought from Acoss Organics; 9,9-dimethyl-acridan was from Shine Materials Technology Co. Ltd.; and bis(1,5-cyclooctadiene) nickel (Ni(COD)_2_) was obtained from STREM Chemicals Inc. Anhydrous toluene and dimethylformamide (DMF) were purchased from Aldrich and stored over molecular sieves before using. Tetrahydrofuran (THF) and diethyl ether were purchased from commercial suppliers and dried by refluxing over sodium metal with the indicator benzophenone. All reactions were monitored by thin-layer chromatography, and compounds were purified by column chromatography on silica gel using hexane and ethyl acetate or dichloromethane. The chemical structures and synthetic routes of the monomers Br-DMAC-Si, Br-DPA-Si, and Br-Cz-Si and the polymers P(DPA-Si), P(DMAC-Si), and P(Cz-Si) are shown in Schemes 2 and 3, respectively. The weight-average molecular weight (*M*_w_) and polydispersity values are 66,000 and 4.96 Da for P(DPA-Si), 36,000 and 3.03 Da for P(DMAC -Si), and 58,000 and 5.04 Da for P(Cz -Si), as measured by using GPC analysis with narrow *M*_w_ polystyrene as calibration standards. All of these polymers show good solubility in common organic solvents, such as chloroform, chlorobenzene, ortho-dichlorobenzene, and THF.

### 2.2 Synthetic Methods

#### 2.2.1 Synthesis of **TBS-Si**

It was prepared according to a procedure reported in the literature ref S1.

#### 2.2.2 Synthesis of **Br-DMAC-Si**

The chemical structure and synthetic route of DMAC-Si are shown in Scheme 2. The mixture of **TBS-Si** (3.3 g, 5 mmol) and 9,9-dimethyl-acridan (1.3 g, 6 mmol) in toluene (30 mL) was purged with nitrogen for 30 min. Then, sodium *tert*-butoxide (0.72 g, 7.5 mmol), Pd_2_(dba)_3_ (46 mg, 0.05 mmol), and 1,1′-bis(diphenylphosphino)ferrocene (55 mg, 0.1 mmol) were added to the mixture and purged with nitrogen for an additional 10 min. The reaction mixture was heated at 110 °C for 12 h under a nitrogen atmosphere. The brown suspension was then allowed to cool to room temperature, after which water was added and the mixture was extracted with CHCl_3_. The organic layer was washed with water and dried over MgSO_4_, and the solvent was removed under vacuum. The residue was purified by column chromatography on silica gel, eluting with hexane/CH_2_Cl_2_ = 4:1, to yield the product **Br-DMAC-Si** (0.57 g, 13%) as a white solid. ^1^HNMR (500 MHz, CDCl_3_). δ (ppm): 7.82 (d, *J* = 8.5 Hz, 4H), 7.62 (d, *J* = 8.0 Hz, 4H), 7.52 (d, *J* = 8.0 Hz, 4H), 7.45 (dd, *J* = 8.0 Hz, 1.0 Hz, 4H), 7.41 (d, *J* = 8.0 Hz, 4H), 6.98 (td, *J* = 1.5 Hz, 4H), 6.92 (td, *J* = 1.0 Hz, 4H), 6.28 (dd, *J* = 8.0 Hz, 1.0 Hz, 4H), 1.67 (s, 12H). ^13^C NMR (125 MHz, CDCl_3_): δ = 143.2, 140.7, 138.7, 137.8, 133.0, 132.0, 131.6, 131.1, 130.3, 126.3, 125.5,125.2, 120.8, 114.0, 36.0, 31.0. MS (FAB+) calcd for C_54_H_44_Br_2_SiN_2_ ([M]^+^): *m*/*z* 909.2. Found: *m*/*z* 909. The ^1^HNMR spectra and ^13^CNMR are given in the Supporting Information (S4).

#### 2.2.3 Synthesis of **Br-DPA-Si**

The chemical structure and synthetic route of DMAC-Si are shown in Scheme 2. It was prepared with the same procedure as for **Br-DMAC-Si** but di-*p*-tolylamine was used as the starting material. The residue was purified by column chromatography on silica gel, eluting with hexane/CH_2_Cl_2_ = 9:1, to yield the product **Br-DPA-Si** (0.71 g, 16%) as a white solid. ^1^HNMR (500 MHz, CDCl_3_), δ (ppm): 7.47 (d, *J* = 8.0 Hz, 4H), 7.38 (d, *J* = 8.0 Hz, 4H), 7.25 (t, 4H), 7.01 (q, 16H), 6.93 (d, *J* = 8.5 Hz, 4H), 2.29 (s, 12H). ^13^C NMR (125 MHz, CDCl_3_): δ = 149.7, 144.7, 137.8, 136.9, 133.8, 133.3, 131.0, 130.0, 125.4, 124.6, 123.7, 120.2, 20.8. MS (FAB+) calcd for C_52_H_44_Br_2_SiN_2_ ([M]^+^): *m*/*z* 884.2. Found: *m*/*z* 884. The ^1^HNMR spectra and ^13^CNMR are given in the Supporting Information (S4).

#### 2.2.4 Synthesis of **Br-Cz-Si**

The chemical structure and synthesis routes of DMAC-Si are shown in Scheme 2. The mixture of tetrakis(4-bromophenyl)silane (5.0 g, 7.67 mmol) and 9H-carbazole (2.56 g, 15.34 mmol) in toluene (75 mL) was purged with nitrogen for 20 min. Then, tribasic potassium phosphate (4.88 g, 23 mmol), copper(I) iodide (730 mg, 3.8 mmol), and ethylene diamine (256 μL, 3.8 mmol) were added to the mixture and purged with nitrogen for an additional 10 min. The reaction mixture was heated at 100 °C overnight under a nitrogen atmosphere. The brown suspension was then allowed to cool to room temperature, after which water (50 mL) was added and the mixture was extracted with DCM. The organic layer was washed with water and dried over MgSO_4_, and the solvent was removed under a vacuum. The residue was purified by column chromatography on silica gel, eluting with hexane/DCM = 4:1, to yield the product (2.70 g, 42%) as a white solid. ^1^HNMR (500 MHz, CDCl_3_). δ (ppm): 8.14 (d, *J* = 8.0 Hz, 4H), 7.82 (d, *J* = 8.0 Hz, 4H), 7.67 (d, *J* = 8.0 Hz, 8H), 7.63 (d, *J* = 8.5 Hz, 8H), 7.54 (d, *J* = 8.0 Hz, 4H), 7.51 (d, *J* = 8.0 Hz, 4H), 7.41 (t, 4H), 7.29 (t, 4H). ^13^C NMR (125 MHz, CDCl_3_): δ = 140.5, 139.7, 137.8, 137.7, 132.0, 131.9, 131.6, 126.5, 126.0, 125.5, 123.6, 120.4, 120.3, 109.8. MS (FAB+) calcd for C_48_H_32_Br_2_SiN_2_ ([M]^+^): *m*/*z* 824.1. Found: *m*/*z* 824. The ^1^HNMR spectra and ^13^CNMR are given in the Supporting Information (S4).

#### 2.2.5 General Polymerization Procedure for Three D-Polymers: P(DMAC-Si), P(DPA-Si), and P(Cz-Si)

The monomer (0.3 mmol), bis(1,5-cyclooctadiene) nickel (0) (Ni(COD)_2_) (182 mg, 0.66 mmol), 2,2-bipyridyl (BPY) (103 mg, 0.66 mmol), 1,5-cyclooctadiene (COD) (71 mg, 0.66 mmol), anhydrous DMF (1 mL), and anhydrous toluene (3 mL) were added into a reactor under a nitrogen atmosphere. The polymerization proceeded at 80 °C for 4 days, and 1-bromo-4-tert-butylbenzene as an end-capping agent (0.040 mL, 0.24 mmol) was added to the reaction mixture and then continually reacted for an additional 24 h. The resulting polymer was poured into methanol and stirred for 30 min. The precipitate was collected by filtration and dried and then dissolved in CHCl_3_. Chloroform was washed with water, dried over anhydrous MgSO_4_, and evaporated under reduced pressure. The material was redissolved in CHCl_3_ and again precipitated in methanol. The precipitate was collected by filtration and dried under a high vacuum for 24 h.

#### 2.2.6 P(DMAC-Si)

The chemical structure and synthetic route of DMAC-Si are shown in Scheme 3. The pale-yellow solid product so obtained was subject to GPC analysis, giving a weight-average molecular weight (*M*_w_) and polydispersity of 36,000 Da and 3.03, respectively, relative to polystyrene standards. ^1^HNMR (500 MHz, CDCl_3_). δ (ppm): 7.93–8.00 (4H), 7.79–7.85 (8H), 7.39–7.43 (8H), 6.88–6.98 (8H), 6.30–6.33 (4H), 1.62–1.69 (12H). The ^1^HNMR spectra are given in the Supporting Information (S4).

#### 2.2.7 P(DPA-Si)

The chemical structure and synthetic route of DMAC-Si are shown in Scheme 3. The white solid product so obtained was subject to GPC analysis, giving a weight-average molecular weight (*M*_w_) and polydispersity of 66,000 Da and 4.96, respectively, relative to polystyrene standards. ^1^HNMR (500 MHz, CDCl_3_). δ (ppm): 7.41–7.50 (4H), 7.35–7.40 (4H), 7.21–7.32 (4H), 6.96–7.12 (16H), 6.90–6.99 (4H), 2.23–2.31 (12H). The ^1^HNMR spectra are given in the Supporting Information (S4).

#### 2.2.8 P(Cz-Si)

The chemical structure and synthesis route of DMAC-Si are shown in Scheme 3. The white solid product so obtained was subject to GPC analysis, giving a weight-average molecular weight (*M*_w_) and polydispersity of 58,000 Da and 5.04, respectively, relative to polystyrene standards. ^1^HNMR (500 MHz, CDCl_3_). δ (ppm): 8.06–8.15 (4H), 7.76–7.91 (12H), 7.59–7.68 (4H), 7.43–7.57 (4H), 7.29–7.39 (5H), 7.10–7.22 (3H). The ^1^HNMR spectra are given in the Supporting Information (S4).

### 2.3 Thermal, Optical, and Electrochemical Properties

The thermal properties of these D-polymers were measured by differential scanning calorimetry (DSC) (Figure S1 and Table S1), giving similar high glass-transition temperatures (*T*_g_) lying between 255 and 261 °C; their thermal stabilities were measured by thermogravimetric analysis (TGA) (Figure S2 and Table S1). The thermal decomposition temperatures (*T*_d_) at 5% weight-loss for P(DMAC-Ge), P(DMAC-Si), P(DPA-Si), and P(Cz-si) are 434.4, 432.7, 406.2, and 498 °C, respectively. The TGA measurement results indicate that these D-polymers have good thermal stability. The UV–vis absorption (UV), photoluminescence (PL), and phosphorescence (Ph) spectra of the D-polymer hosts as solid films are shown in Figure S3. The broad absorption band around 300 nm for P(DPA-Si) is composed of the n−π* transition of the TPA moiety (305 nm) and the π–π* transition of the Si-biphenyl backbone (271 nm) refs S2 and S3. The absorption peaks around 280 nm and shoulders at 290 nm for P(DMAC-Si) and P(DMAC-Ge) are contributed by the π–π* transitions of Si- and Ge-biphenyl backbones and the π–π* transition of the DMAC moiety, respectively. For P(Cz-Si), the absorption peaks around 295 nm and the absorption band from 325 to 350 nm correspond to the π–π* transition of the Cz moiety, and the broad peak around 250 nm is assigned to the n−π* transition of the benzene group linking to the Cz moiety ref S4.

The PL peaks are 395, 405, 396, and 402 nm for P(DMAC-Ge), P(DMAC-Si), P(DPA-Si), and P(Cz-Si), respectively. The phosphorescence spectra of the D-polymer hosts as solid films were measured with a 1 ms delay time at 77 K, and their onsets at short wavelength sides were taken as their T_1_ energy levels. As shown in Table S1, the Ge-based D-polymer host P(DMAC-Ge) shows a much higher triplet energy (T_1_) level (2.86 eV) than that of the Si-based D-polymer hosts, P(DMAC-Si), P(DPA-Si), and P(Cz-Si), which are 2.78, 2.78, and 2.76 eV, respectively. All of the Si-based σ–π conjugated polymers show similar T_1_ levels due to the existence of multiple-triplet states ref S5, where their T_1_ levels are determined from the Si-based polymer main chain, which have lower T_1_ than that of the side arm group. Similarly, the high T_1_ of P(DMAC-Ge) arises from its higher T_1_ of the Ge-based main chain (2.85 eV) than that of the Si-based one (2.78 eV) ref S6. Cyclic voltammetry was performed to determine the oxidative curves of the D-polymer hosts (Figure S4), and the results are summarized in Table S1. Based on the oxidative curves, the HOMO levels are estimated to be −5.39, −5.40, −5.45, and −5.72 eV for P(DMAC-Ge), P(DMAC-Si), P(DPA-Si), and P(Cz-Si), respectively (Section S1C and Table S1). Their optical band gaps (E_g_) were determined from the onsets of the absorption spectra in CHCl_3_ as 3.31, 3.31, 3.32, and 3.52 eV, and the LUMO levels were thus calculated by subtraction of the E_g_ from the HOMO to give −2.08, −2.09, −2.13, and −2.20 eV for P(DMAC-Ge), P(DMAC-Si), P(DPA-Si), and P(Cz-Si), respectively.

### 2.4 Device Fabrication Method

The substrate of indium tin oxide (ITO) glass was treated with oxygen plasma for 5 min under a pressure of 200 mTorr and a power of 50 W. The hole injection materials poly(3,4-ethylenedioxythiophene)-poly(styrenesulfonate) (PEDOT:PSS), CLEVIOS P VP AI 4083, and CLEVIOS P VP CH 8000 were mixed in a 2:1 volume ratio and then spin-coated on the treated ITO substrate with a thickness of 30 nm. All of the D-polymer hosts and sB/G/R TADF emitters were dissolved in chlorobenzene (10 mg/mL), and each host was doped with 8 wt % emitter to obtain EML solutions, followed by spin-coating on top of the hole injection layer (PEDOT:PSS) with a thickness of 30 nm. The EML layer was annealed at 120 °C for 10 min. The layer of 1,3,5-tri(diphenylphosphoryl-phen-3-yl) benzene (TP3PO) (3 nm) was used as a high triplet exciton blocker, followed by coating the layer of 1,3,5-tri(m-pyridin-3-ylphenyl)benzene (TmPyPB) (52 nm) as a hole blocking and electron transport layer. Both layers were deposited by thermal evaporation in a vacuum of 2 × 10^–6^ Torr. Finally, a thin layer of CsF (approximately 1 nm) was deposited on top of the electron transport layer and then covered with an aluminum film (100 nm). Both layers were deposited by thermal evaporation in a vacuum of 2 × 10^–6^ Torr through a shadow mask.

## 3 Results and Discussion

### 3.1 Possible Interacting Emitting Species in the Host/TADF-Guest System

To investigate interactions in the host/TADF-guest systems, we propose a series of D-polymer hosts of P(DMAC-Ge), P(DMAC-Si), P(DPA-Si), and P(Cz-Si) as shown in Scheme 1a, among which P(DMAC-Ge) was first proposed by us very recently^22^ and the others are novel σ–π conjugated polymers with various donor-type side arms. All of them are synthesized by the Yamamoto polymerization method.^33^ Moreover, the well-known TADF emitters, DMAC-TRZ,^34^ DACT-II,^35^ and TPA-DCPP,^36^ are used as the sky-blue/green/red (sB/G/R) TADF guests, respectively (Scheme 1b). The emission behavior in the host/TADF-guest system could be more complicated than that in the nondoped TADF emitter system as the former involves additional D–A interaction between the host and guest and between two guest molecules. In addition to the intramolecular charge transfer (ICT)* emission from the TADF guest, the guest/guest exciplex (D_g_/A_g_)* emissions from the interaction between the donor moiety of the guest molecule and the acceptor moiety on the neighboring guest molecule, the aggregate emission from two π–π interacting emitter molecules (aggregates)*, and the host/guest exciplex (D_h_/A_g_)* emission from the interaction between the acceptor moiety on the TADF guest and the donor moiety on the neighboring D-polymer are all possible (Scheme 1c). These emitting species could form prompt fluorescence (PF) and delayed fluorescence (DF) based on the sources of exciton from the singlet state (S_1_) and up-converted triplet state (T_1_) to S_1_. Here, we denote them as radiative singlet emissions, ^1^(ICT)*, ^1^(D_g_/A_g_)*, ^1^(aggregate)*, and ^1^(D_h_/A_g_)* for the former and radiative up-converted triplet emissions, ^3^(ICT)*, ^3^(D_g_/A_g_)*, ^3^(aggregate)*, and ^3^(D_h_/A_g_)* for the latter. If the excitons of ^3^(D_g_/A_g_)*, ^3^(D_h_/A_g_), and ^* 3^(aggregate)* cannot be up-converted from T_1_ to S_1_ through RISC, they lead to nonradiative (NR) decay and are denoted as NR ^3^(D_g_/A_g_)*, NR ^3^(D_h_/A_g_)*, and NR ^3^(aggregate)*, respectively. All of the possible emitting species mentioned above are summarized in Scheme 1c.

**Figure 1:**
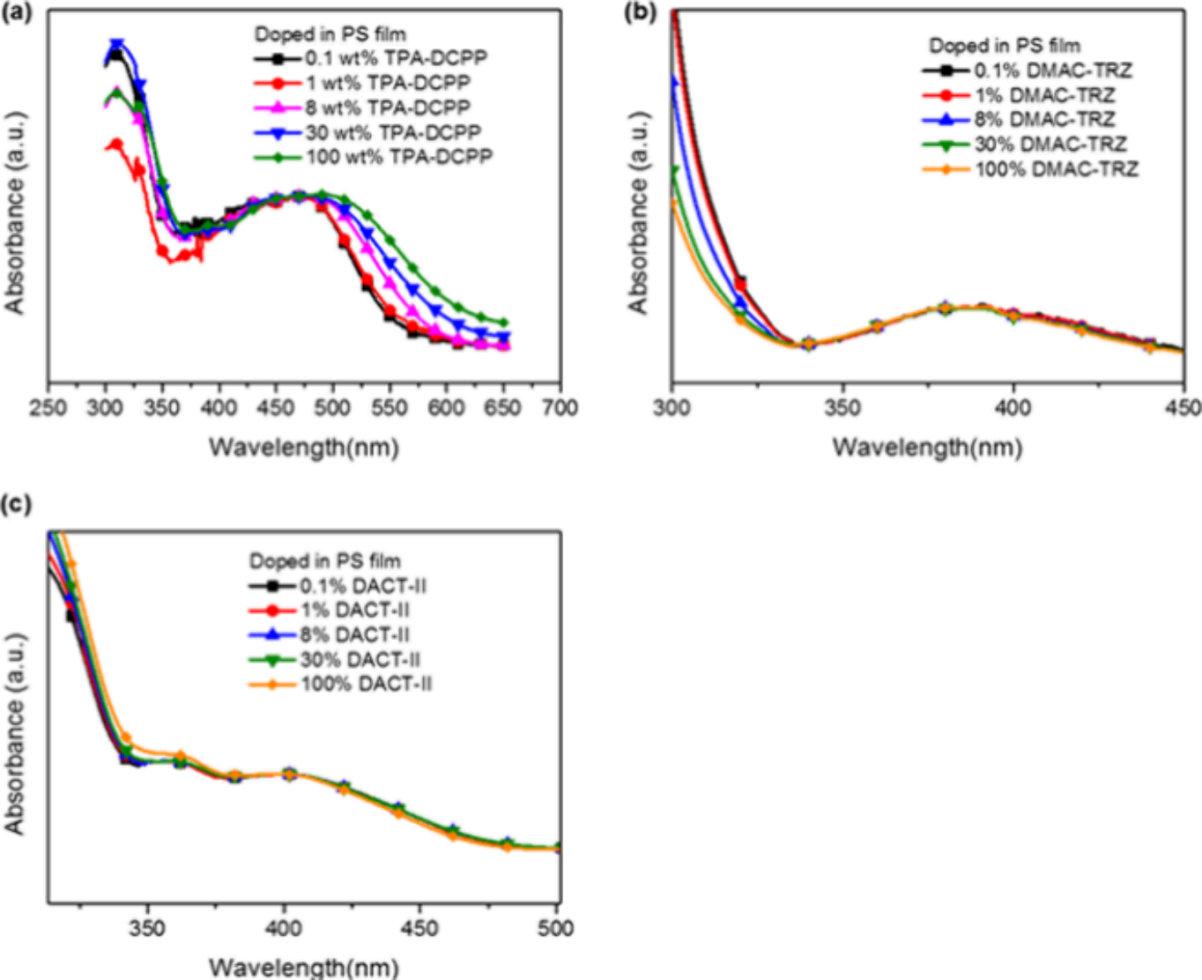
UV spectra of the doped polystyrene films for (a) TPA-DCPP normalized at 470 nm, (b) DMAC-TRZ normalized at 384 nm, and (c) DACT-II normalized at 397 nm at various doping concentrations.

To identify the formation of (D_g_/A_g_)* and (aggregate)*, we investigate the system of the R/G/sB emitter doped into the optically inert polymer, polystyrene (PS), in which (D_h_/A_g_)* should not appear since PS contains no donor or acceptor moiety to facilitate host–guest interaction. The PLQYs of 8 wt % DMAC-TRZ, DACT-II, and TPA-DCPP doped PS films are found as 99%, 99%, and 31%, while the PLQYs of the three emitter neat films are found as 83%, 43%, and 14%. The PLQYs of DACT-II and TPA-DCPP show drastic decreases by 56% and 17% as the concentration increases, indicating (D_g_/A_g_)* and (aggregate)* could appear in high concentrations. The UV–vis spectra of TPA-DCPP, DMAC-TRZ, and DACT-II doped PS films at 0.1–100 wt % are shown in Figure 1. The strong absorption at 300 nm of the TPA-DCPP UV spectra can be attributed to the π–π* transition (Figure 1a), while the weaker absorption bands at 350–650 nm can be attributed to the intramolecular charge transfer (ICT) transition from the TPA moiety to the DCPP moiety. Furthermore, the lower energy shoulders at 475–650 nm, which grow monotonically with dopant concentration from 0.1 to 100 wt %, potentially originate from the direct absorption of excitation energy by aggregates. On the contrary, the UV spectra of the DMAC-TRZ doped and DACT-II doped PS films show no absorption of aggregates at 384–450 nm for DMAC-TRZ and 400–500 nm for DACT-II as the dopant concentration increases from 0.1 to 100 wt % (Figure 1b,c), assuring the absence of aggregates in these two systems. The PL spectra of TPA-DCPP doped PS films show increased FWHM from 91 to 145 nm and a significant red shift as the dopant concentration increases from 0.1 to 100 wt % (Figure S5a), which could be attributed to the presence of (D_g_/A_g_)* and aggregates since (ICT)* emission gives FWHM normally within 70–100 nm.^37^ However, the PL spectra of DMAC-TRZ and DACT-II doped PS films with dopant concentrations increasing from 0.2 to 100 wt % show similar full width at half-maximum values of 77–86 and 83–94 nm, respectively (Figure S5b,c). To confirm the presence of (aggregate)* and its concentration dependence on TPA-DCPP doped PS films, the UV spectrum of the diluted film (0.1 wt %) is considered as the absorption by isolated TPA-DCPP molecules. Spectral subtractions for all UV–vis absorption spectra at higher concentrations from that of the 0.1 wt % film are performed, and the resulting spectra with normalization at the intersection point 470 nm are shown in Figure S6. In this way, we can separate the contributions from the absorptions of the (ICT)* luminophor and aggregates. The UV spectrum of aggregates in a 1 wt % TPA-DCPP doped PS film shows a peak at 529 nm, which becomes broader and slightly red-shifts as the dopant concentration increases. The broader and red-shifted spectrum implies the formation of different species of aggregates with various extents of aggregation.

**Figure 2:**
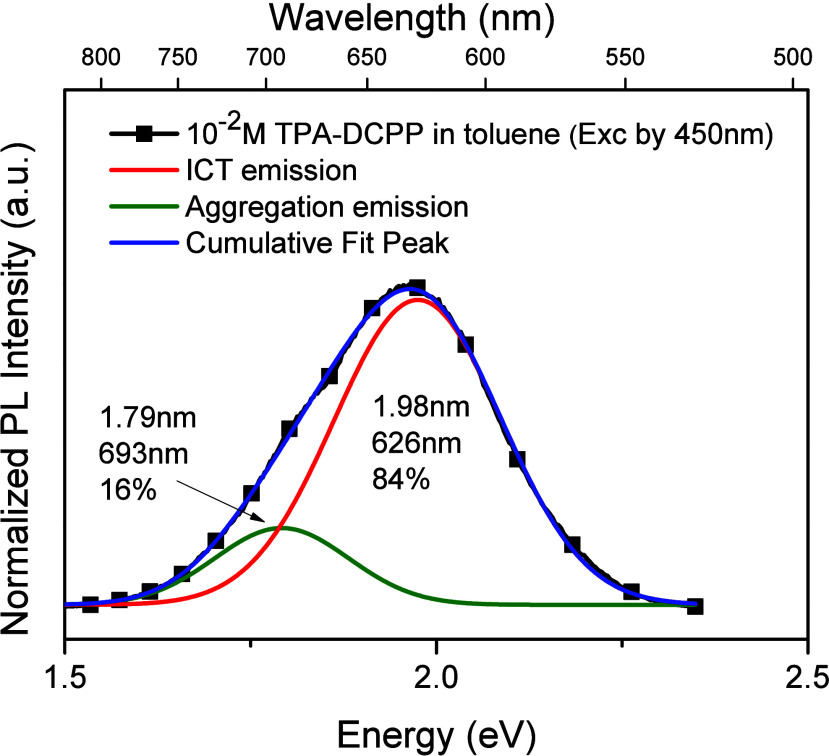
Deconvoluted plots and fractions of emitting species from the PL spectrum of the 10^–2^ M (0.88 wt %) TPA-DCPP solution in toluene (excited by 450 nm).

To further confirm the formation of aggregates in the TPA-DCPP doped PS system, the dependence of the PL spectrum of TPA-DCPP in toluene (10^–2^ M, 0.88 wt %) on excitation wavelength is shown in Figure S7a for the two excitation wavelengths at 450 and 560 nm (corresponding to the absorption spectra in Figure 1a). The PL spectrum for the (aggregate)* emission with a peak at 693 nm and an FWHM of 90 nm appears after excitation at the wavelength corresponding to the absorption of aggregates (560 nm). Changing the excitation wavelength to 450 nm produces a PL spectrum with a peak at 634 nm and an FWHM of 102 nm, which is 12 nm wider than the former spectrum, supporting the presence of multiple emission species in the PL spectrum of the TPA-DCPP solution excited at 450 nm. To identify the presence of (D_g_/A_g_)*, (ICT)*, and (aggregate)* emission species in the 10^–2^ M TPA-DCPP toluene solution, spectral deconvolutions were carried out (see Figure 2) by adopting the Gaussian function energy fit (which is used in all Gaussian fits in this work) because it reasonably fits the PL spectrum of TPA-DCPP in the diluted solution (Figure S8), in which no interaction between guest molecules could occur. As shown in Figure 2, the PL spectrum of the 10^–2^ M TPA-DCPP solution can be well-deconvoluted into two emission spectra, one peak at 628 nm and the other peak at 693 nm. The former peak with the higher fraction (84%) can be attributed to (ICT)* emission and the latter peak with the lower fraction (16%) can be attributed to (aggregate)*, as its wavelength is the same as that of (aggregate)* in Figure S7a.

The above assignment to (aggregate)* and (ICT)* emissions can be further supported by photoluminescence excitation (PLE) spectra monitored at monomeric (600 nm) and (aggregate)* (730 nm) emissions (Figure S7b). The PLE spectrum monitored at 600 nm shows similar absorption bands at 350–550 nm as the UV spectrum (Figure 1a), while that monitored at 730 nm shows a peak at 570 nm, which is close to that of the UV spectrum of (aggregate)* at 1 wt % in PS (Figure S6), indicating the existence of (aggregate)*. Furthermore, the absorption spectrum of (aggregate)* (covering 450–650 nm) is partially overlapped with the emission spectrum of (ICT)* (covering 550–700 nm), indicating a possible occurrence of energy transfer from (ICT)* to (aggregate)* by fluorescence resonance energy transfer, and therefore the actual fraction of (aggregate)* should be far less than 16% as indicated in Figure 2.

**Figure 3:**
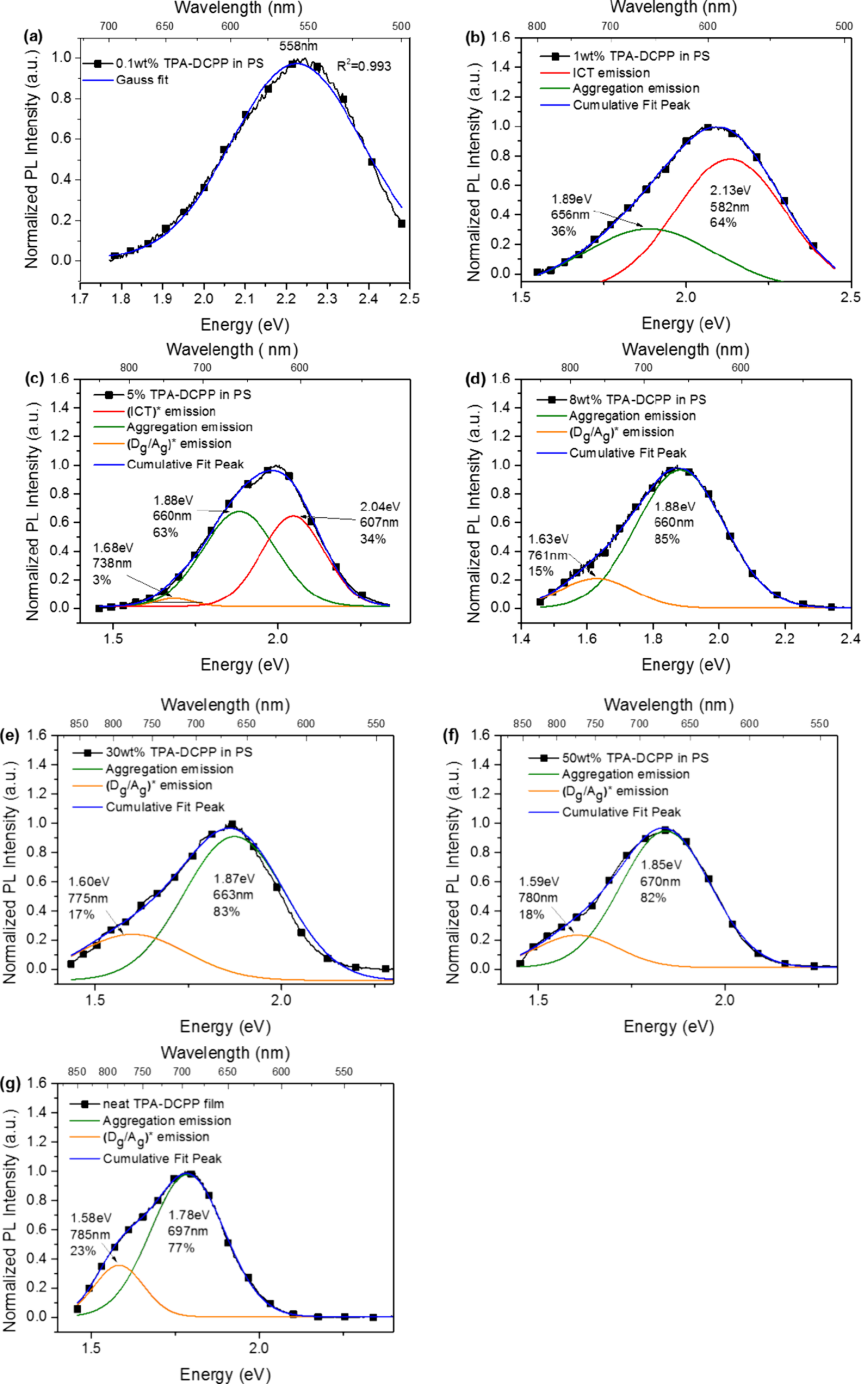
Deconvoluted plots and fractions of emitting species from the PL spectra of (a) 0.1 wt %, (b) 1 wt %, (c) 5 wt %, (d) 8 wt %, (e) 30 wt %, and (f) 50 wt % TPA-DCPP doped polystyrene films and (g) neat TPA-DCPP film.

To investigate guest–guest interactions between the donor moiety and the acceptor moiety of each type of sB, G, and R TADF emitters, PLs of DMAC and TRZ blend for DMAC-TRZ, DAC and TRZ blend for DACT-II, and TPA and DCPP blend for TPA-DCPP (all at 50:50 wt %) were carried out. As shown in Figure S9a–c, a new emission peak is observed for each blend film at 471, 542, and 758 nm. These peaks exhibit significant red-shifts compared to the emission spectra of the corresponding pure components DMAC/TRZ, DAC/TRZ, and TPA/DCPP. This observation indicates a possible formation of new emission species in each blend film, that is, an exciplex formation. The TPA/DCPP exciplex emission has a much more red-shifted emission than ICT, which can be explained by the TPA molecule having a stronger electron-donating ability and a shallower HOMO level than the DPA molecule, which leads to forming exciplexes with a much more red-shifted emission spectrum. For the purpose of identifying the presence of (D_g_/A_g_)*, (ICT)*, and (aggregate)* emission species in the TPA-DCPP doped and DACT-II doped PS films, spectral deconvolutions were carried out as shown in Figures 3 and S10, respectively. The PL spectrum of the 0.1 wt % TPA-DCPP doped PS film and the 0.2 wt % DACT-II doped PS film can be fitted well with the Gaussian function energy fit into one emission spectrum as shown in Figures 3a and S10a, which means that no interaction occurs between the TADF molecules. Hence, the former PL spectrum can be attributed to the emission from (ICT)*. The 1 wt % TPA-DCPP doped PS film (Figure 3b) can be deconvoluted into two emission spectra, in which one has the peak at 582 nm and the other has the peak at 656 nm. The former peak with a higher fraction (64%) can be attributed to (ICT)* and the latter peak with a smaller fraction (36%) can be attributed to (aggregate)* since the peak wavelength 656 nm is close to the peak of the (aggregate)* emission 693 nm (Figure S7a). The significantly wider FWHM of the 1 wt % film spectrum (125 nm) as compared to that of the 0.1 wt % spectrum (91 nm) also supports the existence of multiple emission species in the former case. In each of the deconvoluted spectra (Figure 3c–g), there are two peaks located in the two ranges 660–697 and 738–785 nm, which can be assigned to (aggregate)* and (D_g_/A_g_)* emissions, respectively, since they are close to the value of 693 nm for (aggregate)* (Figure S7a) and the value of 758 nm for (D_g_/A_g_)* (Figure S9c). The light emission by (ICT)* disappears in the 8–100 wt % film PL spectra since the energy from (ICT)* is fully transferred to (aggregate)*. In each of all of the deconvoluted spectra of the DACT-II doped PS film (Figure S10b–f), there are two peaks located in the two ranges 482–525 and 541–561 nm, which can be assigned to (ICT)* and (D_g_/A_g_)* emissions, since they are close to the value of 479 nm for (ICT)* (Figure S10a) and 542 nm for (D_g_/A_g_)* (Figure S9b). The red shift of (aggregate)* and (D_g_/A_g_)* should be due to a better stacking orientation of the TPA/DCPP moiety for TPA-DCPP and the DAC/TRZ-moiety for DACT-II between two neighboring molecules as the concentration increases.

During the preparation of the solid film samples (guest emitters in the polystyrene host) stated above from their solutions in chlorobenzene, the guest emitter molecules are subjected to different environmental changes due to changing guest concentrations in the host. This can lead to solvatochromism of the emitter imparted by the host, leading to a slight red shift of emission,^19,38^ in addition to the primary formation of aggregates and exciplexes discussed in this work. From the case of the TPA-DCPP dopant concentration increasing from 0.1 to 100 wt % (neat film) shown in Table S3, we can observe that the FWHM value of the emission spectra significantly increases from 91 to 145 nm. The main reason for the broadening of FWHM can be attributed to the generation of aggregates and exciplex rather than from solid-state solvatochromism for which the difference in the PL spectrum FWHM between high- and low-polarity environments is about 10 nm only.^19^ The results can also be confirmed by the PLE spectra shown in Figure S7. On the other hand, the solid-state solvatochromism effect can be included in the 8–50 wt % TPA-DCPP doped polystyrene as shown in Table S3. From their emission spectral deconvolution results shown in Figure 3, the aggregation emission and exciplex emission slightly red-shift from 660 to 670 nm and 761 to 780 nm (both from 8 to 50 wt % TPA-DCPP), respectively. The aggregates and exciplex deconvolution results each have similar FWHM (from 99 to 110 nm for aggregate emission and from 117 to 138 nm for exciplex emission), which can include the contribution of solid-state solvatochromism effect as proposed by Ginsberg’s group^19^ and Adachi’s group.^38^ This minor solvatochromism contributed by the host materials could also occur in the following analysis for the hosts with various pendant hole transport moieties.

**Figure 4:**
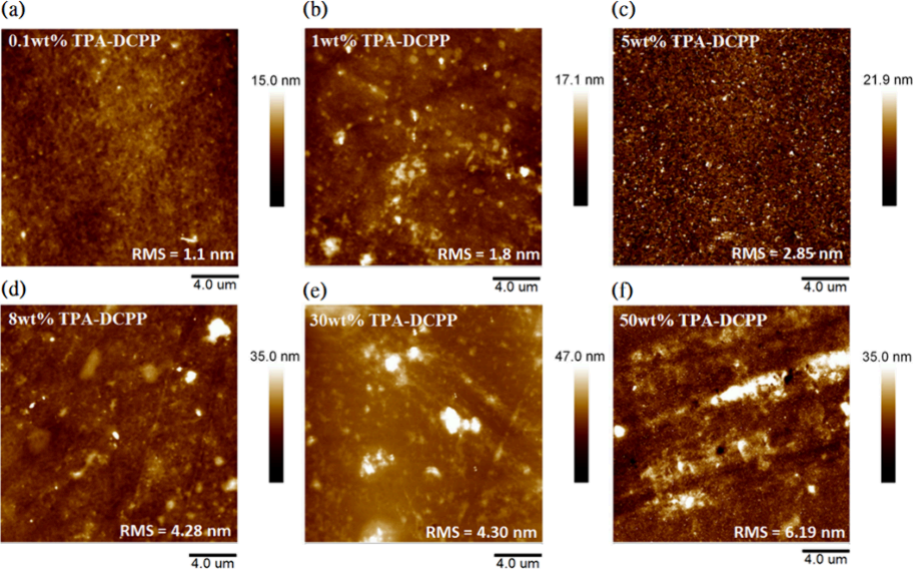
AFM images of TPA-DCPP doped polystyrene films with different dopant concentrations: (a) 0.1 wt %, (b) 1 wt % (c) 5 wt %, (d) 8 wt %, (e) 30 wt %, and (f) 50 wt %.

To further confirm the existence of aggregates in the doped PS films (0.1–50 wt %), atomic force microscopy (AFM) was used to image their surface morphology, as shown in Figure 4. The film doped with 0.1 wt % TPA-DCPP (Figure 4a) exhibits the flattest surface morphology among all of the doped PS films, with a root-mean-square (RMS) surface roughness (SR) value of 1.1 nm. As the concentration of TPA-DCPP increases, noticeable increases in aggregation particles on the surface morphology are observed, with their domain sizes also increasing with increasing doping concentration of the TPA-DCPP luminophore. Figure 4b–f illustrates the AFM images of TPA-DCPP at different concentrations of 1, 5, 8, 30, and 50 wt %; the measured RMS SR values are 1.8, 2.85, 4.28, 4.3, and 6.19 nm, respectively. Their 3D AFM images are also shown in Figure S15a–f. These results demonstrate the initiation of nanoscale aggregates at the concentration of 1 wt %, with significant phase separation occurring at the higher concentrations, which are consistent with the previously shown spectral deconvolution results (Figure 3).

**Figure 5:**
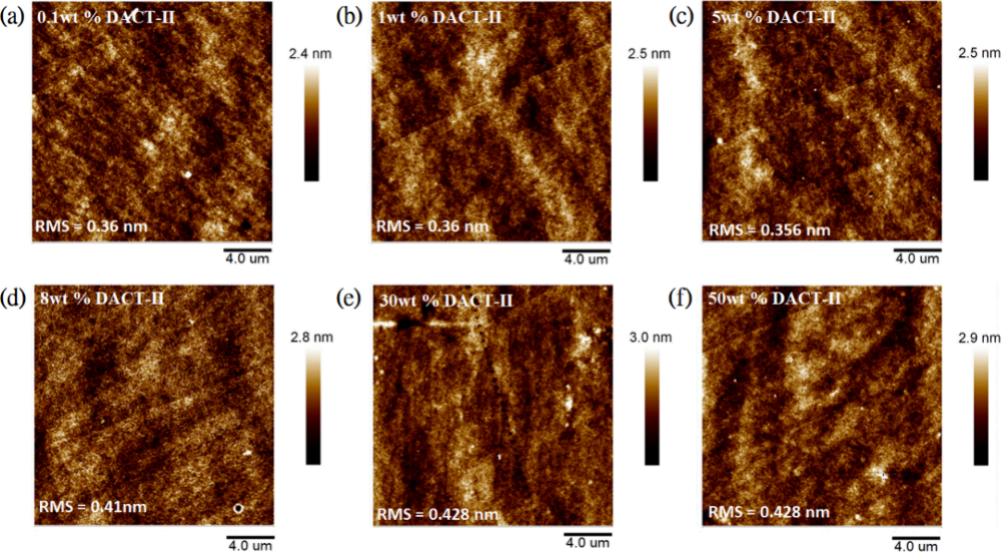
AFM images of DACT-II doped polystyrene films with different dopant concentrations: (a) 0.1 wt %, (b) 1 wt %, (c) 5 wt %, (d) 8 wt %, (e) 30 wt %, and (f) 50 wt %.

Additionally, the surface morphology of PS thin films doped with DACT-II was also measured by AFM. As shown in Figure 5a–f, the doped films of DACT-II exhibit a relatively flat surface morphology. The RMS values measured over a 20 μm area for DACT-II doping concentrations of 0.1, 1, 5, 8, 30, and 50 wt % are 0.36, 0.36, 0.356, 0.41, 0.428, and 0.428 nm, respectively. The 3D AFM images are also shown in Figure S16a–f. These RMS values are significantly lower than their corresponding TPA-DCPP doped PS films, indicating that using DACT-II as a guest material has less tendency to form aggregates, which is also consistent with the spectral deconvolution results as discussed earlier (Figure S10).

### 3.2 Donor–Acceptor Interaction between D-Polymer Host and TADF-Guest in a Simulated Donor/Acceptor System

**Figure 6:**
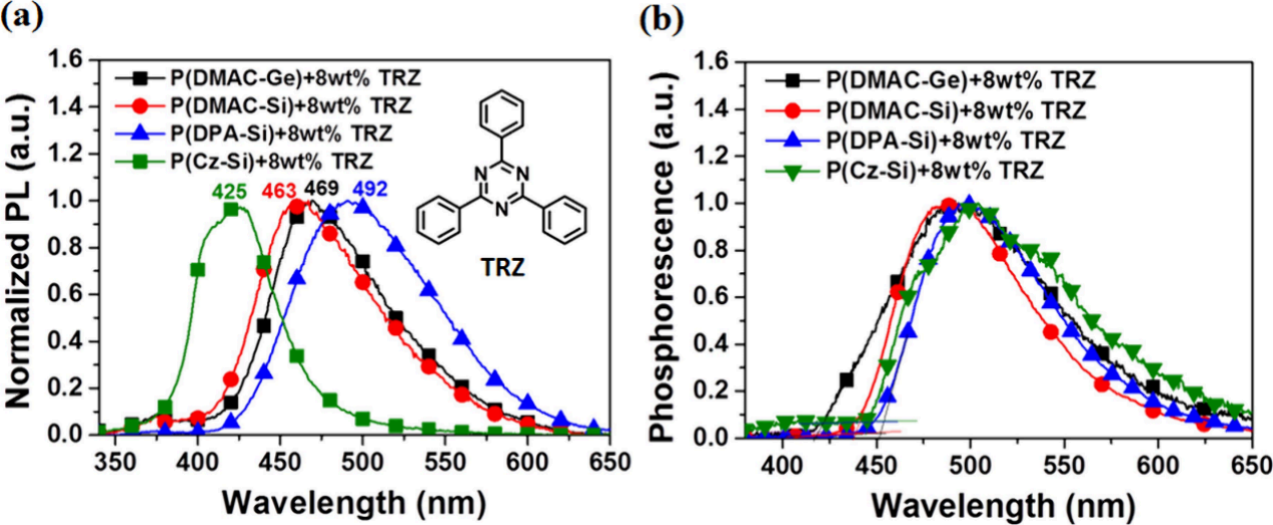
PL spectra of (a) 8 wt % TRZ doped in the D-polymer host films at RT. (b) Phosphorescence spectra at 77 K with 1 ms delay time and for 8 wt % TRZ doped in the D-polymer host films.

Since the D–A interactions between host and guest molecules are rather complicated, we use the electron-accepting molecules DCPP and TRZ as the guests (see Scheme 1c for their chemical structures), which have the same chemical structures as the acceptor parts of DMAC-TRZ or DACT-II and TPA-DCPP (Scheme 1b), to simulate the host–guest interactions in the sB/G/R emitters with D-polymer hosts. The PL spectral maximum emissions (λ_PL,max_) of TRZ doped P(DMAC-Ge), P(DMAC-Si), P(DPA-Si), and P(Cz-Si) films are at 469, 463, 492, and 425 nm (Figure 6a), and those of DCPP doped D-polymer films are at 610, 607, 536, and 760 (Figure S11), which appear clearly red-shifted relative to the emission peaks of 395, 405, 396, and 402 nm for the neat films of P(DMAC-Ge), P(DMAC-Si), P(DPA-Si), and P(Cz-Si), respectively (Figure S11). Based on the featurelessness emission spectra in these blend films along with the considerable red-shift relative to the emissions from the neat D-polymer films, the variations of the PL emissions from these blend films can be attributed to the formation of D/A exciplexes in the simulated systems, which is designated as (D/A)*.^32,39^

**Figure 7:**
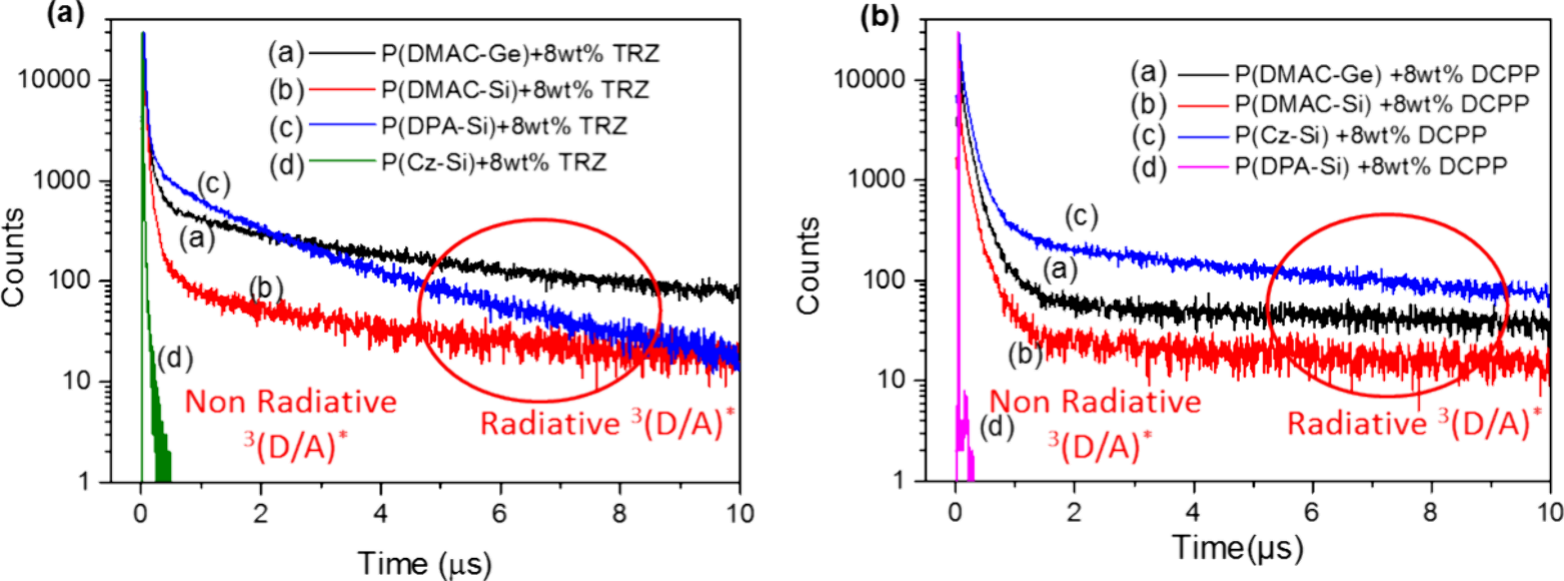
Transient PL decays at RT (monitored at the PL peak) for 8 wt % (a) TRZ and (b) DCPP doped in the D-polymer host films.

The phosphorescence spectra of D-polymer films doped with 8 wt % TRZ and DCPP were measured at 77 K with a 1 ms delay time (Figures 6b and S12). In order to identify the emission spectra of (D/A)* in 8 wt % DCPP doped P(DMAC-Ge) and P(DMAC-Si) films (Figure S12a,b), a spectral deduction of the D-polymer film was carried out for each phosphorescence spectrum of the 8 wt % DCPP doped D-polymer host. This deduction considered the exclusion of emissions from the D-polymer host (Figure S14), and as a result, the remaining emission spectrum was assigned to the phosphorescence of (D,A)*. On the other hand, the 8 wt % DCPP doped P(DPA-Si) film shows only the phosphorescence of P(DPA-Si) (Figure S12c), indicating that the phosphorescence of (D/A)* in the 8 wt % DCPP doped P(DPA-Si) film is too weak to be observed. The T_1_ levels estimated from 8 wt % TRZ and DCPP doped D-polymer films' onset values at the short wavelength side are 2.96, 2.83, 2.74, and 2.77 eV for 8 wt % TRZ doped P(DMAC-Ge), P(DMAC-Si), P(DPA-Si), and P(Cz-Si) films and 2.25, 2.23, and 2.54 eV for DCPP doped P(DMAC-Ge), P(DMAC-Si), and P(Cz-Si) films, respectively (Table 1), all of which can be assigned as the T_1_ levels of their (D/A)*. The doped P(DMAC-Ge) films show higher T_1_ levels of (D/A)* than that of the doped P(DMAC-Si) films due to the smaller extent of π-electron delocalization affected by the size effect and the orbital interaction of the central atom.^40^ Based on these results, it is conceivable that the T_1_ levels of ^3^(D_h_/A_g_)* from the sB/G/R TADF doped D-polymer films should be close to those of (D/A)* from the simulated TRZ/DCPP doped D-polymer system films because the electron acceptor moieties of the sB/G/R TADF emitters, DMAC-TRZ, DACT-II, and TPA-DCPP (Scheme 1b), are identical to TRZ and DCPP. Therefore, the T_1_ level of ^3^(D/A)* from the D-polymer host/TRZ and D-polymer host/DCPP interaction provides a crucial basis for exploring the triplet energy transfer among ^3^(D_h_/A_g_)*, (aggregate)*, and ^3^(ICT)* of the TADF guest. Follow-up transient PL emission decay measurements for 8 wt % TRZ/DCPP doped D-polymer films at room temperature are carried out (Figure 7). Their exciton lifetimes (τ_1_ and τ_2_) and emission fractions estimated from two exponentials' fit are listed in Table 1. As can be seen, the PL decays of 8 wt % TRZ doped P(DMAC-Ge), P(DMAC-Si), and P(DPA-Si) films and 8 wt % DCPP doped P(DMAC-Ge), P(DMAC-Si), and P(Cz-Si) films show PF (τ_1_ = 25.1–80.7 ns) and delay fluorescence (DF) (τ_2_ = 1.8–5.3 μs) characteristics due to small Δ*E*_ST_ (0.04–0.15 eV) between ^1^(D/A)* and ^3^(D/A)* except for the system of the 8 wt % TRZ doped P(Cz-Si) film. For this system, only PF (τ_1_ = 3.0 ns and τ_2_ = 12.1 ns) but no DF characteristics are observed; these τ_1_ and τ_2_ should be attributed to the intrinsic host and ^1^(D_h_/A_g_)* fluorescence luminance (FL), respectively, which are close to the general FL lifetimes of D/A exciplexes (about 10–50 ns).^41,42^ As to its τ_2_ (12.1 ns), it is much shorter than the general decay emission τ_2_ (1–5 μs); thus, this system can be considered as having no TADF characteristic. The PL spectral maximum emission (λ_PL,max_) is at 425 nm for TRZ doped P(Cz-Si) films (Figure S13a), which appear clearly red-shifted relative to the emission peaks of 402 nm for the neat films of P(Cz-Si) and 399 nm for the TRZ. The phosphorescence spectra of TRZ, P(Cz-Si), and its 8 wt % TRZ doped films are measured at 77 K with a 1 ms delay time as shown in Figure S13b. The PhF spectrum of the 8 wt % Trz doped P(Cz-Si) film shows clear red-shifting relative to the emission peaks of 481 nm for the neat film of TRZ. The T_1_ level estimated from its onset value at the short wavelength side is 2.77 eV, which is close to that of the P(Cz-Si) neat film value (2.76 eV). Therefore, the large Δ*E*_ST_ (0.45 eV) of the 8 wt % TRZ doped P(Cz-Si) film is correct and can be explained by the competition between two different up-conversions of triplet excitons to singlet-state mechanisms: TTA and TADF as proposed by Monkman’s group.^43^ In this case, the exciplex emission is dominated by the TTA mechanism, which leads to weak TADF characteristics in transient PL decay at room temperature (Figure 7a).

**Table 1:** Exciton Lifetimes, Component Fractions, and S_1_, T_1_, and Δ*E*_ST_ Values for the D-Polymer Host Doped with 8 wt % TRZ/DCPP Films

Compounds	τ_1_^a^ (ns)	A_1_%^a^	τ_2_^a^ (ns)	A_2_%^a^	χ^2^^b^	S_1_^c^ (eV)	T_1_^d^ (eV)	Δ*E*_ST_^e^ (eV)	type of ^3^(D/A)*^f^
P(DMAC-Ge)+8 wt % TRZ	25.1	42.2	2878.2	57.8	1.24	2.92	2.96	0.04	rad.
P(DMAC-Si)+8 wt % TRZ	35.0	80.2	2188.6	19.8	1.31	2.98	2.83	0.15	rad.
P(DPA-Si)+8 wt % TRZ	34.4	38.7	1780.2	61.3	1.51	2.89	2.74	0.15	rad.
P(Cz-Si)+8 wt % TRZ	3.0	24.8	12.1	75.2	1.77	3.22	2.77	0.45	NR^g^
P(DMAC-Ge)+8 wt % DCPP	80.7	84.7	5334.6	15.3	1.12	2.29	2.25	0.04	rad.
P(DMAC-Si)+8 wt % DCPP	73.8	91.2	2188.6	8.8	1.29	2.28	2.23	0.05	rad.
P(DPA-Si)+8 wt % DCPP	0	0	30.2	100.0	1.21	1.71	2.76	1.05-	NR^h^
P(Cz-Si)+8 wt % DCPP	64.6	74.5	3313.1	25.5	1.47	2.63	2.54	0.09	rad.

a These exciton lifetimes and component fractions were determined from the transient PL decays and monitored at their maximum PL emissions.

b Chi-square values for exponential fitting of the decay curves.

c Estimated from the onset of the PL spectrum.

d Estimated from the onset of the phosphorescence spectrum.

e Calculated from the difference between S_1_ and T_1_.

f Rad. and NR mean radiative and nonradiative processes, respectively.

g As its τ2 (12.1 ns) is much shorter than the general decay emission τ2 (1–5 μs), it can be considered as having no TADF characteristic.

h As its T_1_ > S_1_, it means this system has no TADF characteristic.

For the PL decay of the 8 wt % DCPP doped P(DPA-Si) film, it shows PF (τ_1_ = 0 ns and τ_2_ = 30.2 ns), where τ_2_ was contributed by the ^1^(D/A)* FLs. As its τ_2_ (30.2 ns) is much shorter than the general decay emission τ_2_ (1–5 μs), it can be considered as having no TADF characteristic. The T_1_ level estimated from the Ph spectrum of 8 wt % DCPP doped P(DPA-Si) film onset values at the short wavelength side in Figure S12c is 2.76 eV, which is close to that of the P(DPA-Si) neat film value (2.78 eV). Thus, the Δ*E*_ST_ for P(DPA-Si) doped with 8 wt % DCPP film has the negative value −1.05 eV, which means that the exciplex is not a TADF. The transient PL result in Figure 7b also shows that the DCCP doped P(DPA-Si) film does not exhibit TADF characteristics. From both of these experimental results, we attributed it to the fact that the exciplex for the DCPP doped P(DPA-Si) film is also dominated by the TTA mechanism,^43^ similar to that of the TRZ doped P(Cz-Si) film as mentioned in the above paragraph.

**Figure 8:**
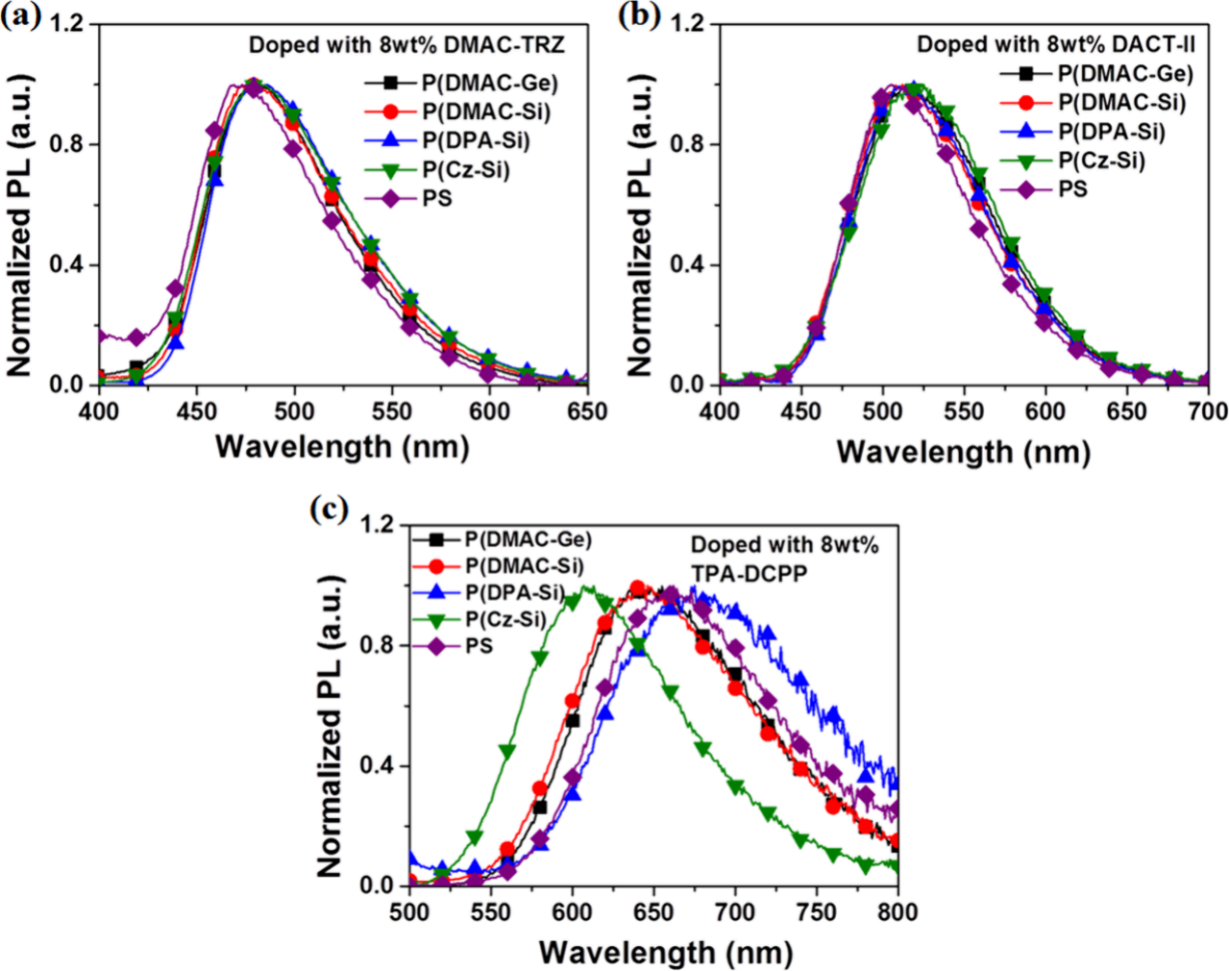
PL spectra of the D-polymer doped with 8 wt % sB/G/R TADF films: (a) DMAC-TRZ, (b) DACT-II, and (c) TPA-DCPP as TADF emitters.

Up to this stage, we have already confirmed the presence of (D_g_/A_g_)* and (aggregate)* in the 8 wt % TPA-DCPP doped PS film; similarly, it should also exist in the doped films with the present D-polymers as hosts. In addition, (D_h_/A_g_)* could exist too. To explore if the emitting species (D_h_/A_g_)*, (aggregate)*, and (D_g_/A_g_)*, in addition to (ICT)*, form in these host/guest systems, we choose 8 wt % TPA-DCPP doped D-polymer films as the first case for study because there are significant PL spectral differences (including λ_PL, max_ and FWHM) as compared to the systems with DMAC-TRZ and DACT-II as guests, which give sky-blue emissions (λ_PL,max_ ∼ 480 nm) and green emissions (λ_PL,max_ ∼ 510 nm), respectively (Figure 8a,b). Their FWHMs are similar in both sB and G systems, ∼80 nm for the former and ∼95 nm for the latter (Table 2). For 8 wt % TPA-DCPP doped D-polymer films, their PL differences are large, allowing easy identification of their (D_h_/A_g_)* emissions (Figure 8c). On the contrary, the PL differences in 8 wt % DMAC-TRZ (Figure 8a) and DACT-II doped D-polymer films (Figure 8b) are small, which leads to difficulty in identifying the emission from (D_h_/A_g_)*. The small difference in PL spectra in the sB and G systems might indicate that the emission spectra of (D_h_/A_g_)* could probably highly overlap with or be close to their corresponding (ICT)* emission spectra. For that, the identification of (D_h_/A_g_)* in the sB and G doped systems via transient PL measurements will be carried out after the exploration of (D_h_/A_g_)* in the R guest doped D-polymer system.

**Table 2:** PL Parameters of the 8 wt % sB/G/R TADF Guest Doped D-Polymer Films

emitter	polymer	λ_PL,max_ (nm)	FWHM (nm)
DMAC-TRZ	P(DMAC-Ge)	481	77
	P(DMAC-Si)	479	79
	P(DPA-Si)	483	83
	P(Cz-Si)	482	84
DACT-II	P(DMAC-Ge)	515	96
	P(DMAC-Si)	512	95
	P(DPA-Si)	514	94
	P(Cz-Si)	521	98
TPA-DCPP	P(DMAC-Ge)	646	130
	P(DMAC-Si)	644	133
	P(DPA-Si)	677	153
	P(Cz-Si)	609	115

**Figure 9:**
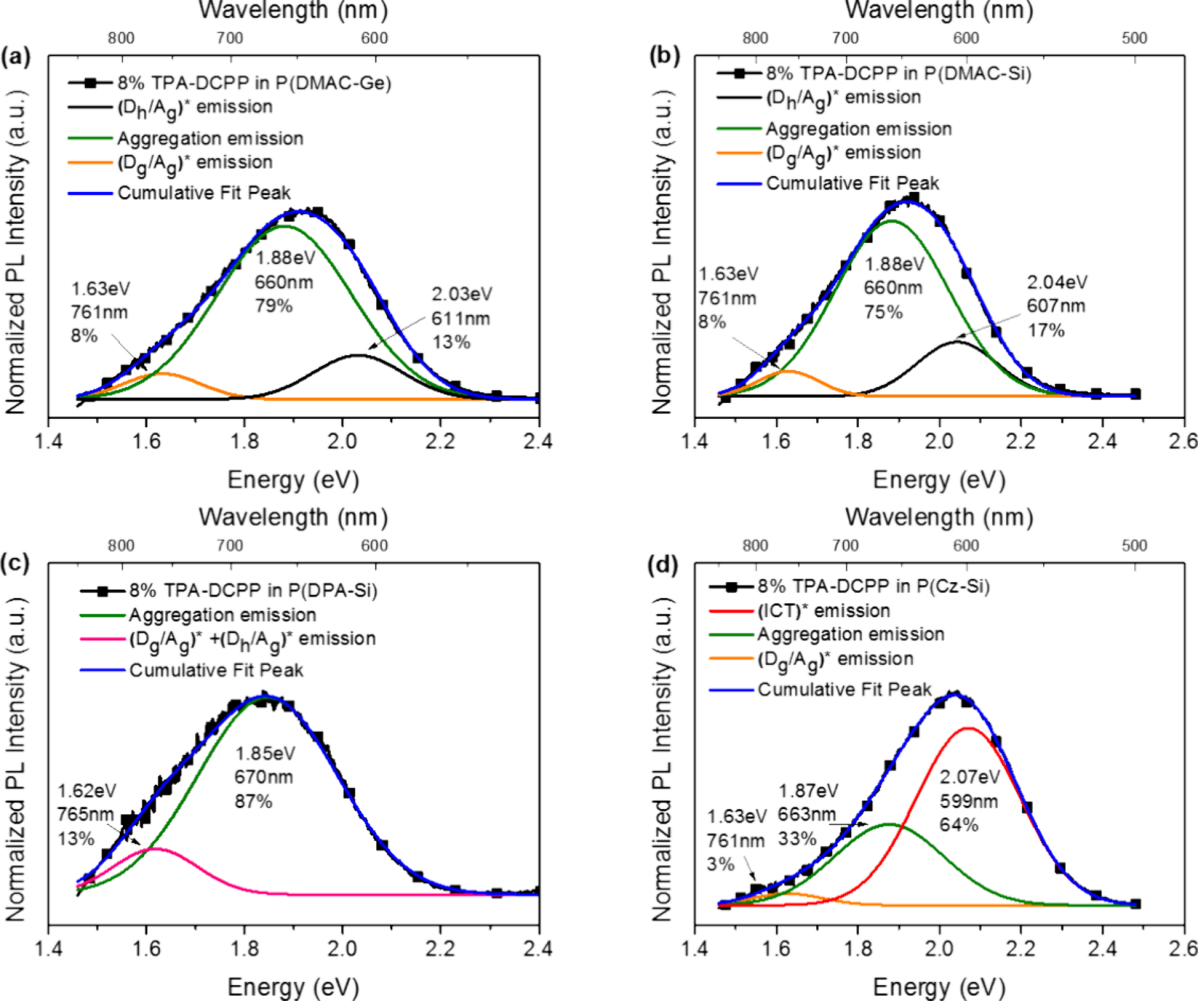
Deconvoluted plots and fractions from the PL spectra of 8 wt % TPA-DCPP doped in (a) P(DMAC-Ge), (b) P(DMAC-Si), (c) P(DPA-Si), and (d) P(Cz-Si) films.

To identify the emission species in these doped D-polymer hosts, we perform three-component spectral deconvolution for each spectrum of the 8 wt % TPA-DCPP doped D-polymer host films to estimate possible emission spectra and fractions of (D_h_/A_g_)*, (D_g_/A_g_)*, (aggregate)*, and (ICT)* emissions. As shown in Figure 9a–d, in each of all of the deconvoluted spectra, there are two peaks located in the ranges of 660–670 and 761 nm, which can be assigned to (aggregate)* and (D_g_/A_g_)* emissions, respectively, since they are close to the value 660 nm for (aggregate)* and have the same value 761 nm for (D_g_/A_g_)* in the 8 wt % TPA-DCPP doped PS film, respectively (Figure S9c). Additionally, from the AFM images in Figure S17a,c, it can be seen that P(DMAC-Ge) has a flat morphology, with an RMS value of 1.02 nm. In contrast, after doping with 8 wt % TPA-DCPP, the presence of aggregates is evident in the images of Figure S17b,d, with an RMS value of 4.77 nm. This result confirms the aforementioned deconvolution spectral analysis and is consistent with the previous analysis of interacting emitting species in the PS/TADF-guest system. However, the 761 nm peak of the TPA-DCPP doped P(DPA-Si) film can also be attributed to (D_h_/A_g_)* due to the high overlap of (D_g_/A_g_)* emission at 761 nm and (D_h_/A_g_)* emission at 760 nm. The third peaks of TPA-DCPP doped P(DMAC-Ge) and P(DMAC-Si) films at 610 and 607 nm can be assigned to (D_h_/A_g_)* as they are the same as the values of the emission peaks of exciplexes formed by P(DMAC-Ge) and P(DMAC-Si) with the acceptor DCPP. The (D_h_/A_g_)* emission cannot be seen in the TPA-DCPP doped P(Cz-Si) film since the (D_h_/A_g_)* emission spectrum overlaps with the absorption spectrum of (ICT). Hence, the third peak of the TPA-DCPP doped P(Cz-Si) film can be attributed to (ICT)*.

In addition, the (D_g_/A_g_)* emissions estimated by the spectral deconvolution give the fractions and emission peaks: 8% (761 nm), 8% (761 nm), 16% (761 nm), and 3% (761 nm) for the 8 wt % TPA-DCPP doped P(DMAC-Ge), P(DMAC-Si), P(DPA-Si), and P(Cz-Si) films, respectively (Table S5). All of the (D_g_/A_g_)* except for the TPA-DCPP doped P(DPA-Si) film show much lower fractions than that of the doped PS film (16%) (Table S3), which can be attributed to a competing formation of (D_h_/A_g_)* that could lead to a reduced chance for the formation of (D_g_/A_g_)*. On the other hand, the deconvoluted peak at 761 nm of the TPA-DCPP doped P(DPA-Si) film (13%) has a higher fraction than that of the doped PS film since it can be considered as a combination of the contributions from (D_h_/A_g_)* and (D_g_/A_g_)*.

To ascertain the analysis for the emissions from the exciplexes in addition to (ICT)*, we further measure transient PL decays of 8 wt % TPA-DCPP doped D-polymer host films at room temperature (Figure S18c). All of the decays fit well with triexponential lifetime components, and the lifetimes and their corresponding emission fractions are listed in Table 3, attributed to ^1^(ICT)* and (aggregate)* (τ_1_ = 10.3–22.8 ns), ^1^(D_h_/A_g_)* (τ_2_ = 57.5–96.7 ns), and the mixed DF species, ^3^(ICT)* and ^3^(D_h_/A_g_)* (τ_3_ = 0.2–1.3 μs). The time-scale of τ_2_ for ^1^(D_h_/A_g_)* is slightly longer than the general exciplex lifetimes (about 10–50 ns)^41,42,44^ since the formation of the intermolecular exciplex TADF species may further affect its neighboring molecule (here it should be the host), which produces multiple back and forth intersystem crossings between nearly resonant singlet states, leading to the extended exciplex lifetimes (∼100 ns).^32,39^ As shown in Table 3, for the 8 wt % TPA-DCPP doped P(Cz-Si) film, its τ_1_ and τ_3_ (τ_1_ ∼ 10 ns and τ_3_ ∼ 0.2 μs) values are significantly shorter than the others (τ_1_ ∼ 20 ns and τ_3_ ∼ 1.2 μs), which is due to the additional exciton decay process induced by energy transfer from (ICT)* to (aggregate)*.^26,45^

**Table 3:** Photoluminescence Quantum Yields (PLQYs) and Three Exponential Fittings of Exciton Lifetimes of 8 wt % DMAC-TRZ, DACT-II, and TPA-DCPP Film Doped D-Polymer Films

emitter	D-polymer	PLQY (%)	τ_1_ (ns)^a^	A_1_%^a^	τ_2_ (ns)^a^	A_2_%^a^	τ_3_ (μs)^a^	A_3_%^a^	χ^2^^b^
TPA-DCPP	P(DMAC-Ge)	23	21.1	45.4	90.4	52.2	1.2	2.4	1.40
	P(DMAC-Si)	21	22.8	47.0	96.0	50.7	1.3	2.3	1.29
	P(DPA-Si)	6	19.8	45.6	96.7	50.2	1.2	4.2	1.56
	P(Cz-Si)	61	10.3	68.2	57.5	27.3	0.2	4.5	0.68
DMAC-TRZ	P(DMAC-Ge)	91	16.0	51.9	126.6	8.1	2.6	40.0	1.16
	P(DMAC-Si)	87	15.7	59.3	102.1	7.4	2.5	33.3	1.13
	P(DPA-Si)	71	17.6	47.0	98.0	12.1	2.6	40.9	1.34
	P(Cz-Si)	67	13.9	77.9	83.6	5.7	2.2	16.4	1.21
DACT-II	P(DMAC-Ge)	99	9.6	77.7	54.4	8.6	1.7	13.7	1.30
	P(DMAC-Si)	98	9.4	81.2	52.1	7.9	1.7	10.9	1.17
	P(DPA-Si)	90	10.7	72.6	65.7	10.7	1.8	16.7	1.28
	P(Cz-Si)	61	9.7	85.9	51.8	7.5	1.5	6.6	1.04

a These exciton lifetimes are determined from the transient PL decays (Figure S18) and monitored at their maximum PL emissions.

b Chi-square values for exponential fitting of the decay curves.

As the PL spectral variations among 8 wt % sB and G TADF doped D-polymers are not large enough for carrying out spectral deconvolution, the transient PL decays, however, would not restrict us from analyzing their exciton lifetimes. Their transient decay measurements are carried out (Figure S18a,b), and their characteristic parameters are listed in Table 3. The τ_1_, τ_2_, and τ_3_ are assigned to ^1^(ICT)*, ^1^(D_h_/A_g_)*, and the mixed DF species, ^3^(ICT)* and ^3^(D_h_/A_g_)*, respectively. Their τ_2_ values are similar to the above 8 wt % TPA-DCPP doped ones but are slightly longer than those of the general exciplex (about 10–50 ns). The fraction of τ_2_ for ^1^(D_h_/A_g_)* in the DMAC-TRZ doped ones (5.7–12.1%) and the DACT-II doped ones (7.5–10.7%) is obviously lower than that of the TPA-DCPP doped D-polymer films (27.3–52.2%) (Table 3). Such a high fraction of ^1^(D_h_/A_g_)* in the 8 wt % TPA-DCPP doped D-polymer films also reveals why this (D_h_/A_g_)* can be clearly identified in its PL spectrum. The lifetimes of τ_3_ in the 8 wt % sky-blue and green TADF doped P(Cz-Si) films (2.2 μs for DMAC-TRZ and 1.5 μs for DACT-II) are slightly shorter than the others (2.5–2.6 μs for DMAC-TRZ and 1.7–1.8 for DACT-II) due to the formation of NR ^3^(D_h_/A_g_)*, based on the previous simulated result of the P(Cz-Si)/TRZ film (Figure 7a and Table 1). In addition, the 8 wt % DMAC-TRZ and DACT-II doped P(Cz-Si) films show significantly lower τ_3_ fractions (16.4 and 6.6%) as compared to the ones using DPA- and DMAC-based hosts (33.3–40.9 and 10.9–16.7%), also suggesting the formation of NR ^3^(D_h_/A_g_)* that leads to the reduced fraction of ^3^(ICT)* (A_3_ as 16.4 and 6.6% in Table 3). Therefore, it is conceivable that the lifetime of τ_3_ and its fraction would decrease as NR ^3^(D_h_/A_g_)* is formed, thus lowering the PLQY of (ICT)*. This point will be further discussed in the following section.

### 3.3 Influence of exciplexes ^3^(D_h_/A_g_)* on the Photoluminescence Efficiency of sB/G/R TADF in the D-Polymer

**Figure 10:**
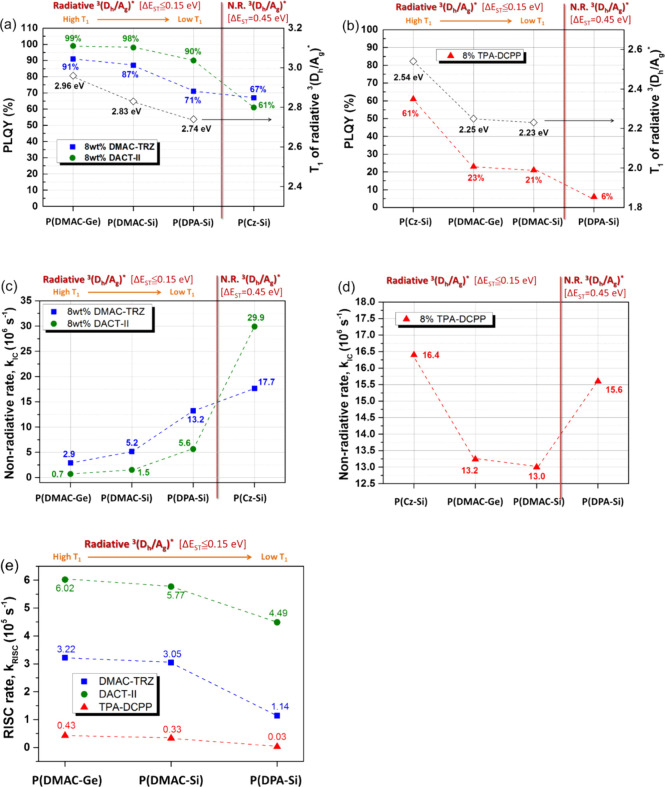
Influence of the exciplexes ^3^(D_h_/A_g_)* on the optical properties of TADF emitters: PLQY values for 8 wt % (a) sB/G and (b) R TADF emitters doped in the D-polymer hosts. Nonradiative rates for 8 wt % (c) sB/G and (d) R TADF emitters doped in the D-polymer hosts. (e) RISC rates of the 8 wt % sB/G/R TADF emitters doped in P(DMAC-Ge), P(DMAC-Si), and P(DPA-Si) films. Note: (1) T_1_ of radiative ^3^(D_h_/A_g_)* and their Δ*E*_ST_ between ^1^(D_h_/A_g_)* and ^3^(D_h_/A_g_)* are based on the measurements in Table 1 for the simulated D-polymer/TRZ system; (2) assignment of ^3^(D_h_/A_g_)* as NR ^3^(D_h_/A_g_)* for sB/G doped P(Cz-Si) and R doped P(DPA-Si) systems is taken from the result in Figure S14.

To explore the influence of ^3^(D_h_/A_g_)* formation including radiative and NR ^3^(D_h_/A_g_)* and T_1_ level of ^3^(D_h_/A_g_)* on the emission efficiency of the TADF guest, we measure the PLQY (shown in Table 3) and calculate the nonradiative internal conversion rate constant (k_IC_) and the RISC rate constant (k_RISC_) from the data of PLQY and exciton lifetimes of prompt and delayed emissions (Tables S6 and S7) for the D-polymer host doped with 8 wt % sB/G/R TADF emitter films. Their characteristic values of PLQYs, k_IC_, and k_RISC_ are displayed in Figure 10a–e. As shown in Figure 10a, the PLQYs for sB/G/R TADF guest doped P(DMAC-Ge), P(DMAC-Si), P(DPA-Si), and P(Cz-Si) films are 91, 87, 71, and 67% for DMAC-TRZ (sky-blue); 99, 98, 90, and 61% for DACT-II (green); and 23, 21, 6, and 61% for TPA-DCPP (red), respectively. Among the TADF guests, the red ones give much lower PLQYs than the sky-blue and green ones due to the intrinsically low PLQY of TPA-DCPP in the film state (14%).^36^ However, its T_1_ of the radiative ^3^(D_h_/A_g_)* (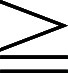
2.83 eV) is higher than those of the sky-blue and green guests (2.77 eV for DMAC-TRZ and 2.70 eV for DACT-II)^34,35^ in the sB/G doped P(DMAC-Ge) and P(DMAC-Si) films (based on the simulated D-polymer/TRZ systems in Table 1), which is beneficial for obtaining higher PLQYs due to the feasible energy transfer from ^3^(D_h_/A_g_)* (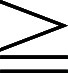
2.83 eV) to ^3^(ICT)*. However, for the radiative ^3^(D_h_/A_g_)* with lower T_1_ (2.74 eV) and the NR ^3^(D_h_/A_g_)* in the sB/G doped P(DPA-Si) and P(Cz-Si) films, respectively, both have lower PLQYs due to the self-emission of ^3^(D_h_/A_g_)* for the former, which leads to no energy gain by the ^3^(ICT)* of the guest, and nonradiative triplet energy loss for the latter. Similarly, we can expect that the low PLQY in the R-doped P(DPA-Si) film could be caused by the formation of ^3^(D_h_/A_g_)*, which is nonradiative, and the energy is lower than that of TPA-DCPP, as can be inferred from its emission energy (λ_PL,max_ at 765 nm) of (D_h_/A_g_)* being lower than that of (ICT)* from TPA-DCPP (λ_PL,max_ at 670 nm) (Figure 10d), which therefore leads to unallowed energy transfer from (D_h_/A_g_)* to (ICT)* and nonradiative triplet energy loss. The exceptionally high PLQY of R-doped P(Cz-Si) is explained next.

**Scheme 4:**
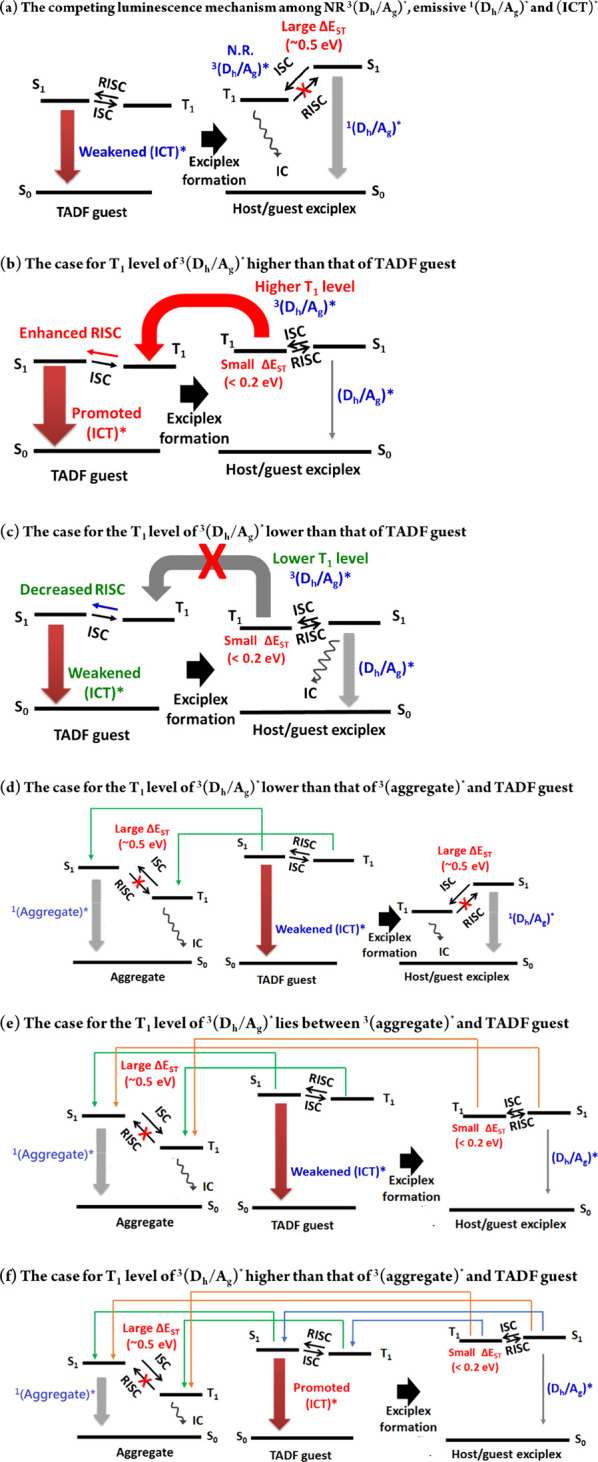
Schematic Illustration of the Mechanisms between the D-Polymer Host and TADF Guest Proposed in This Study The cases of competing luminescence mechanism among NR ^3^(D_h_/A_g_)*, ^1^(D_h_/A_g_)*, and ICT: (a) NR ^3^(D_h_/A_g_)* (Δ*E*_ST_ ≈ 0.5 eV) forms, (b) T_1_ level of ^3^(D_h_/A_g_)* higher than that of ^3^(ICT)*, and (c) T_1_ level of ^3^(D_h_/A_g_)* is lower than that of ^3^(ICT)*. The cases of competing luminescence mechanism among NR ^3^(D_h_/A_g_)*, ^1^(D_h_/A_g_)*, ^3^(aggregate)*, and ICT: (d) NR ^3^(D_h_/Ag)* has lower energy than ^3^(ICT)* and ^3^(aggregate)*, (e) T_1_ level of ^3^(D_h_/Ag)* falls in between ^3^(ICT)* and ^3^(aggregate)*, and (f) T_1_ level of ^3^(D_h_/Ag)* is higher than that of ^3^(ICT)* and ^3^(aggregate)*. (IC is the nonradiative internal conversion, S_0_ is the ground state, and S_1_ and T_1_ represent singlet and triplet levels, respectively.)

All of the PLQYs (Figure 10a,b) show high correspondence to the k_IC_ values of the TADF in the sB/G/R TADF doped D-polymer films (Figure 10c,d), in which the PLQY increases as the k_IC_ decreases except for the red doped P(Cz-Si) film, for which its PLQY is much higher than the others even though its k_IC_ value is large. On the one hand, the emission of the red doped P(Cz-Si) film is dominated by (ICT)*, which has a higher PLQY (84%) than the (aggregate)* (22%). On the other hand, the ^3^(D_h_/A_g_)* shows a higher T_1_ than (ICT)*, which is favorable for energy transfer from ^3^(D_h_/A_g_)* to (ICT)*. However, the energy transfer process could also proceed between (ICT)* and (aggregate)* as shown in Scheme 4, which induces additional exciton decay and thus increases the k_IC_ value.^26,45^ Here, the decreased k_IC_ value means less nonradiative decay that is favorable to achieve high PLQY. The sB/G/R-doped P(DMAC-Ge) and P(DMAC-Si) films show lower k_IC_ than the sB/G/R-doped P(DPA-Si) films due to their ^3^(D_h_/A_g_)* with higher T_1_. However, the sB/G/R-doped P(Cz-Si) films show higher k_IC_ than the others due to the formation of NR ^3^(D_h_/A_g_)*. One may argue that other factors may cause a reduction in the PLQY of the TADF guest in our D-polymer hosts, such as the shallow HOMO level and large excited-state dipole moment; both would lead to some extent of exciton quenching according to previous reports,^15,23−25^ and these concerns are excluded here as discussed in Section S3.4 of the SI. Therefore, we do ensure that the PLQY of (ICT)* is affected by the T_1_ level of the ^3^(D_h_/A_g_)*.

For further confirmation of if the high PLQY in the doped D-polymer films can be attributed to the radiative ^3^(D_h_/A_g_)* with high T_1_ that leads to the increased RISC rate of the TADF guest, the relationship between the T_1_ level of radiative ^3^(D_h_/A_g_)* and the RISC rate for 8 wt % sB/G/R TADF emitter doped P(DMAC-Ge), P(DMAC-Si), and P(DPA-Si) films is shown in Figure 10e. Interestingly, their k_RISC_ in these sB/G/R TADF emitter doped films all show the same sequence of P(DMAC-Ge)> P(DMAC-Si)> P(DPA-Si), where the k_RISC_ values for the doped P(DMAC-Ge) and P(DMAC-Si) films with higher T_1_ of ^3^(D_h_/A_g_)* are significantly larger than the doped P(DPA-Si) with lower T_1_ of ^3^(D_h_/A_g_)*, based on the results of the simulated D-polymer/TRZ and D-polymer/DCPP systems (Table 1). Moreover, all of the k_RISC_ values for the doped P(DMAC-Ge) films in sB/G/R emissions were slightly higher than those of the doped P(DMAC-Si) films (Figure 10e); this difference could have originated from the more intense external heavy-atom effect for P(DMAC-Ge) than P(DMAC-Si) imparted by the heavy-atom Ge in the former.^22^

Based on the results above, we realize that the formation of (D_h_/A_g_)* is unavoidable in the host/TADF-guest system. For highly efficient (ICT)* emission, it is preferred to have radiative ^3^(D_h_/A_g_)* with a higher T_1_ level than that of (ICT)*, but it is undesirable to have NR ^3^(D_h_/A_g_)* and radiative ^3^(D_h_/A_g_)* with a lower T_1_ level than that of (ICT)*. Among the D-polymers, P(DMAC-Ge) is the most suitable host for sB/G/R TADF emitters due to its ^3^(D_h_/A_g_)* with high T_1_.

### 3.4 Emission Mechanisms in the Presence of D-Polymer Host/TADF-Guest Interaction

Based on the understanding up to this stage on the interactions between the D-polymer hosts and sB/G/R TADF guests in the host/guest system, we propose three types (a–c) of emission mechanisms for the sB/G TADF guest system and three other types (d–f) for the R TADF guest system as follows (Scheme 4). The presence of the exciplexes can either weaken or promote the TADF emission of the guest depending on the type of exciplex formed in the emitting process. In type (a), nonradiative triplet exciplex NR ^3^(D_h_/A_g_)* (Δ*E*_ST_ ≈ 0.45 eV) forms, which increases the rate of the nonradiative IC process and promotes ^1^(D_h_/A_g_)* emission, both leading to the decreased DF component of (ICT)* and thereby weakening the TADF emission. It occurs when the Cz-based polymer as a host interacts with sB/G TADF guests. In other cases, some specific host–guest interactions can lead to the formation of DF exciplexes (such as sB/G TADF emitter doped P(DMAC-Ge), P(DMAC-Si), and P(DPA-Si) films), which are of radiative triplet exciplexes ^3^(D_h_/A_g_)* (Δ*E*_ST_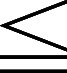 0.15 eV), and it allows or disallows energy transfer from ^3^(D_h_/A_g_)* to ^3^(ICT)* of the TADF guest depending on their T_1_ levels. In type (b), the T_1_ level of ^3^(D_h_/A_g_)* is higher than that of ^3^(ICT)*, which would lead to a promotion of the exciton population in ^3^(ICT)* through energy transfer from ^3^(D_h_/A_g_)*, leading to a promotion of the RISC rate of the guest from T_1_ to S_1_, and thus enhancing the TADF emission of (ICT)*. It occurs in the case of sB/G TADF emitter doped P(DMAC-Ge) and P(DMAC-Si). In type (c), the T_1_ level of ^3^(D_h_/A_g_)* is lower than that of ^3^(ICT)*, which would result in unallowed energy transfer from the former to the latter, leading to ^3^(D_h_/A_g_)* self-emission and unpromoted TADF emission. It occurs in the case of sB/G TADF emitter doped P(DPA-Si) films. Among these three types, type (b) is preferable for high PLQY. As for the R TADF guest/D-polymer host system, the interaction is more complex due to the existence of (aggregate)* and (D_g_/A_g_)*. The ^3^(aggregate)* and ^3^(D_g_/A_g_)* of TPA-DCPP are nonradiative and have a lower energy than ^3^(ICT)*, resulting in energy transfer from (ICT)* to (aggregate)*, which has a much lower PLQY than (ICT)*. Hence, the PLQYs of the R TADF guest/D-polymer host system are much lower than the sB/G ones. In type (d), nonradiative triplet exciplex NR ^3^(D_h_/A_g_)* has lower energy than ^3^(ICT)* and ^3^(aggregate)*, which does not allow the energy transfer from ^3^(D_h_/A_g_)* to ^3^(ICT)* and ^3^(aggregate)*, leading to the decreased RISC rate and increased nonradiative IC process. It occurs in the case of R TADF emitter doped P(DPA-Si). In type (e), the T_1_ level of ^3^(D_h_/A_g_)* falls in between ^3^(ICT)* and ^3^(aggregate)*, which would lead to energy transfer from ^3^(D_h_/A_g_)* and ^3^(ICT)* to ^3^(aggregate)*. Hence, the rate of the nonradiative IC process would decrease. It occurs in the case of R TADF emitter doped P(DMAC-Ge) and P(DMAC-Si). In type (f), the T_1_ level of ^3^(D_h_/A_g_)* is higher than that of ^3^(ICT)* and ^3^(aggregate)*, which could result in energy transfer from the former to the latter, thus leading to a promotion of ^3^(ICT)*. The PLQY of this system is significantly enhanced since TPA-DCPP ^3^(ICT)* has a much higher PLQY than (aggregate)*. Besides, the radiative ^3^(ICT)* can increase the rate of RISC and thus enhance TADF emission. However, energy transfer from ^3^(ICT)* to ^3^(aggregate)* due to the lower energy of ^3^(aggregate)* can shorten the DF lifetime and increase the rate of the nonradiative IC process.

### 3.5 Influence of (D_h_/A_g_)* on the EQE of EL

**Figure 11:**
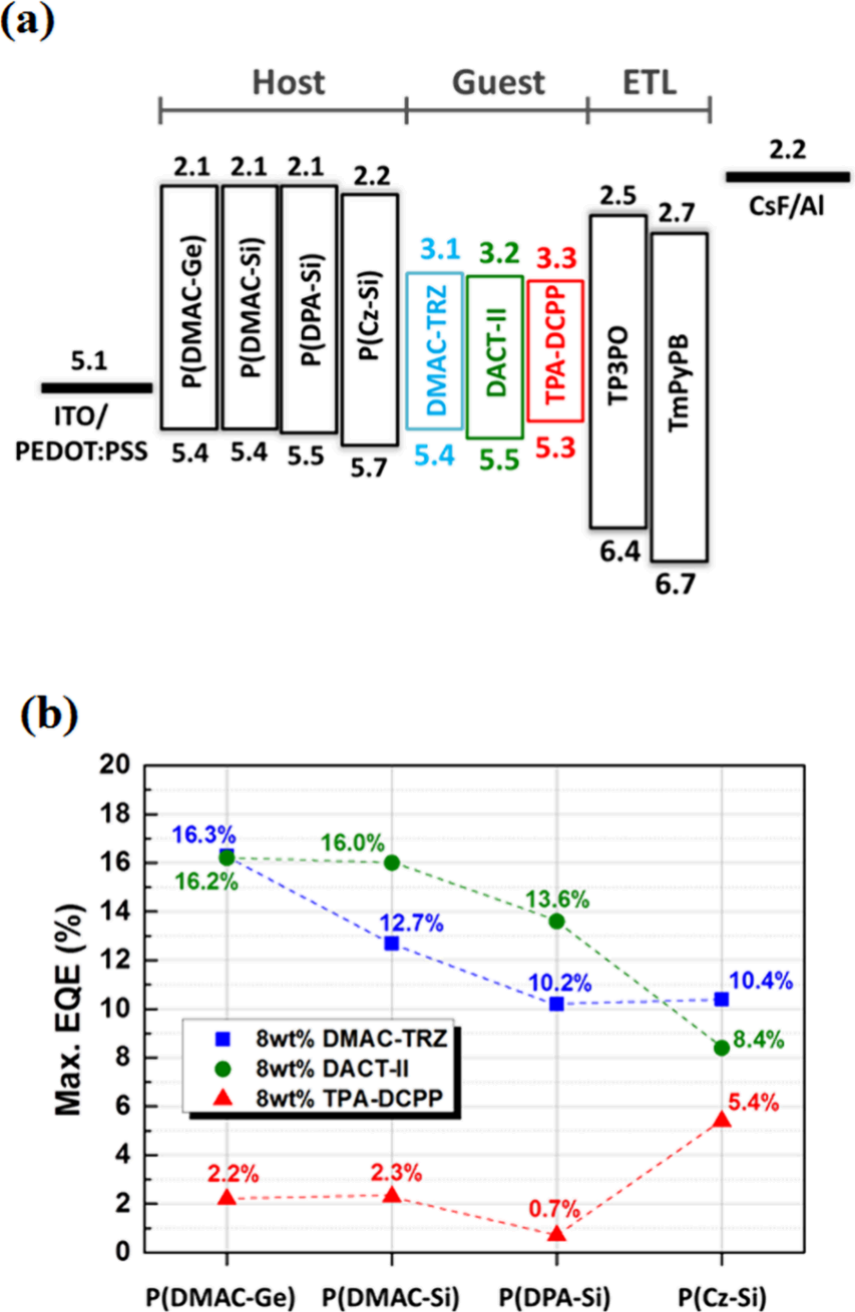
(a) Schematic diagram of the device structure and energy levels. (b) Maximum EQEs versus the different D-polymer hosts employed in the sB/G/R devices.

To understand if the host–guest interactions under photoexcitation also occur under electroexcitation, the EL measurements for the D-polymer host doped with sB/G/R TADFs as the EML are carried out and the device structure is ITO/PEDOT:PSS (30 nm)/ D-polymer host: 8 wt % guest (30 nm)/ TP3PO (3 nm)/ TmPyPB (52 nm)/ CsF (1 nm)/ Al (100 nm), where PEDOT:PSS, 1,3,5-tri(diphenylphosphoryl -phen-3-yl) benzene (TP3PO), 1,3,5-Tri[(3- pyridyl)-phen-3-yl] benzene (TmPyPB), and CsF act as hole injection, triplet exciton-blocking, electron transport, and electron injection layers, respectively (Figure 11a). Their current density–voltage–brightness (*I*–*V*–*B*), current efficiency (CE), and power efficiency (PE) profiles as well as EL spectra are shown in Figure S20, and their characteristic parameters are listed in Table S6. The maximum EQEs of the sB/G/R TADF devices using P(DMAC-Ge), P(DMAC-Si), P(DPA-Si), and P(Cz-Si) as hosts are as follows: for the DMAC-TRZ-based sky-blue devices, they are 16.3, 12.7, 10.2, and 10.4%; for the DACT-II based green devices, they are 16.2, 16.0, 13.6, and 8.4%; and for the TPA-DCPP-based devices, they are 2.2, 2.3, 0.7, and 5.4%, respectively (Figure 11b). These results highly correlate to their PLQY changes in the sB/G/R TADF doped D-polymer films (Figure 10a,b).

## 4 Conclusions

For the D-polymer host/TADF-guest system, we found the presence of exciplex (D_g_/A_g_)* through D/A interaction between two neighboring TADF molecules that could broaden its PL spectrum and decrease the PLQY of the TADF guest. The occurrence of (aggregate)* between two neighboring TADF molecules could induce a red shift and significantly decrease the PLQY of the TADF emitter due to the occurrence of the energy transfer process from (ICT)* to (aggregate)* and the low PLQY nature of (aggregate)*. The presence of (D_h_/A_g_)* through D/A interactions between D in the host and A in the TADF guest is also found, which could significantly influence the optical properties of the TADF emitter. For the formation of NR ^3^(D_h_/A_g_)* (Δ*E*_ST_ ≈ 0.5 eV), it is unfavorable for (ICT)* emission and thus weakens the emission of the TADF guest. However, the radiative ^3^(D_h_/A_g_)* (Δ*E*_ST_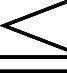 0.15 eV) provides a positive or negative effect on (ICT)* emission depending on its T_1_ level. As the T_1_ level of ^3^(D_h_/A_g_)* is higher than that of the TADF emitters, it can transfer energy to the latter and thus promote the triplet exciton population of the TADF emitter, which leads to a promoted RISC rate and thus enhances TADF emission. Conversely, if the T_1_ level of ^3^(D_h_/A_g_)* is lower than that of the guest, it would lead to ^3^(D_h_/A_g_)* self-emission, which cannot promote TADF emission. Furthermore, the PL efficiency of the TADF emitter influenced by ^3^(D_h_/A_g_)* also reflects on their EQEs in EL. These results imply that the formation of (D_h_/A_g_)* is unavoidable in the host/TADF-guest system. For (ICT)* emission, it is preferred to have radiative ^3^(D_h_/A_g_)* with a higher T_1_ level than (ICT)*, but it is undesirable to have NR ^3^(D_h_/A_g_)* and radiative ^3^(D_h_/A_g_)* with a lower T_1_ level than (ICT)*. Among the D-polymers studied, P(DMAC-Ge)/TRZ and P(Cz-Si)/DCPP give a higher T_1_ level of ^3^(D_h_/A_g_)*, which is beneficial for achieving high PLQY and RISC rates in the TADF emitter and thus shows higher EQEs in sB/G/R TADF EL devices. Our findings reveal the importance of (aggregate)* and (D_h_/A_g_)* on the optoelectronic properties of TADF emitters and provide useful guidelines on the selection of the donor moiety and polymer backbone structure for designing ideal small-molecule and polymer hosts as well as TADF emitters. These findings are also applicable to small-molecule host/TADF-guest systems and nondoped TADF systems.

## Supporting Information Available

Material synthesis and characterizations: ^1^H NMR and ^13^C NMR for all compounds; physical and optical characterizations: TGA, DSC, cyclic voltammogram, UV–vis absorption, PL, PLE, delayed emission, and lifetime decay of delayed emission; additional experimental details: optical properties, AFM images, PLQY data, and exciton lifetime calculation for the host–guest system; and PLED and OLED device fabrication and summary of device performance data (PDF)
