# Supplementary material for: Interacting Emission Species among Donor and Acceptor Moieties in a Donor-Grafted Polymer Host/TADF-Guest System and Their Effects on Photoluminescence and Electroluminescence
Source: ACS Appl Mater Interfaces. 2024 Oct 24;16(44):60715–31. doi: 10.1021/acsami.4c15933 (PMC11551908; doi:10.1021/acsami.4c15933)
Supplement: Supplementary file 1 — am4c15933_si_001.pdf [file am4c15933_si_001.pdf]

Supporting Information

Interacting Emission Species among Donor and Acceptor moieties in Donor-Grafted Polymer Host/TADF-Guest System and their Effects on Photoluminescence and Electroluminescence

*Yi-Hen Mao†, Miao-Ken Hung†, Shang-Ting Chung†, Sunil Sharma, Kuen-Wei Tsai, and Show-An Chen**

Department of Chemical Engineering, National Tsing-Hua University, Hsinchu 30013, Taiwan

*Email: sachen@che.nthu.edu.tw.

† These authors contributed equally.

Content

S1. General Measurements

A. Physical characterization ……………………………………………………………..S-3

B. Optical characterization …………………………………………………………...…..S-3

C. Electrochemical characterization ………………………………………………………..S-4

D. Calculation methodology ……………………………………………………………..S-4

E. Materials for device fabrications …………………..………………………………..…S-4

S2. Physical Characteristics

S2.1 Thermal, optical and electrochemical properties …………………………………….S-5

S2.2 Optical properties for the host-TADF guest system ………………………………….S-7

S2.3 Exciton lifetime calculation of the 8wt% TRZ doped and TADF emitters doped D-polymer host films ……………………………………………………………………S-17

S2.4 Exclusion of energy level and dipole effects on the PLQY of TADF emitter ………..S-20

S3. Device Performances ……………………………………………………….S-22

S4. ^{1}HNMR spectra and ^{1}CNMR spectrum …………………………………….S-26

S1. General Measurements

A. Physical characterization: The ^{1}H NMR and ^{13}C NMR spectra were recorded by using VARIAN UNITYINOVA 500 NMR. Mass spectra were measured in fast atom bombardment (FAB) mode by High Resolution Mass Spectrometer (JEOL JMS-700). Gel permeation chromatography (from Waters) assembled with a UV detector and three columns in series (Styragel HR2~4 from Waters) was used to measure molecular weight distributions relative to polystyrene standards at 40 ^{o}C. Differential scanning calorimetry (DSC) analyses were measured by a Perkin-Elmer Diamond DSC Difference Scanning Calorimeter at a heating rate of 15 ^{o}C min^{-1} from 70 to 350 ^{o}C under nitrogen atmosphere. The thickness of solid film is measured by a Tencor P-10 Surface Profiler.

B. Optical characterization: Ultraviolet-visible (UV-Vis) spectra and photoluminescence (PL) and electroluminescence (EL) spectra were measured using an UV-Vis spectrometer (Perkin-Elmer, Lambda 35) and a fluorescence spectrometer (Jobin Yvon Horiba, Fluoromax-3), respectively. The absolute PL quantum yields (PLQYs) of the doped films were determined by the fluorescence spectrometer (Jobin Yvon Horiba, Fluoromax-3) adapted with an integrating sphere under N_2 atmosphere. Transient PL measurement of the doped films were obtained using a 350 nm pulsed Ti:sapphire femtosecond laser (Spectra-Physics Hurricane) as the excitation source and the PL decay curves were measured using a 1024-channel time-correlated single photon counting (TCSPC) system with a microchannel plate photomultiplier tube (Hamamatsu Photonics R3809U-50) and a spectrometer (Edinburgh, Lifespec-ps with TCC900 data acquisition card). The phosphorescence spectra were measured by using a 300 nm pulsed Ti:sapphire laser (Spectra-Physics Hurricane) as the excitation source, and recorded by a monochromator (Princeton Instruments Acton SpectraPro 2300i) and then to a gated intensified CCD camera (Princeton Instruments PIMAX), and the measurements were carried out in a cryostat at 77 K with 1ms delay time and under a dynamic vacuum of 10^{-3} Torr, and the triplet energy (T_1) levels were determined from the onset values of the phosphorescence spectra in

solid film state.

C. Electrochemical characterization: The energy levels were measured by using CH Instruments 611E electrochemical analyzer. All measurements were carried out at room temperature with a conventional three-electrode configuration consisting of the D-polymer film coated on platinum (Pt) plate as working electrode, Pt plate as counter electrode, and a nan-aqueous $Ag/AgNO_3$ (0.01 M in acetonitrile) as reference electrode. In all the experiments, 0.1M *n*-Bu_4NPF_6 in acetonitrile used as the electrolyte and ferrocene/ferrocenium (Fc/Fc^+) couple served as external standard use, and the cyclic voltammogram (CV) were observed at a scan rate of 100 mV/s. The ionization potential (E_{HOMO}) of the D-polymer was determined from the onset oxidation potential relative to that of Fc^+/Fc plus 4.8 eV (the Fc^+/Fc energy level below the vacuum level), which means that E_{HOMO} can be calculated using the equation E_{HOMO} [eV] = E_{onset} + 4.8 eV. The energy level of LUMO was deduced from the onset of UV-vis spectrum (E_g) and that of HOMO, which means that E_{LUMO} [eV] = $E_{HOMO} + E_g$.

D. Calculation methodology: The excited-state dipole moments were calculated by the time-dependent DFT (TD-DFT) using the Gaussian 09 package at the B3LYP/6-13G(d,p) level.Spectral deconvolution was performed using nonlinear curve fit with Gaussian function by the OriginPro 9.0 software.

E. Materials for device fabrications:

The sky-blue and green TADF emitters, 9,9-Dimethyl-9,10-dihydroacridine-2,4,6- triphenyl-1,3,5-triazine (DMAC-TRZ) and 9-[4-(4,6-Diphenyl-1,3,5-triazin-2-yl)phenyl] -N3,N3,N6,N6-tetraphenyl-9H-Carbazole-3,6- diamine (DACT-II) and the electron transport material 1,3,5-Tri(m-pyridin-3-ylphenyl)benzene (TmPyPB) were purchased from Shine Materials Technology Co.,Ltd, Taiwan and used without further purification. The exciton blocking and electron transport materials 1,3,5-Tri(diphenylphosphoryl-phen-3-yl) benzene (TP3PO) and 5-terphenyl-1,3- phenylene-bis(diphenylphosphine oxide) (POPH) and the red TADF emitter 7,10-Bis(4-(diphenylamino)phenyl)-2,3-dicyanopyrazino phenanthrene (TPA-

DCPP) were purchased from Luminescence Technology Corp., Taiwan and used without further purification.

S2. Physical Characteristics

S2.1 Thermal, optical and electrochemical properties

Table S1. Physical properties of the D-polymer hosts

D-polymer host	$T_{d, 5\%}$ (˚C) [a]	Tg (˚C)[b]	$\lambda_{abs, peak}$ (nm)[c]	$\lambda_{PL, peak}$ (nm)[d]	E_T (eV)[e]	E_g (eV)[f]	HOMO (eV)[g]	LUMO (eV)[h]
**P(DMAC-Ge)**	434.4	259	280	395	2.86	3.31	-5.39	-2.08
**P(DMAC-Si)**	432.7	258	278	405	2.78	3.31	-5.40	-2.09
**P(DPA-Si)**	406.2	259	300	396	2.78	3.32	-5.45	-2.13
**P(Cz-Si)**	498	261	253, 293	402	2.76	3.52	-5.72	-2.20

[a] Thermal decomposition temperature, 5% weight loss of polymers obtained from TGA measurement. [b]Obtained from DSC measurement. [c&d]Measured in film state. [e]Calculated from the onset value of phosphorescence spectrum at 77K with 1mS delay time. [f]Estimated from the onset value of absorption spectrum. [g] Determined from the onset value of oxidation potential shown in Figure S4. [h]Estimated from the equation: LUMO = HOMO + E_g.

Thermal properties:

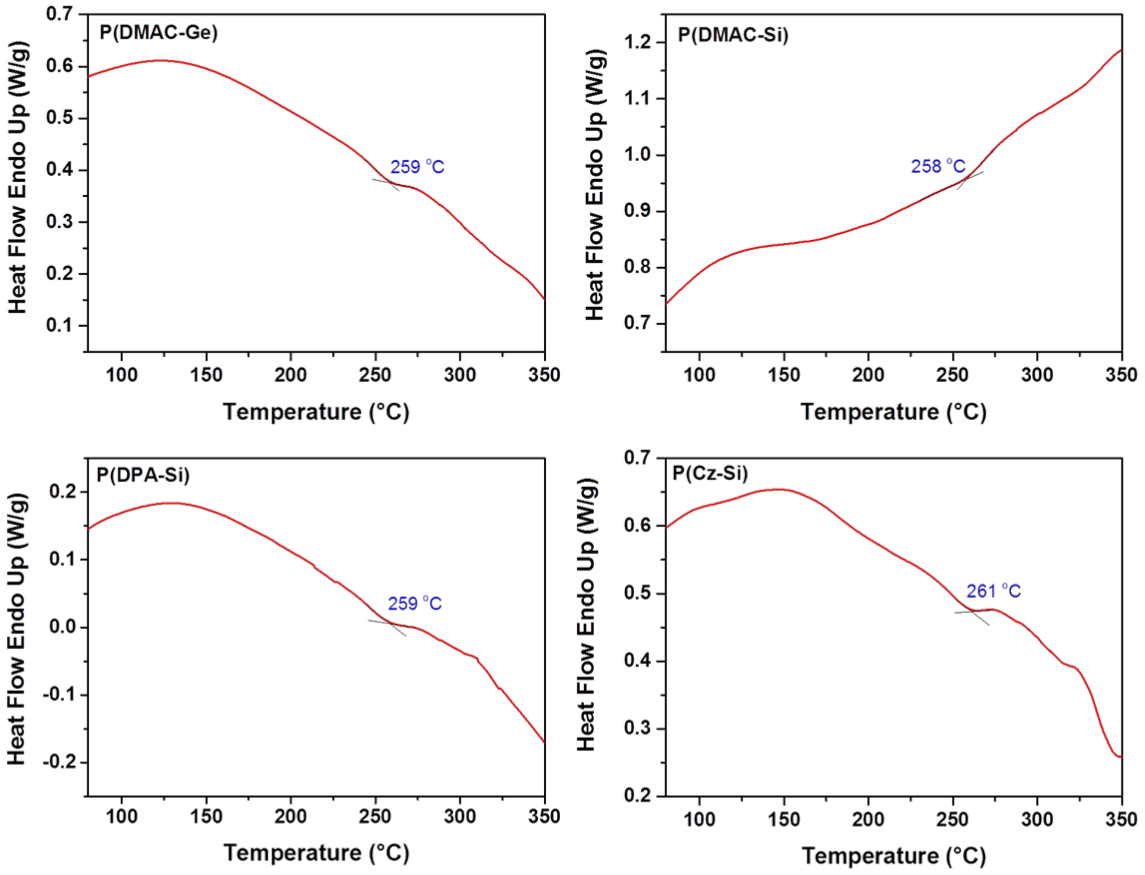

Figure S1. DSC traces of the D-polymer hosts at a heating rate of 15˚C/min.

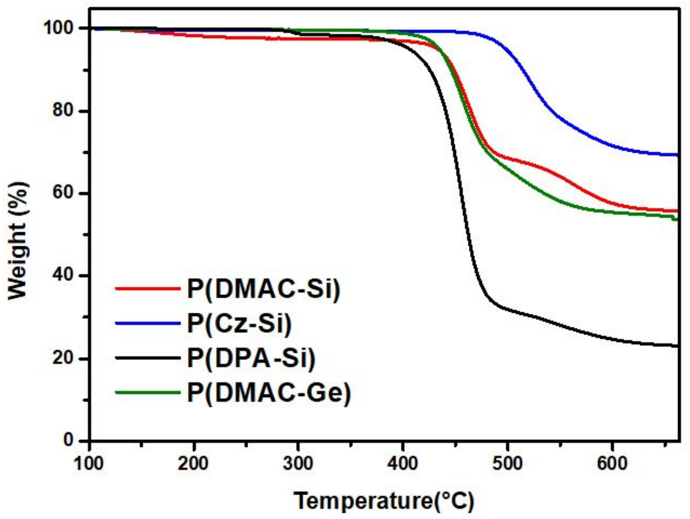

Figure S2. TGA curve of the D-polymer hosts at a heating rate of 10˚C/min.

Photophysical properties:

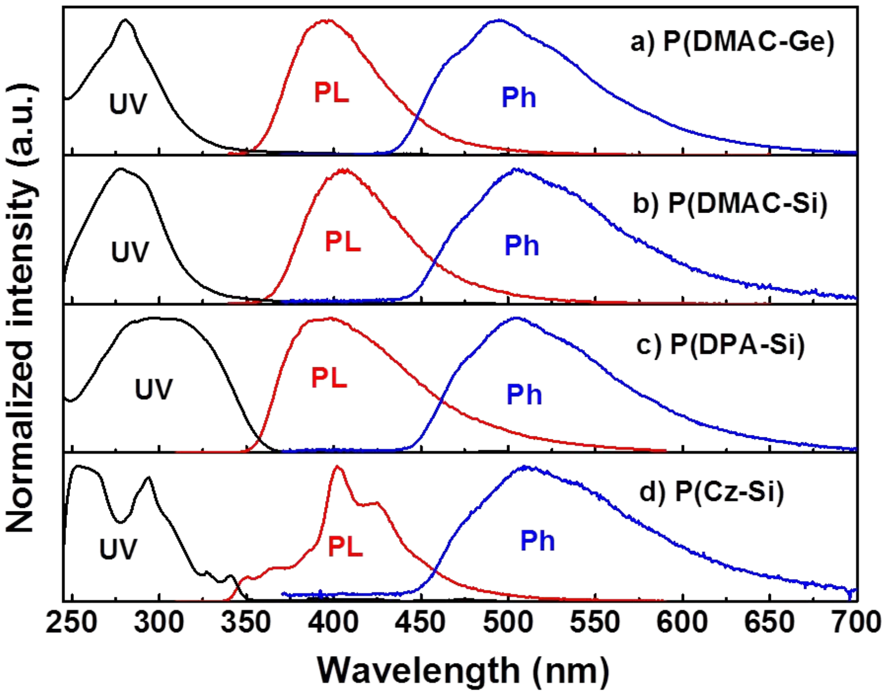

Figure S3. UV-vis absorption (UV), photoluminescence (PL) and phosphorescence (Ph) spectra for the D-polymer hosts: (a) P(DMAC-Ge), (b) P(DMAC-Si), (c) P(DPA-Si) and (d) P(Cz-Si) in solid film.

Electrochemical properties:

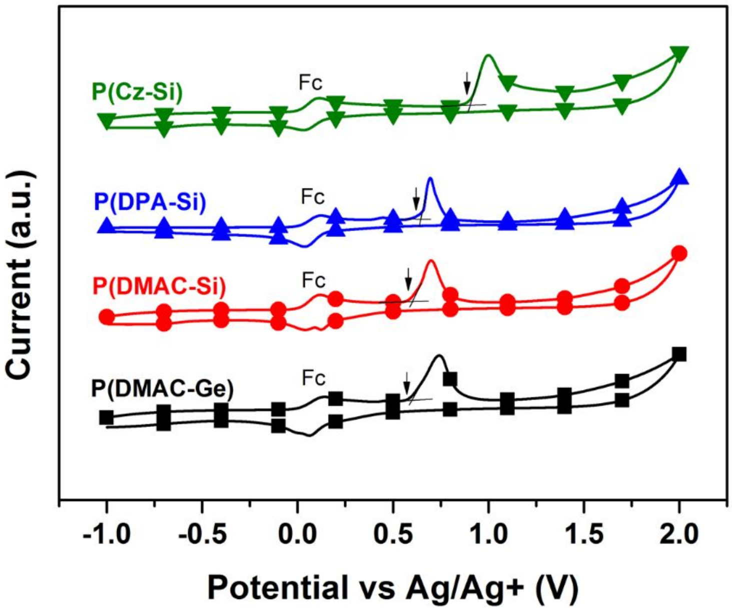

Figure S4. Cyclic voltammograms of oxidative curves for the D-polymer hosts.
*

S2.2 Optical properties for the host-TADF guest system

Table S2. PL parameters of the 8wt% sB/G/R TADF guest doped D-polymer films.

Emitter	Polymer	$\lambda_{PL, max}$ [nm]	FWHM [nm]
DMAC-TRZ	P(DMAC-Ge)	481	77
	P(DMAC-Si)	479	79
	P(DPA-Si)	483	83
	P(Cz-Si)	482	84
DACT-II	P(DMAC-Ge)	515	96
	P(DMAC-Si)	512	95
	P(DPA-Si)	514	94
	P(Cz-Si)	521	98
TPA-DCPP	P(DMAC-Ge)	646	130
	P(DMAC-Si)	644	133
	P(DPA-Si)	677	153
	P(Cz-Si)	609	115

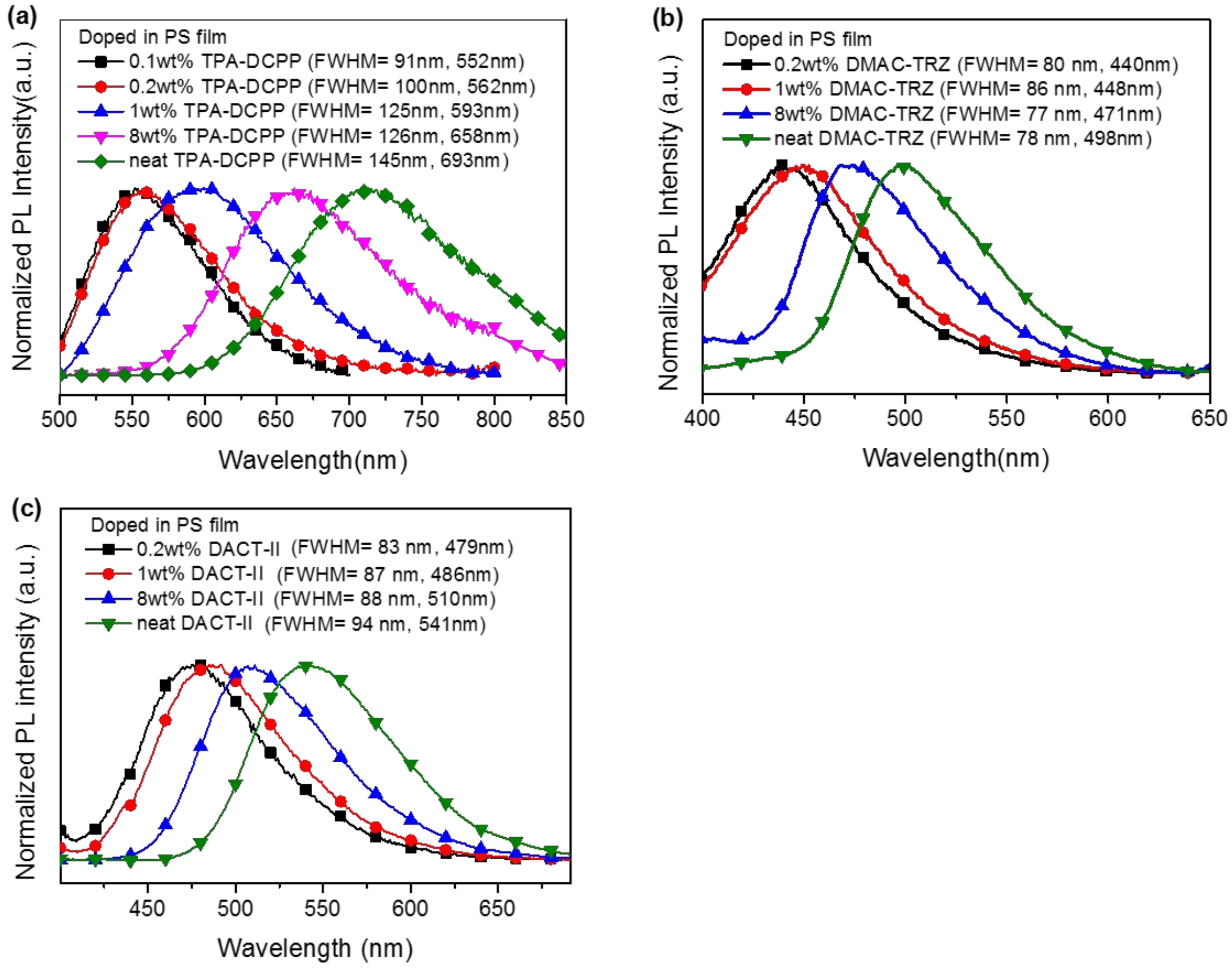

Figure S5. PL spectra, corresponding FWHMs and peak wavelengths of the doped PS films for (a) TPA-DCPP, (b) DMAC-TRZ and (c) DACT-II at various doping concentrations.

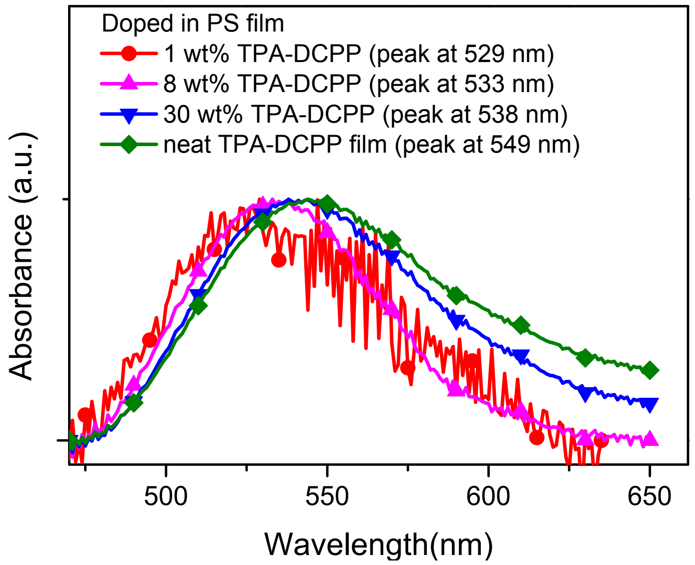

Figure S6. The aggregates UV spectra that are obtained from each UV spectrum of 1~100wt% TPA-DCPP doped PS film after the subtraction by that of the 0.1wt% one according to the UV

spectra shown in Figure 1a.

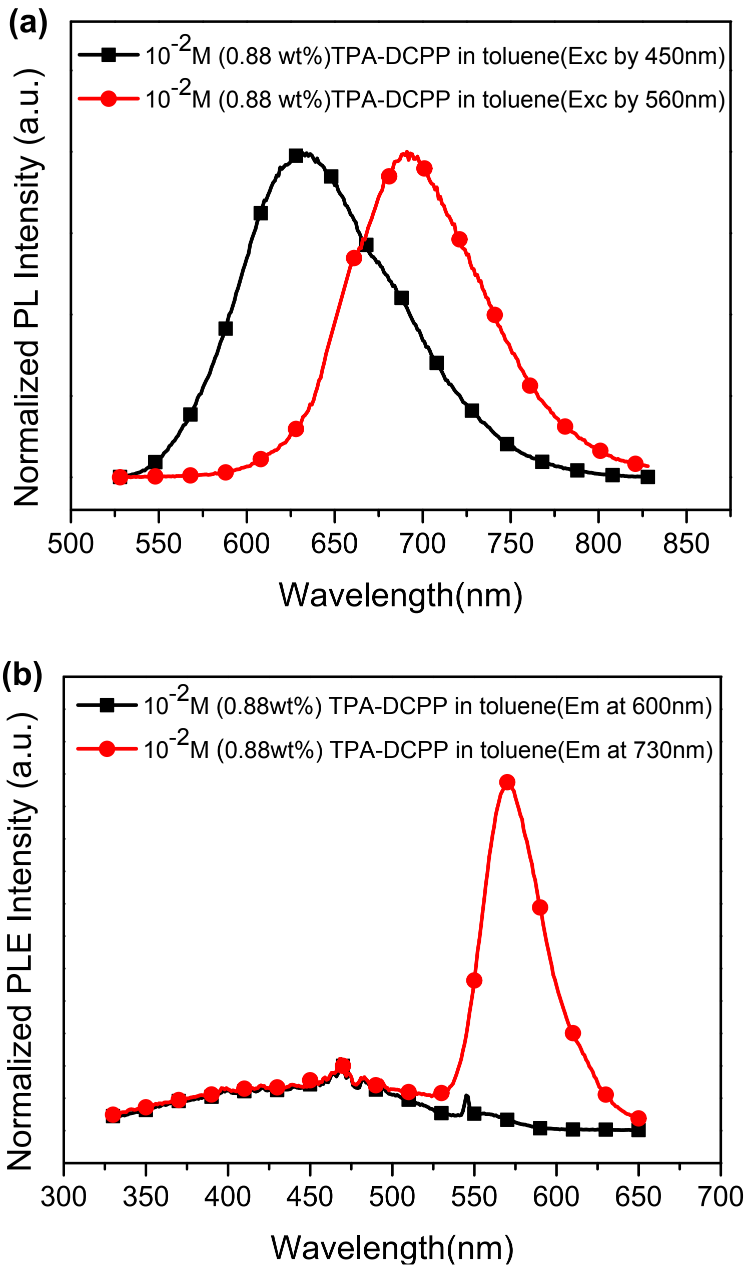

Figure S7. (a) PL (Excited by 450 and 560 nm) spectra normalized at the peaks of 10^{-2} M TPA-DCPP in toluene and (b) PLE (Emission at 600 and 730 nm) spectra normalized at 468 nm

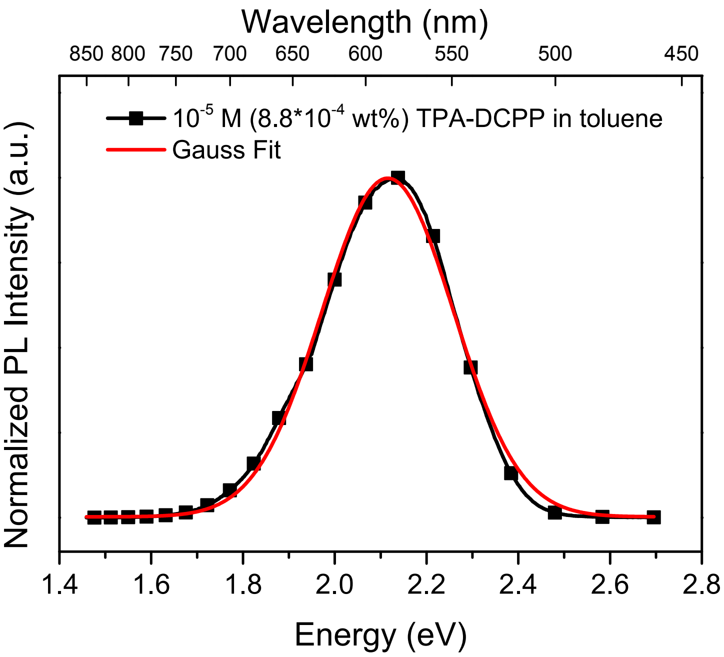

Figure S8. PL spectra of TPA-DCPP in diluted solution (1×10^{-5} M, $8.8*10^{-4}$ wt% in toluene) and their Gaussian fit plot.

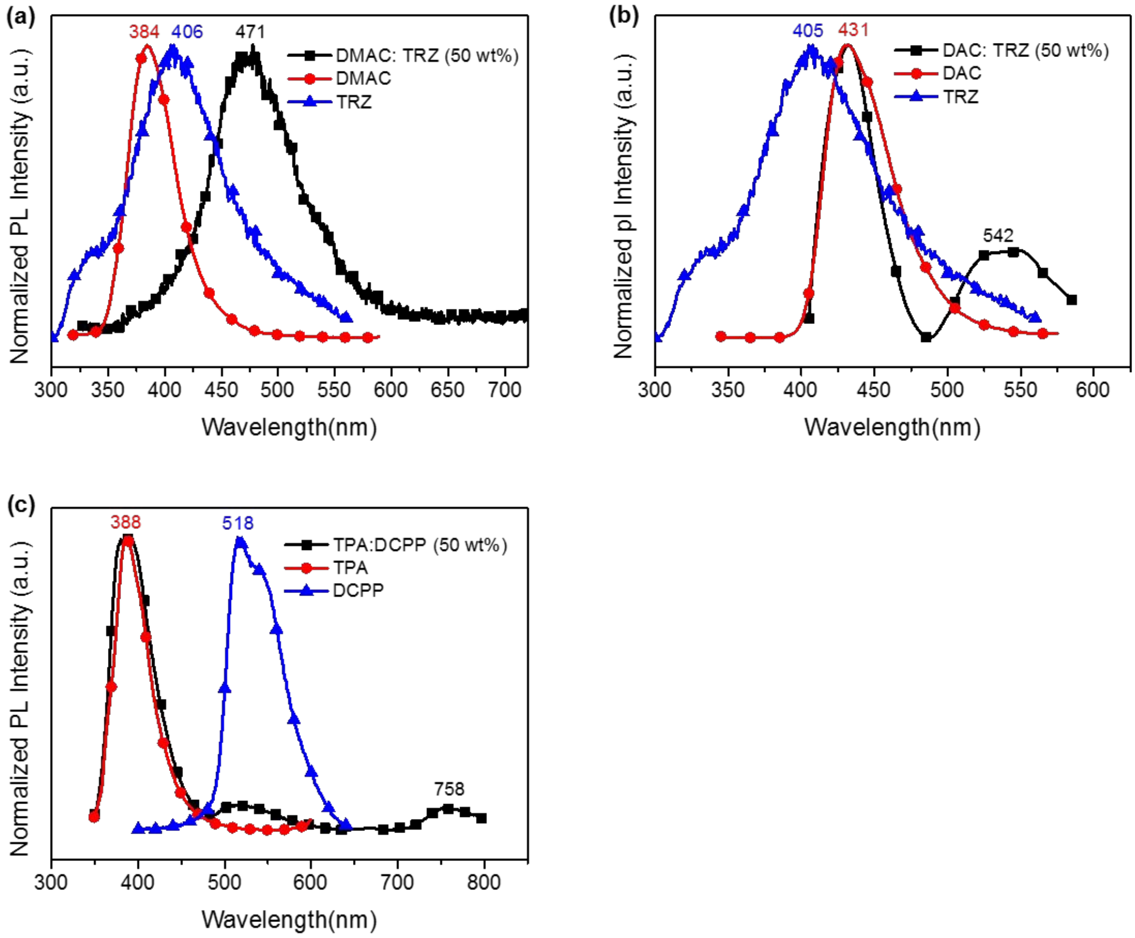

Figure S9. PL spectrum of the solid films (a)DMAC:TRZ, DMAC and TRZ; (b)DAC:TRZ, DAC and TRZ; (c)TPA:DCPP, TPA and DCPP.

Table S3. FWHM, emission peak wavelength and component fraction of 0.1~100 wt% TPA-DCPP doped PS film

Compounds	$\lambda_{PL,\,max}$ [nm]	FWHM [nm]	$\lambda_{PL1,\,max}$ [nm]	A_1%	FWHM,1 [nm]	$\lambda_{PL2,\,max}$ [nm]	A_2%	FWHM,2 [nm]	$\lambda_{PL3,\,max}$ [nm]	A_3%	FWHM,3 [nm]
**PS+0.1%TPA-DCPP**	552	91	558	100	-	-	-	-	-	-	-
**PS+1%TPA-DCPP**	593	125	582	64	97	656	36	152	-	-	-
**PS+5%TPA-DCPP**	621	125	607	34	66	660	63	91	738	3	65
**PS+8%TPA-DCPP**	658	126	-	-	-	660	84	107	761	16	117
**PS+30%TPA-DCPP**	664	131	-	-	-	663	83	110	775	17	138
**PS+50%TPA-DCPP**	672	135	-	-	-	670	82	99	780	18	123
**TPA-DCPP**	693	145	-	-	-	697	77	98	785	23	85

The subscripts are referred to: 1. ICT emission 2. Aggregate emission 3. $(D_g/A_g)^*$ emission.

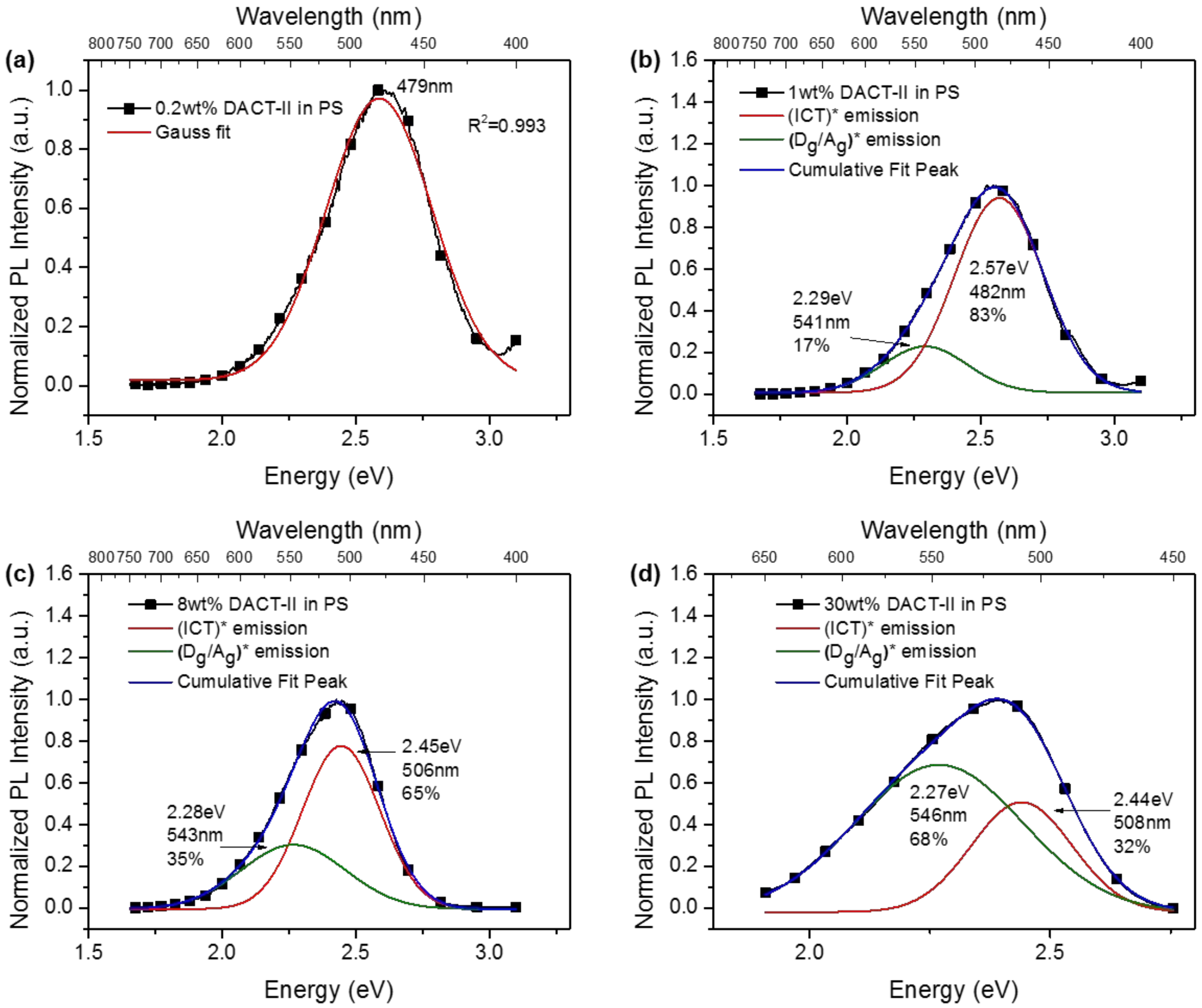

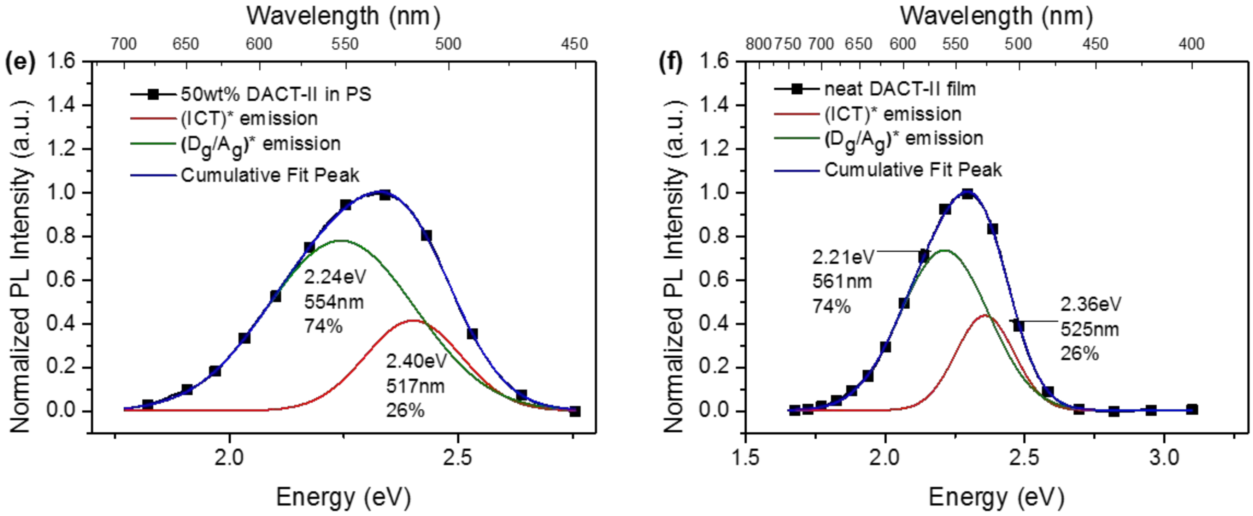

Figure S10. Deconvoluted plots and fractions of emitting species from the PL spectra of (a) 0.2wt% (b) 1wt% (c) 8wt% (d) 30wt% (e) 50wt% DACT-II doped PS film and (f) neat DACT-II film

Table S4. FWHM, emission peak wavelength and component fraction of 0.2~100 wt% DACT-II doped PS film

Compounds	$\lambda_{PL, max}$ [nm]	FWHM [nm]	$\lambda_{PL1, max}$ [nm]	A_1%	$FWHM_1$ [nm]	$\lambda_{PL2, max}$ [nm]	A_2 %	$FWHM_2$ [nm]
PS+0.2%DACT-II	479	83	479	100		-	-	
PS+1% DACT-II	486	87	482	83	73	541	17	87
PS+8% DACT-II	510	88	506	65	70	543	35	106
PS+30% DACT-II	517	93	508	32	50	546	68	94
PS+50% DACT-II	533	94	517	26	55	554	74	92
Neat DACT-II	541	94	525	26	54	561	74	92

The subscripts are referred to: 1. ICT emission 2. $(D_g/A_g)^*$ emission.

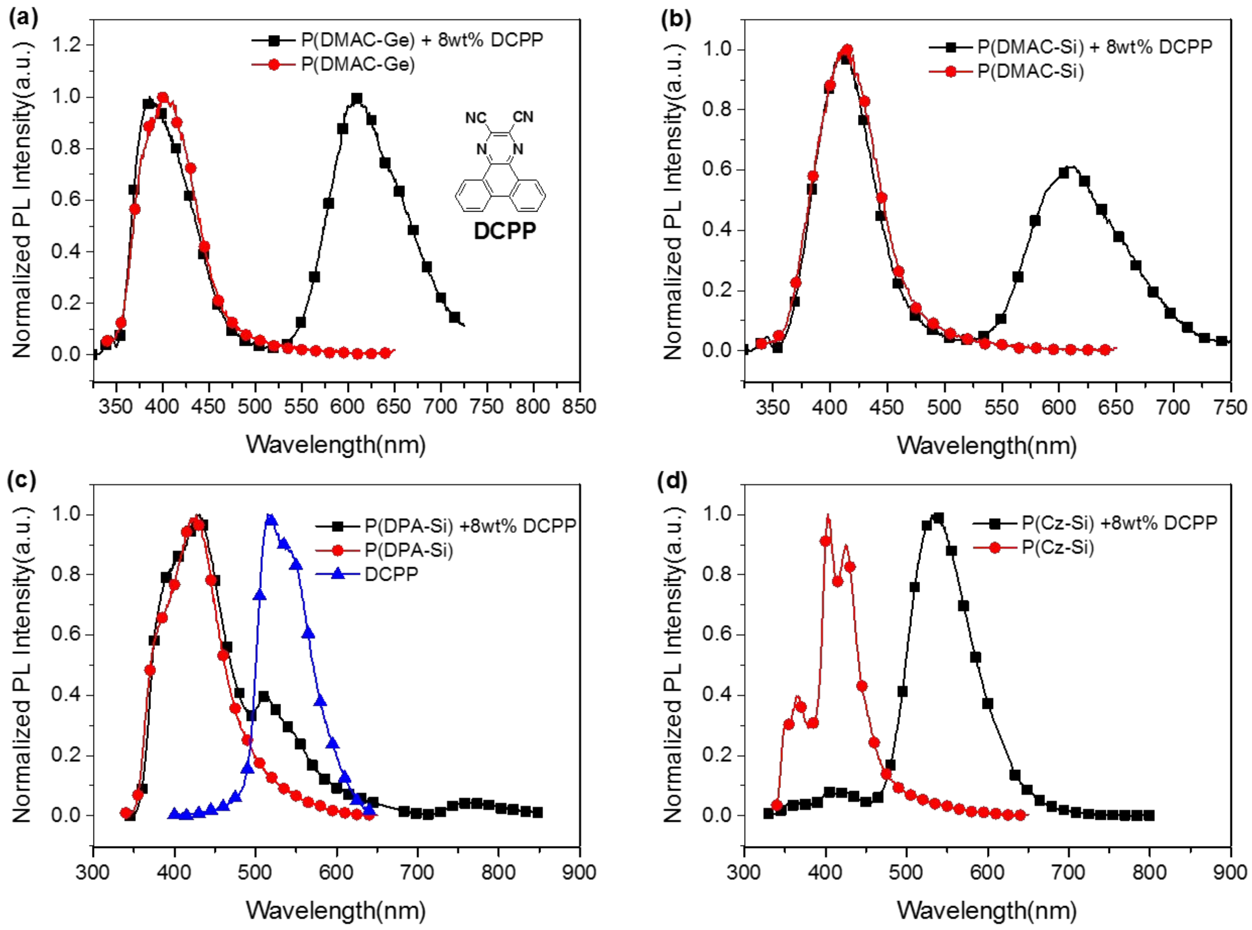

Figure S11. PL spectra of the neat D-polymer films and 8wt% DCPP doped in (a) P(DMAC-Ge), (b) P(DMAC-Si), (c) P(DPA-Si), and (d) P(Cz-Si) films

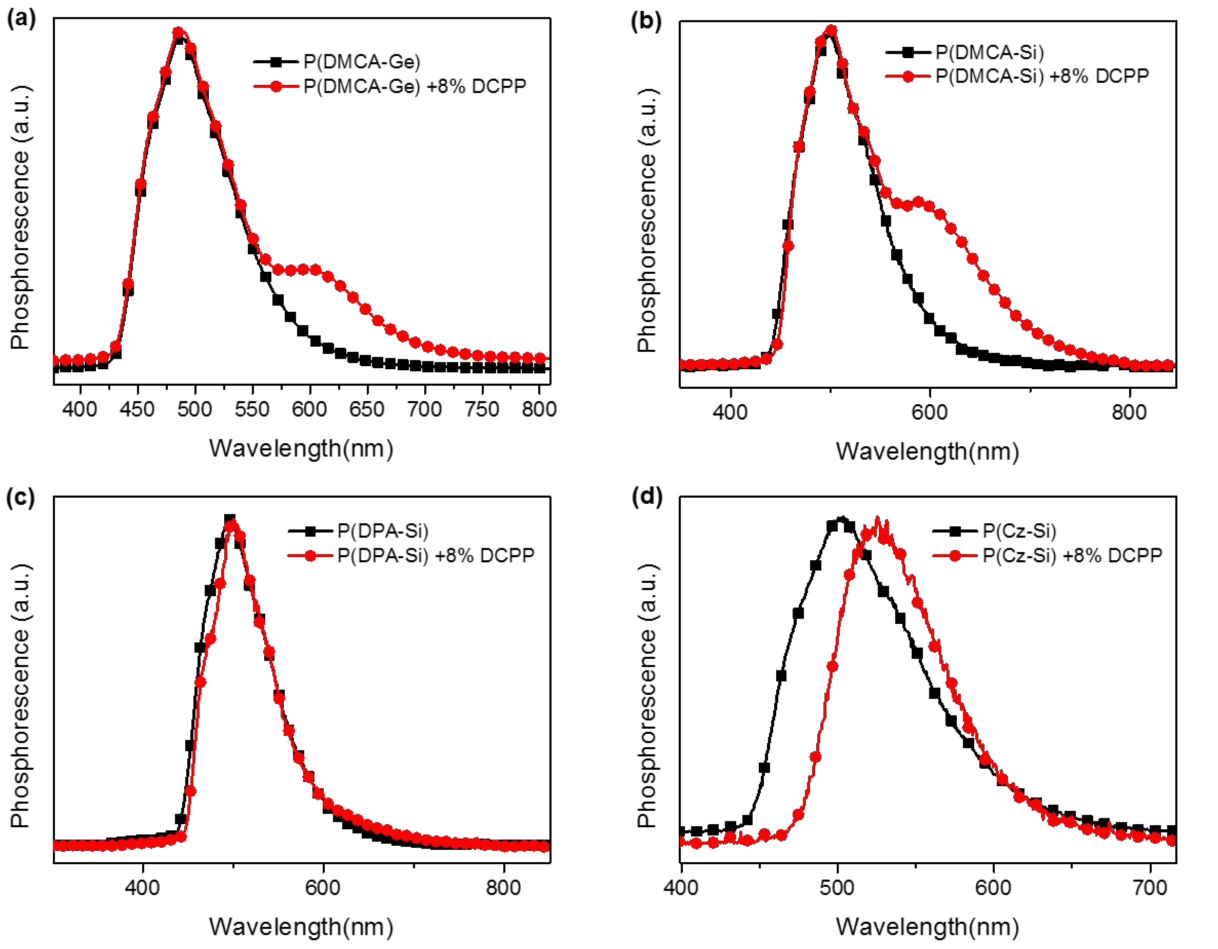

Figure S12. Phosphorescence spectra at 77K with 1ms delay time for 8wt% DCPP doped in the (a) P(DMAC-Ge), (b) P(DMAC-Si), (c) P(DPA-Si) and (d) P(Cz-Si) films

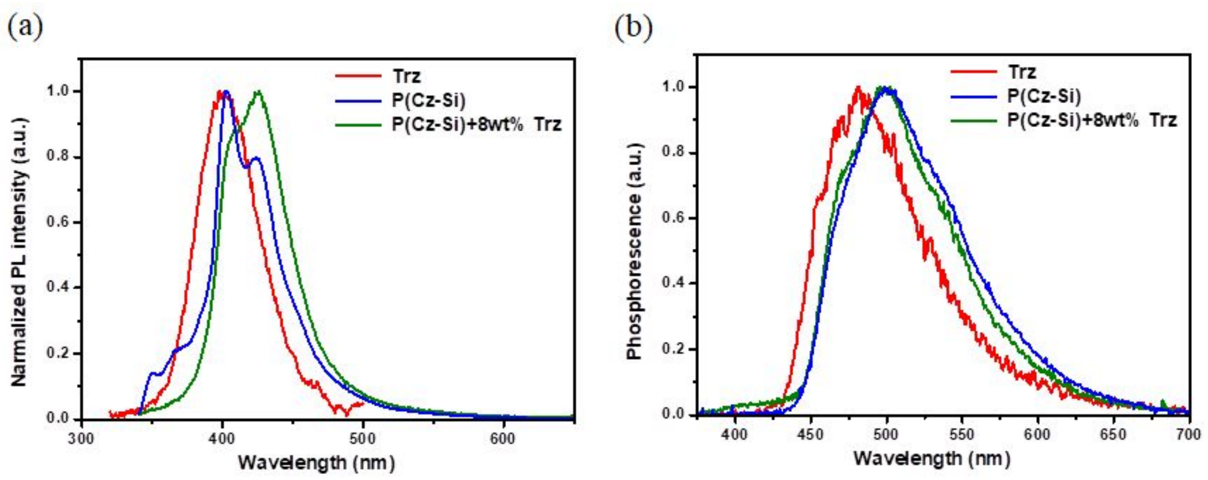

Figure S13. (a) PL spectra of Trz, P(Cz-Si) and 8wt% TRZ doped in the P(Cz-Si) host films at RT. (b) Phosphorescence spectra at 77K with 1ms delay time for 8wt% DCPP doped in the P(Cz-Si) films

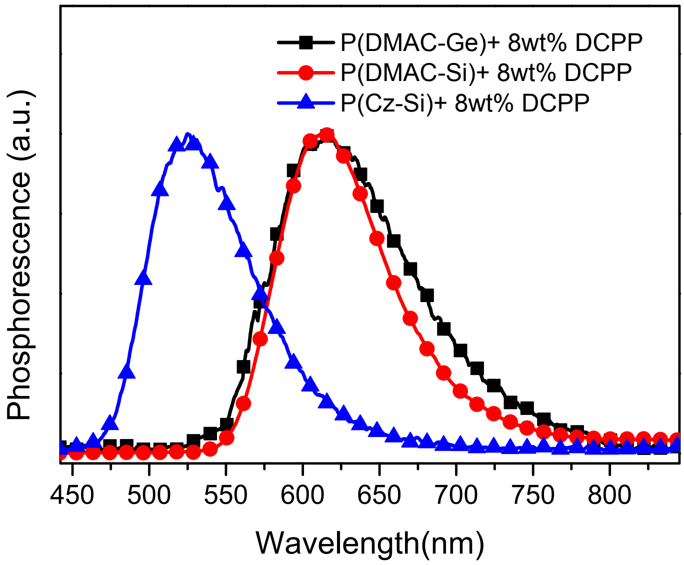

Figure S14. Phosphorescence spectra at 77K with 1 ms delay time for 8wt% DCPP doped D-polymer host films

Table S5. Deconvoluted PL emission spectral of D-polymers doped with 8 wt% TPA-DCPP films

Emitter	D-polymer	$\lambda_{PL,\ max}$ [nm]	$\lambda_{PL1,\ max}$ [nm]	A_1 %	$FWHM_1$ [nm]	$\lambda_{PL1,\ max}$ [nm]	A_2 %	$FWHM_2$ [nm]	$\lambda_{PL1,\ max}$ [nm]	A_3 %	$FWHM_3$ [nm]
TPA-DCPP	P(DMAC-Ge)	646	610	13	64	660	79	114	761	8	83
	P(DMAC-Si)	644	607	17	72	660	75	111	761	8	85
	P(DPA-Si)	677	-	-	-	670	84	126	761	16	102
	P(Cz-Si)	609	599	64	87	663	33	107	761	3	100

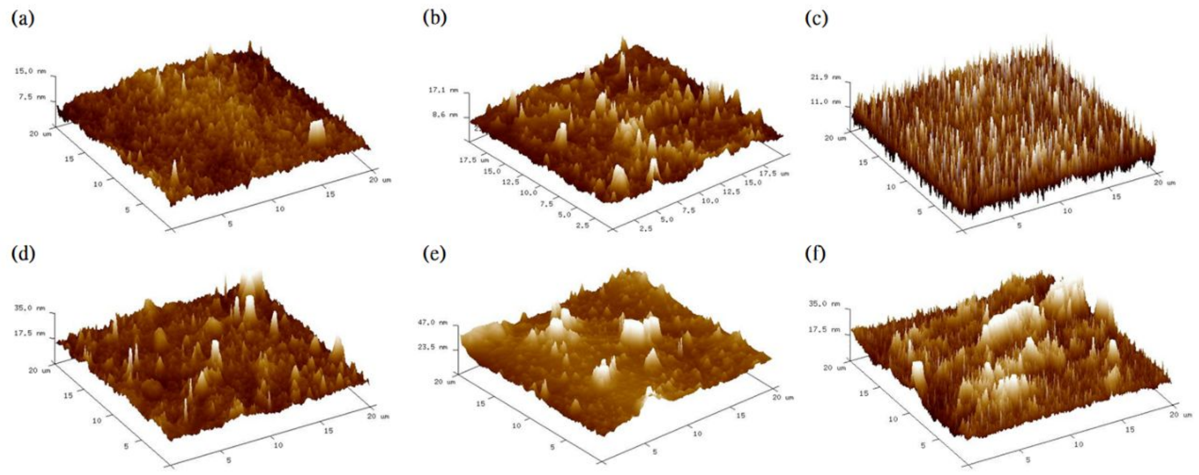

Figure S15. AFM 3D image of TPA-DCPP doped polystyrene films with different dopant concentration (a) 0.1 wt % (b) 1 wt % (c) 5wt % (d) 8 wt % (e) 30 wt % (f) 50 wt %.

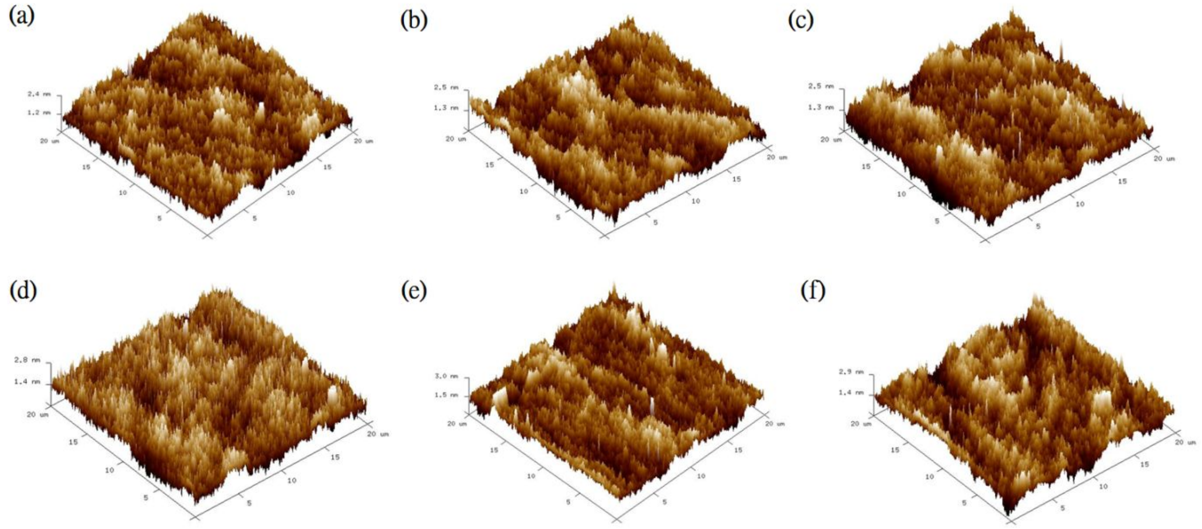

Figure S16. AFM 3D image of DACT-II doped polystyrene films with different dopant concentration (a) 0.1 wt % (b) 1 wt % (c) 5wt % (d) 8 wt % (e) 30 wt % (f) 50 wt %.

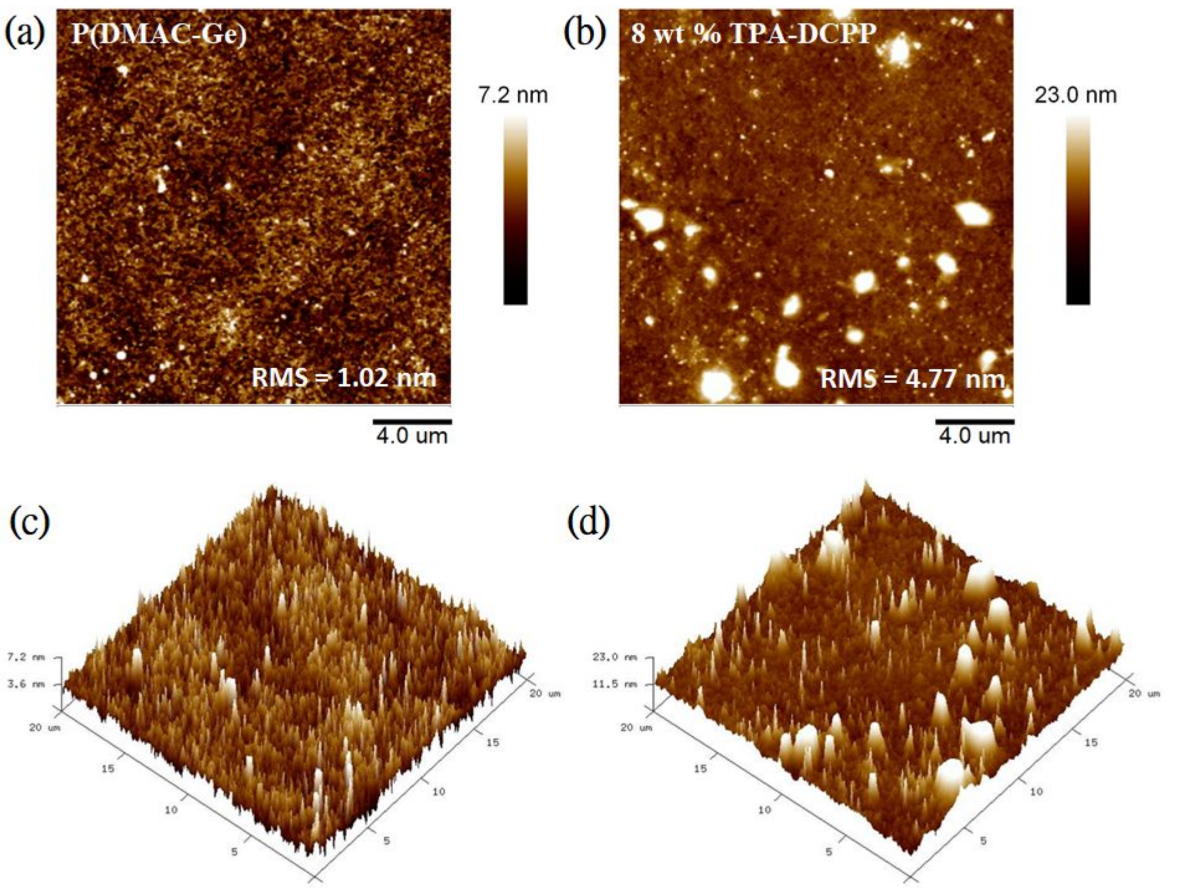

Figure S17. AFM image of (a) P(DMAC-Ge) polymer thin film and (b) 8 wt % TPA-DCPP doped P(DMAC-Ge) films. 3D AFM images of (c) P(DMAC-Ge) polymer thin film and (d) 8 wt % TPA-DCPP doped P(DMAC-Ge) films.

S2.3 Exciton lifetime calculation of the 8wt% TRZ doped D-polymer host films

Table 1 shows the fitting parameters of exciton lifetimes (τ_1 and τ_2) and corresponding fractions (A_1% and A_2%) for the D-polymer host doped with 8wt% TRZ films. These parameters were obtained by two exponentials fitting of the decay curves (Figure 2a) with the following equation (Eq1):

$$I_{PL}(\mathrm{t}) = A_1 e^{-t/\tau_1} + A_2 e^{-t/\tau_2} \quad (1)$$

Exciton lifetime and emission component determinations:

Tables 3 show the fitting parameters of exciton lifetimes (τ_1, τ_2 and τ_3) and corresponding fractions (A_1%, A_2% and A_3%) for the polymer films doped with 8wt% DMAC-TRZ, DACT-II and TPA-DCPP, respectively. All of the parameters were obtained by two or three

exponentials fitting of the decay curves (Figures S13) with the following equation (Eq2):

$$\text{Three exponentials fitting: } I_{PL}(\mathrm{t}) = A_1 e^{-t/\tau_1} + A_2 e^{-t/\tau_2} + A_3 e^{-t/\tau_3} \quad (2)$$

Rate constant determinations:

To evaluate the average prompt lifetime τ_{av}, we adopted the following equation[S7](Eq3):

$$\tau_{av} = \sum A_i \tau_i \quad (3)$$

where A_i and τ_i are the exciton lifetimes τ_1 and τ_2 and their fractions of A_1 and A_2, respectively, and A_3% and τ_3 are not included.

Thus, the prompt and delayed exciton lifetimes, τ_p and τ_d were determined from τ_{av} and $\tau_{1,}$ respectively.[S8] The parameters of exciton lifetimes (τ_p and τ_d) and corresponding fractions (τ_p% and τ_d%) for the D-polymer host films doped with 8wt% DMAC-TRZ, DACT-II and TPA-DCPP (estimated from Table 3), respectively, are summarized in Tables S6.

Based on the parameters of Table S6, the rate constants for the host doped with 8wt% DMAC-TRZ films were determined by using the reported method[S9] shown as follows(Eq4~9):

$$k_p = \phi_p / \tau_p \quad (4)$$

$$\phi_p + \phi_d = k_p / (k_p + k_{IC}) \quad (5)$$

$$\phi_p = k_p / (k_p + k_{IC} + k_{ISC}) \quad (6)$$

$$\phi_{ISC} = k_{ISC} / (k_p + k_{IC} + k_{ISC}) \quad (7)$$

$$k_d = \phi_d / (\phi_{ISC} \tau_d) \quad (8)$$

$$k_{RISC} = \left(\frac{k_p k_d}{k_{ISC}}\right)\left(\frac{\phi_d}{\phi_p}\right) \quad (9)$$

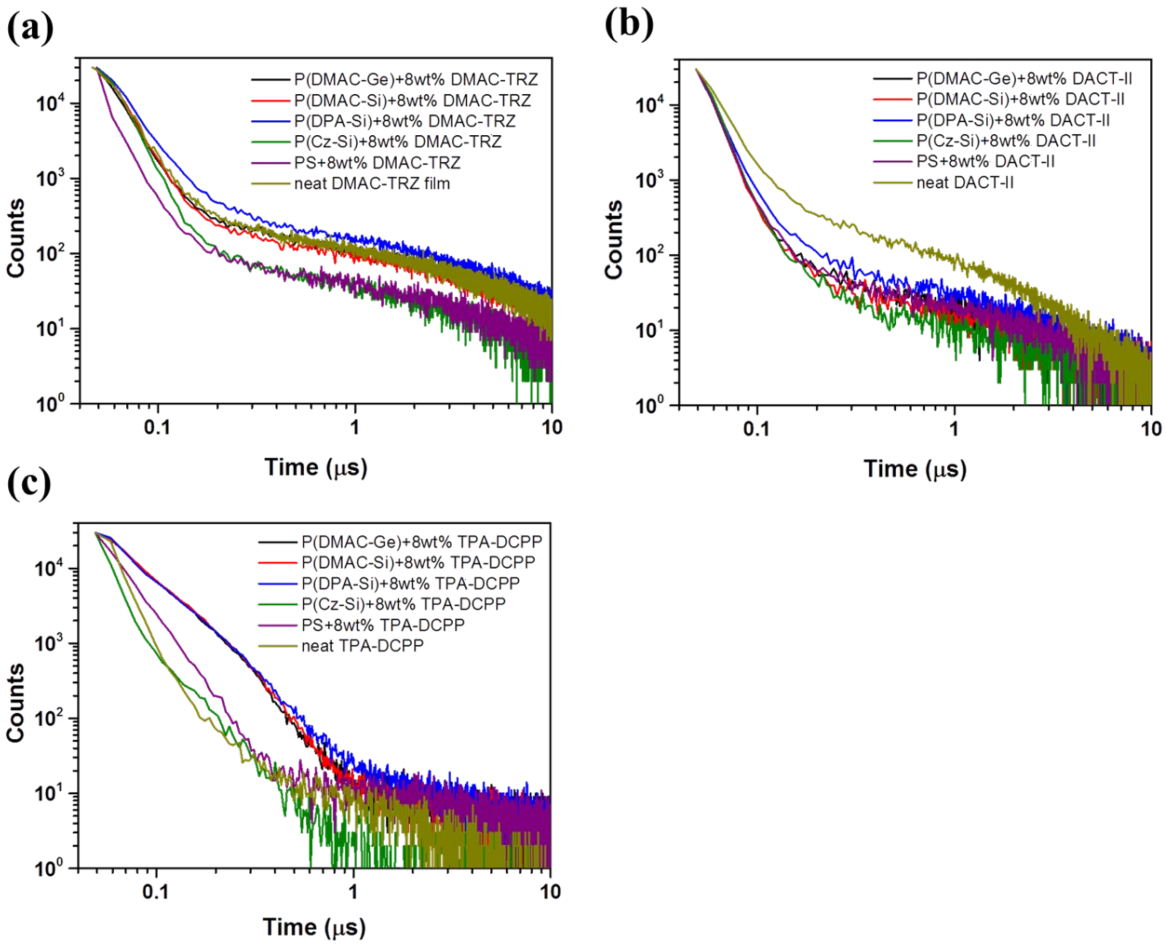

Figure S18. Transient PL decays of the polymer doped with 8wt% sB/G/R TADF films and the TADF emitter neat films: (a) DMAC-TRZ, (b) DACT-II and (c) TPA-DCPP as emitters. The measurement was carried out under excitation by a *fs* pulse laser (350nm) at RT, and these decay curves were monitored at their maximum PL emissions.

Table S6. Prompt and delayed exciton lifetimes of the D-polymer doped with 8wt% DMAC-TRZ, DACT-II and TPA-DCPP f films, respectively.

Emitter	D-polymer	τ_p [ns]	τ_p %	τ_d [μs]	τ_d %	χ^2
DMAC-TRZ (Sky-blue)	P(DMAC-Ge)	18.5	60.0	2.6	40.0	1.16
	P(DMAC-Si)	16.8	66.7	2.5	33.3	1.13
	P(DPA-Si)	20.1	59.1	2.6	40.9	1.34
	P(Cz-Si)	15.6	83.6	2.2	16.4	1.21
DACT-II (Green)	P(DMAC-Ge)	12.1	86.3	1.7	13.7	1.30
	P(DMAC-Si)	11.8	89.1	1.7	10.9	1.17
	P(DPA-Si)	14.8	83.3	1.8	16.7	1.28
	P(Cz-Si)	12.2	93.4	1.5	6.6	1.04
TPA-DCPP (Red)	P(DMAC-Ge)	56.8	97.6	1.2	2.4	1.40
	P(DMAC-Si)	59.4	97.7	1.3	2.3	1.29

P(DPA-Si)	57.6	95.8	1.2	4.2	1.56
P(Cz-Si)	22.7	95.5	0.2	4.5	0.68

Table S7.PLQEs and rate constants of the D-polymer doped with 8wt% DMAC-TRZ, DACT-II and TPA-DCPP films, respectively, and the neat TADF emitter films.

Emitter	D-polymer	Φ_F [%][a]	Φ_p [%][b]	Φ_d [%][c]	k_p [10^7 s][d]	k_d [10^5 s][e]	k_{IC} [10^6 s][f]	k_{ISC} [10^7 s][g]	k_{RISC} [10^5 s][h]
DMAC-TRZ	P(DMAC-Ge)	91	54.6	36.4	2.96	3.53	2.92	2.17	3.22
	P(DMAC-Si)	87	58.0	29.0	3.45	3.51	5.15	1.98	3.05
	P(DPA-Si)	71	31.9	22.1	1.59	2.11	13.22	2.03	1.14
	P(Cz-Si)	67	56.0	11.0	3.59	3.00	17.67	1.05	2.01
DACT-II	P(DMAC-Ge)	99	85.4	13.6	7.07	6.08	0.71	1.13	6.02
	P(DMAC-Si)	98	87.4	10.6	7.44	5.89	1.52	0.92	5.77
	P(DPA-Si)	90	75.0	15.0	5.07	4.99	5.64	1.13	4.49
	P(Cz-Si)	61	57.0	4.0	4.68	3.89	29.9	0.54	2.38
TPA-DCPP	P(DMAC-Ge)	23	22.5	0.5	0.40	1.86	13.24	0.04	0.43
	P(DMAC-Si)	21	20.5	0.5	0.35	1.55	13.00	0.04	0.33
	P(DPA-Si)	6	5.7	0.3	0.10	0.50	15.60	0.07	0.03
	P(Cz-Si)	61	58.2	2.8	2.56	27.90	16.40	0.20	17.00

[a] PLQY in film state. [b]PLQY for the prompt fluorescent (Φ_p) component calculated from ϕ_p=PLQE×(% of τ_p). [c]PLQY for the delayed fluorescent (Φ_d) component calculated from ϕ_d=PLQE×(% of τ_d). [d]The rate constant of prompt component (k_p). [e]The rate constant of delayed component (k_d). [f]The rate constant of internal conversion (k_{IC}). [g]The rate constant of intersystem crossing (k_{ISC}). [h]The rate constant of reverse intersystem crossing (k_{RISC}).

S2.4 Exclusion of energy level and dipole moment effects on the PLQY of TADF emitter

To clarify if the host with shallow HOMO level or large excited-state dipole moment would lead to poor PLQY of TADF emitter, we choose the D-polymer host doped with DACT-II films as an example. First, in terms of the host materials with shallow HOMO levels would lead to exciton quenching of TADF guest,[S10-11] the shallower HOMO levels (about -5.40 eV) for P(DMAC-Si) and P(DMAC-Ge) and the deeper HOMO level (-5.72 eV) for P(Cz-Si) as shown in Table S1 should have the negative and positive effects on the PLQYs of DACT-II, respectively. However, the former ones give extremely high PLQYs (98% and 99%), and the latter one shows inferior PLQY (61%), indicating that the variations in energy levels in these σ-π conjugated polymer hosts do not predominate the PLQY of TADF guest.

On the other hand, we obtain the excited-state dipole moment for each side-arm fragment on these D-polymer hosts via the calculations of time-dependent DFT (TD-DFT) as shown in Figure S19. Their excited-state dipole moments show the sequence Ph-DMAC (9.75 D) > Ph-DPA (8.92 D) > Ph-Cz (4.83 D). If the large excited-state dipole moment intensifies the host-guest dipole-dipole interaction, which would increase the exciton quenching according to Han's report.[S12] By contrast, the DMAC based D-polymer hosts give the highest PLQY than the others after the addition of 8wt% DACT-II, showing no connection to the dipole-dipole interaction. After ruling out the energy level and dipole moment factors, we are sure that the PLQY magnitude of TADF guest is affected only by the D-polymer host/TADF-guest interaction.

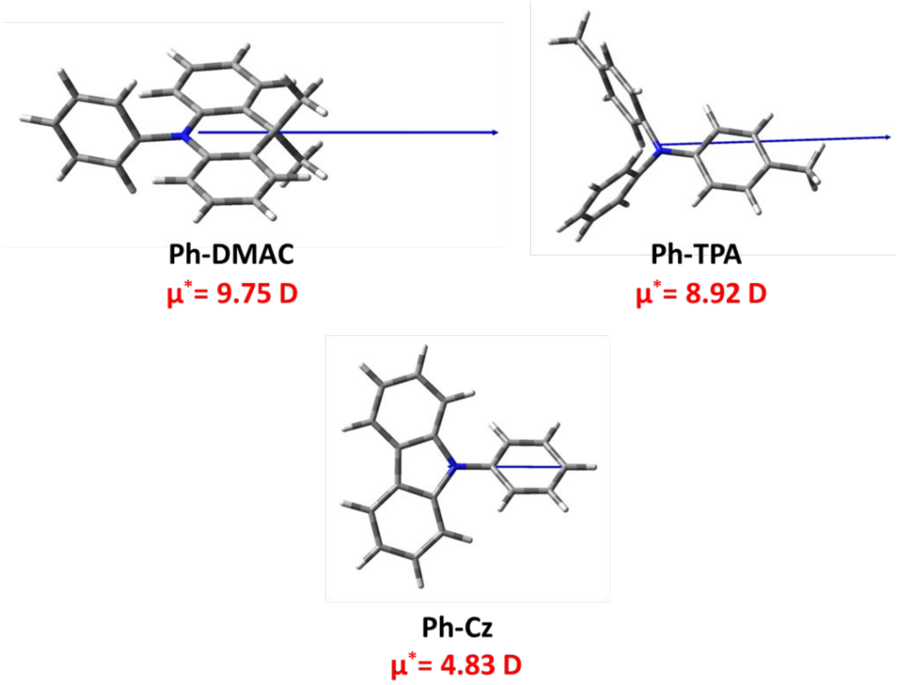

Figure S19. Excited-state structures and dipole moments for each side-arm fragment of these D-polymer hosts calculated by the TD-DFT using the Gaussian 09 package at the B3LYP/6-13G(d,p) level.

S3. Device Performances

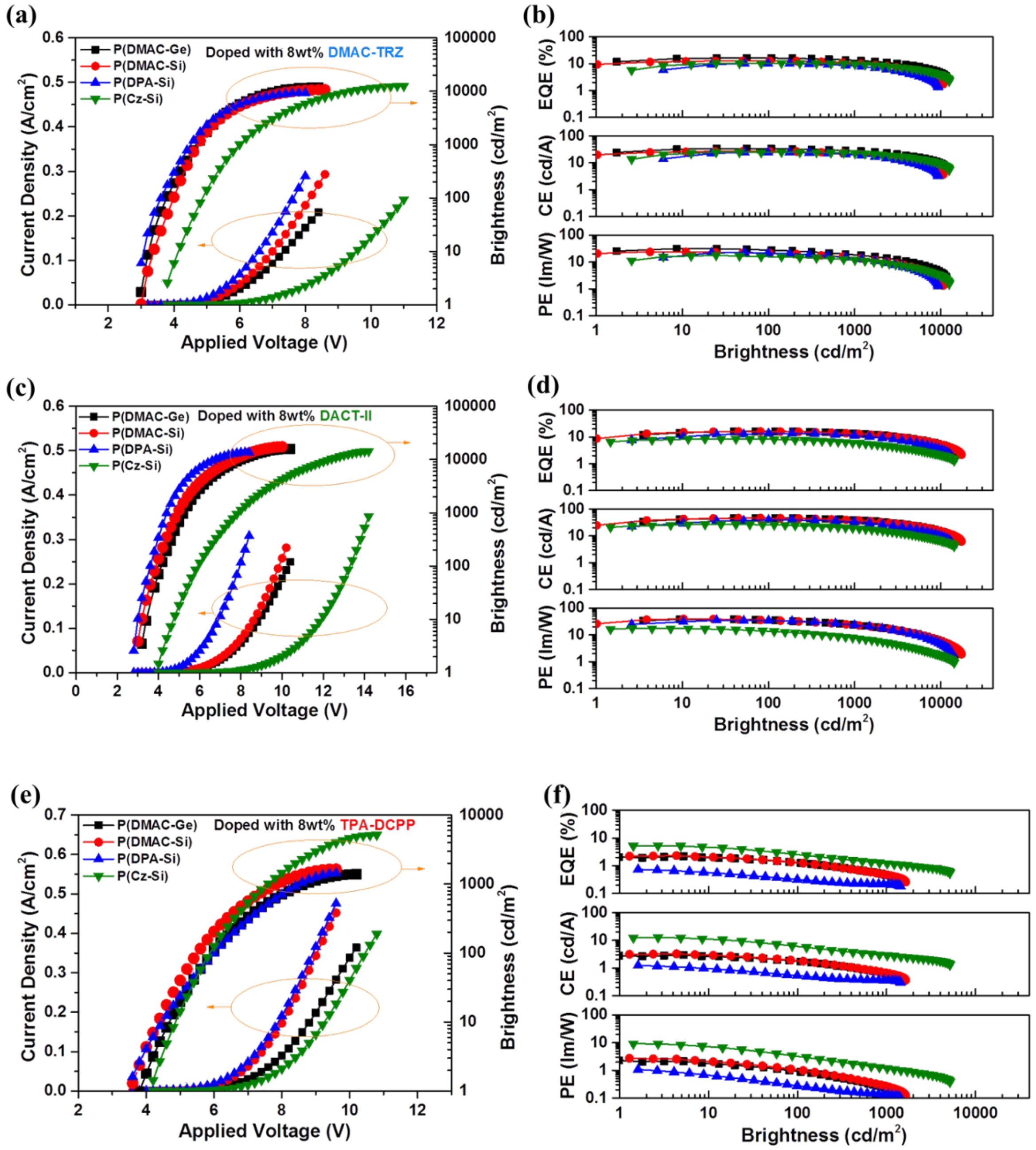

Figure S20. I-V-B characteristics and EQE, CE, PE versus brightness curves for a-b) sky-blue device, c-d) green device and e-f) red device using P(DMAC-Ge), P(DMAC-Si), P(DPA-Si) and P(Cz-Si) as host.

Table S8. Electroluminescence characteristics of the sB/G/R TADF PLEDs.

Emitter	Host	V_{on}[a] [V]	B_{max}[b] [cd/m^2]	LE_{max}[c] [cd/A]	PE_{max}[d] [lm/W]	EQE_{max}[e] [%]	EL_{max}[f] [nm]	CIE[g]
**DMAC-TRZ (Sky-blue)**	P(DMAC-Ge)	3.0	12,843	34.0	31.6	16.3	484	(0.15, 0.32)
	P(DMAC-Si)	3.0	10,666	26.8	24.0	12.7	484	(0.16, 0.32)
	P(DPA-Si)	3.0	9,288	24.5	22.8	10.2	489	(0.22, 0.38)
	P(Cz-Si)	3.8	12,469	24.8	17.7	10.4	494	(0.17, 0.40)
**DACT-II (Green)**	P(DMAC-Ge)	3.2	15,660	46.6	38.3	16.2	505	(0.22, 0.49)
	P(DMAC-Si)	3.0	17,328	45.9	39.7	16.0	505	(0.22, 0.49)
	P(DPA-Si)	2.8	13,623	39.6	35.3	13.6	505	(0.26, 0.49)
	P(Cz-Si)	4.0	14,244	26.7	17.6	8.4	515	(0.26, 0.55)
**TPA-DCPP (Red)**	P(DMAC-Ge)	3.8	1,388	3.0	2.3	2.2	636	(0.60, 0.39)
	P(DMAC-Si)	3.6	1,638	3.3	2.7	2.3	635	(0.59, 0.39)
	P(DPA-Si)	3.6	1,439	1.2	1.1	0.7	600	(0.56, 0.41)
	P(Cz-Si)	4.2	5,157	12.7	9.2	5.4	600	(0.55, 0.44)

[a]Turn-on voltage at 1 cd/m^2. [b]Maximum luminance. [c]Maximum luminance efficiency. [d] Maximum power efficiency. [e] Maximum external quantum efficiency (EQE). [f]PL emission peak [g]CIE coordinates at the operating voltage of 7V.

Side Chain Effects of D-polymer Hosts on EL Spectral Stability:

To explore the EL spectral stability for these σ-π conjugated polymer hosts on the application of sky-blue/red/green (sB/G/R) TADF devices, we use the Si-based D-polymers grafting with DPA, DMAC and Cz moieties on Si as hosts and the sky-blue, green and red TADF: DMAC-TRZ, DACT-II and TPA-DCPP as guest, respectively. The device structure is ITO/PEDOT:PSS (30nm)/ host: 8wt% TADF emitter (30nm)/ TP3PO (3nm)/ TmPyPB (52nm)/ CsF (1nm)/ Al (100nm). As shown in Figure S21, their EL emissions are clearly influenced by the side arm selection of D-polymer host. First, the P(DPA-Si) based devices have the most unstable EL emissions along with the increased driving voltage among the others in the sky-blue, green and red devices. For P(DPA-Si) based devices, besides the originated EL emissions with the peak values at 489, 505 and 600 nm from the sB/G/R TADF guests, all the P(DPA-Si) based sB/G/R devices contain the extra emission at ~580 nm. Moreover, their EL spectra are quite different from their PL spectra, where only the TADF guest emissions can be observed

(Figure 9). This emission at ~580 nm could result from a formation of triphenylamine (TPA) moiety electromer emission as it is identical to that observed in the previous report.[S13] This is further supported by the EL spectrum obtained from the device using P(DPA-Si) as EML without adding TADF dopant (Figure S22). It shows an extremely strong emission around 580 nm under electroexcitation, but this emission does not appear in the neat P(DPA-Si) film under photoexcitation (Figure S3), confirming that the emission at the long wavelenth region is the TPA (DPA plus phenyl on Si) electromer. Second, the P(DMAC-Si) based devices show the same EL spectra in sB/G/R emissions with the peak values at 484, 505 and 635 nm respectively under all the applied voltages, indicating that the twisted DMAC side arm is beneficial for supressing the formation of other emission species and thereby providing the stable EL spectra. Third, the P(Cz-Si) based devices show the stable EL spectra in sky-blue and green emissions, but have the unstable EL spectra in red emissions that produce the spectral blue-shift as increasing the drivring voltage from 5V to 7V, suggesting that this is possibly caused by the host/guest interaction. On the other hand, the red EL spectra of P(Cz-Si) based device have distinctly blue shift emissions than P(DMAC-Si) based device, where the emission peak is around 600 nm for the former and at 635 nm for the latter (Figure S21). Based on their PL analysis (Figure 9), we infer that strong $^1(D_h/A_g)^*$ emission also occurs in the red TADF doped P(Cz-Si) system that affects their EL emissions.

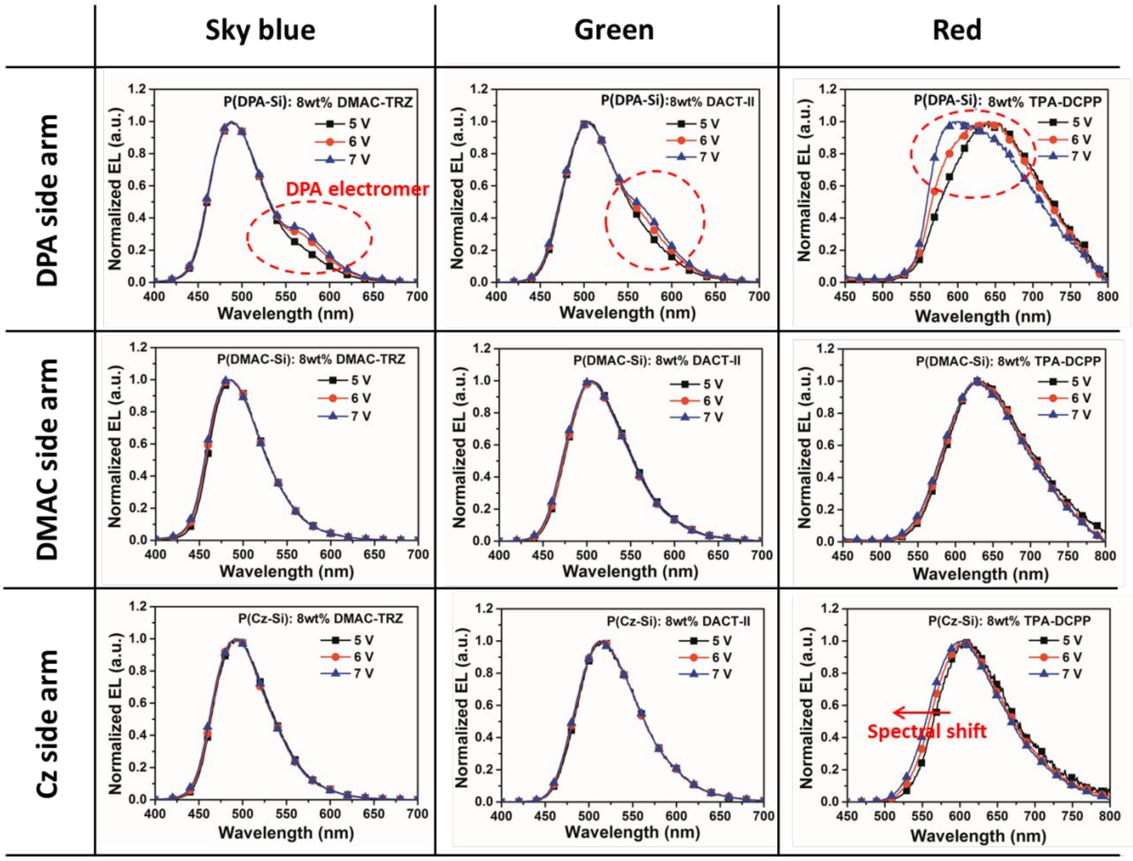

Figure S21. EL spectra of R/G/B devices for using P(DPA-Si), P(DMAC-Si) and P(Cz-Si) as host that graft with DPA, DMAC and Cz side arms, respectively.

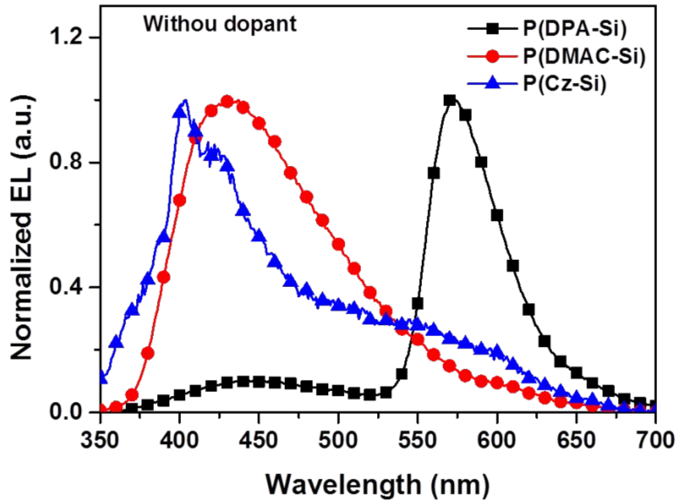

Figure S22. EL spectra for the devices using P(DPA-Si), P(DMAC-Si) and P(Cz-Si) as EML. The device structure: ITO/ PEDOT:PSS/EML (30nm) /TP3PO(3nm)/ TmPyPB (52nm)/ CsF (1nm)/Al.

S4. ¹HNMR spectra and ¹CNMR spectrum

¹H NMR of Br-DMAC-Si (500 MHz, $CDCl_3$)

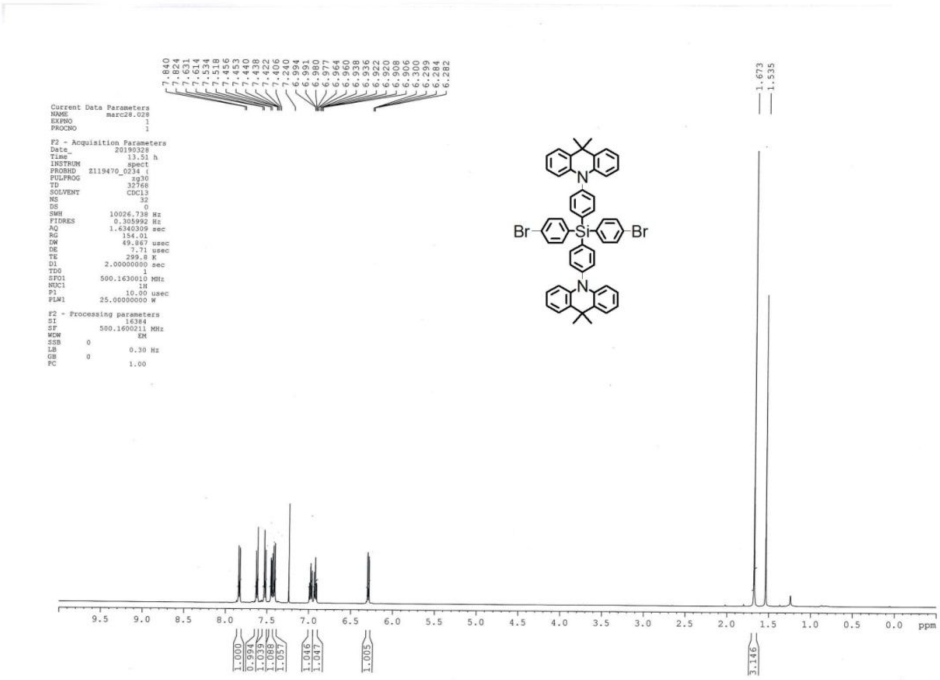

¹³C NMR of Br-DMAC-Si (125 MHz, $CDCl_3$)

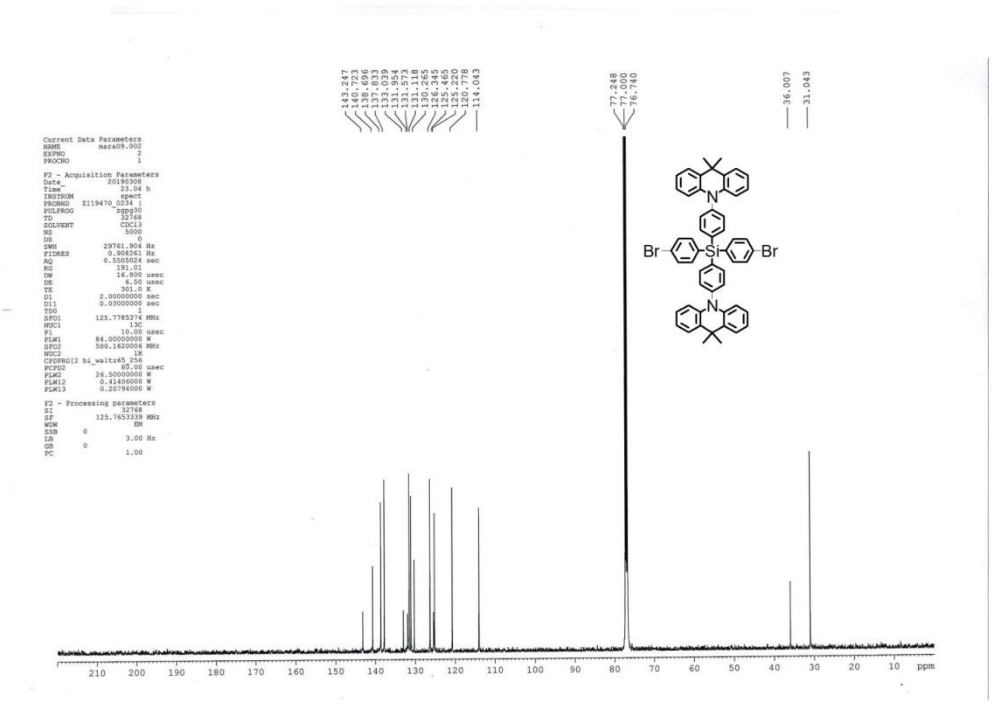

^{1}H NMR of Br-DPA-Si (500 MHz, $CDCl_3$)

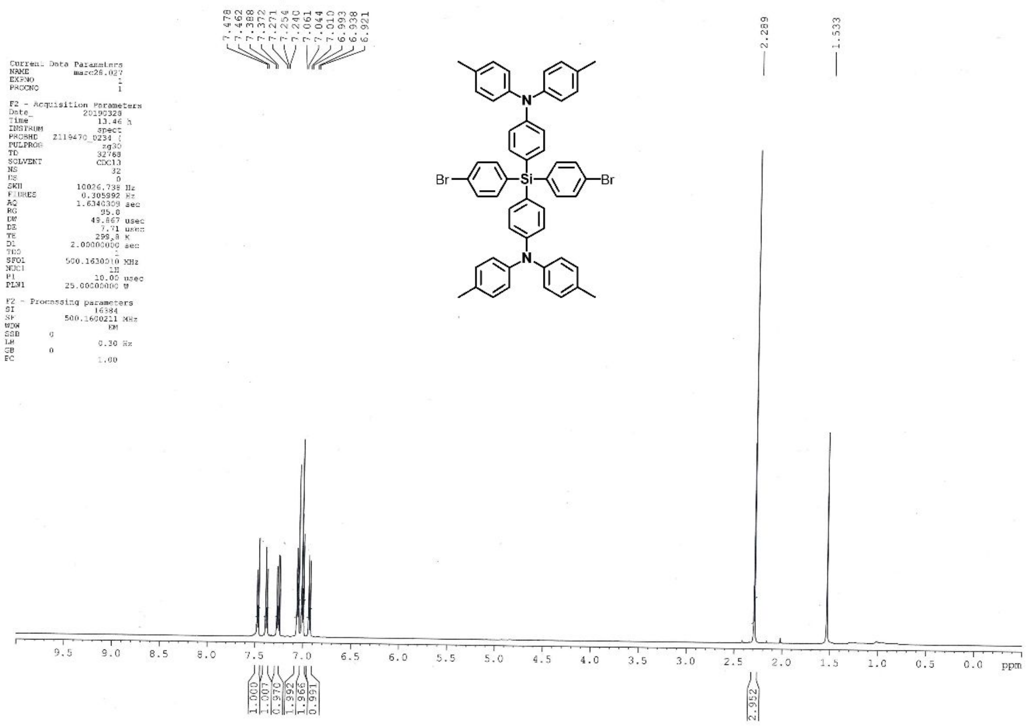

^{13}C NMR of Br-DPA-Si (125 MHz, $CDCl_3$)

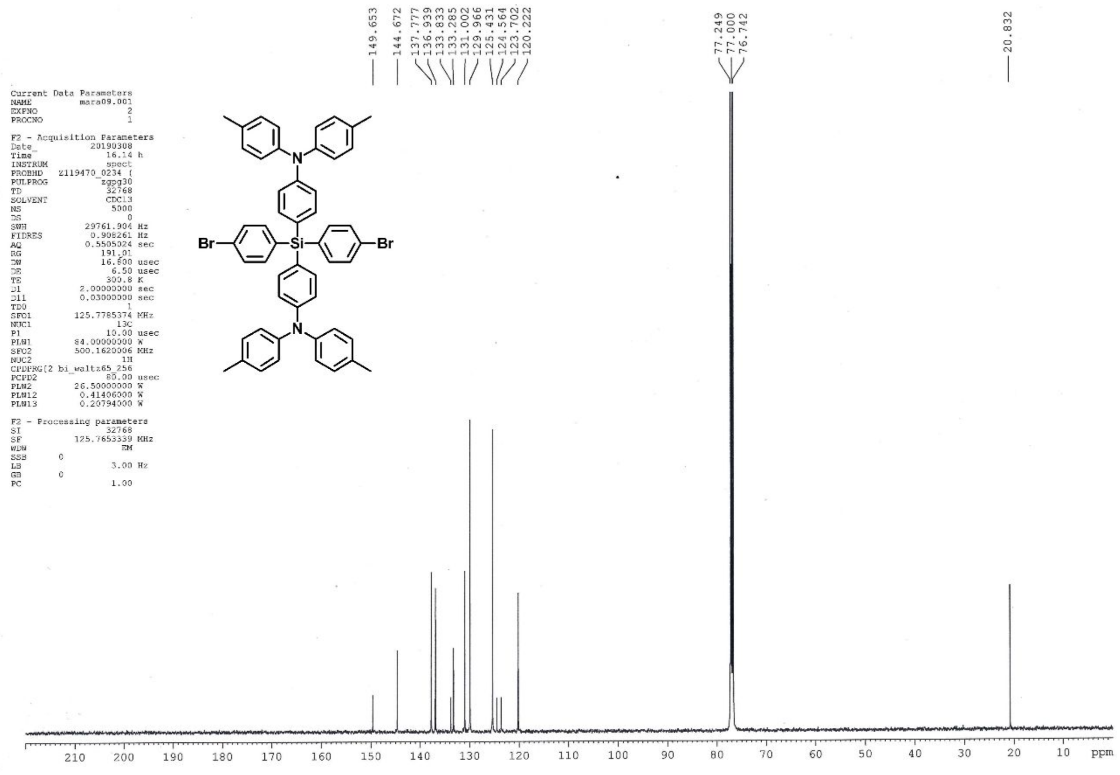

^{1}H NMR of Br-Cz-Si (500 MHz, $CDCl_3$)

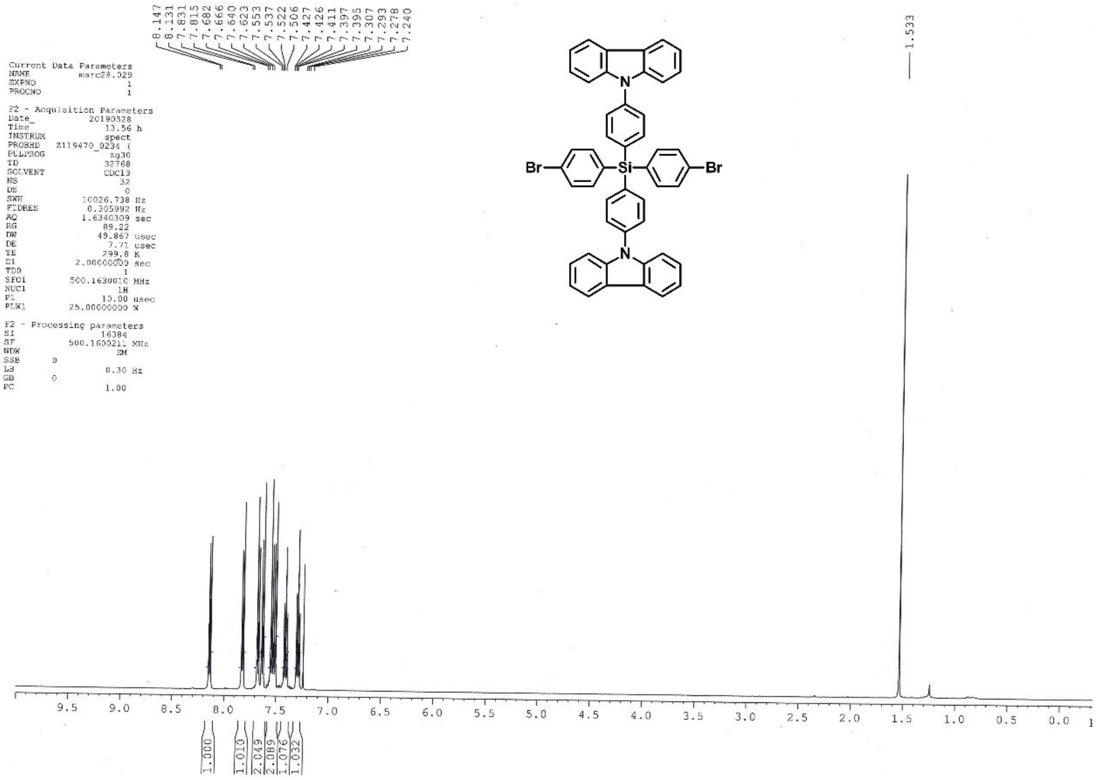

^{13}C NMR of Br-Cz-Si (125 MHz, $CDCl_3$)

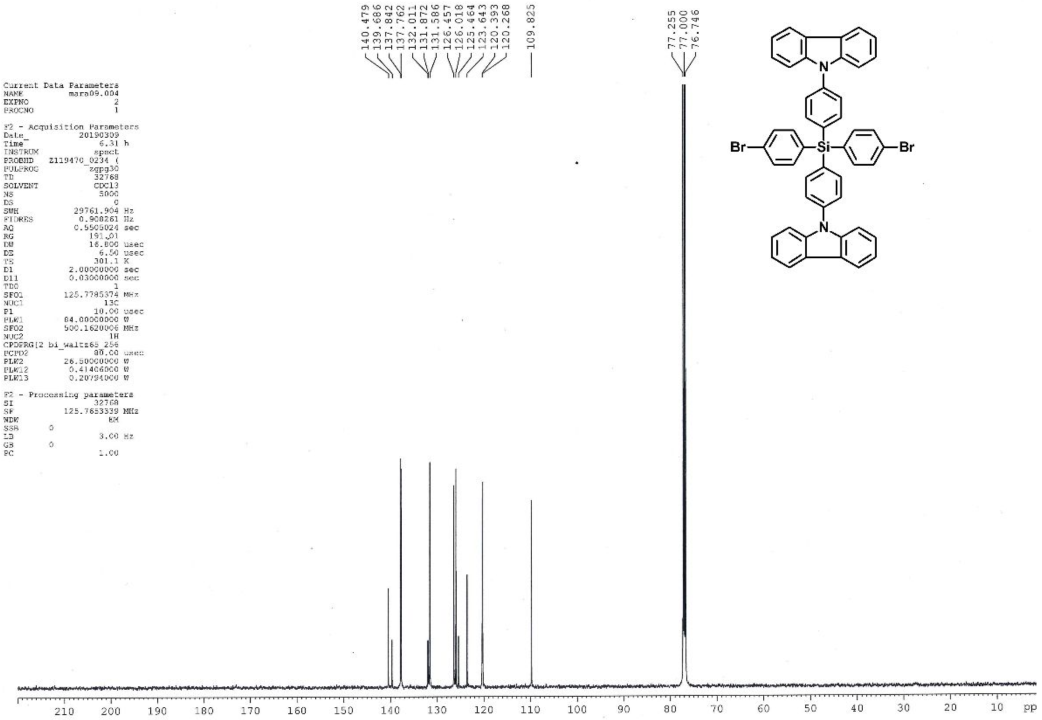

^{1}H NMR of P(DMAC-Si) (500 MHz, $CDCl_3$)

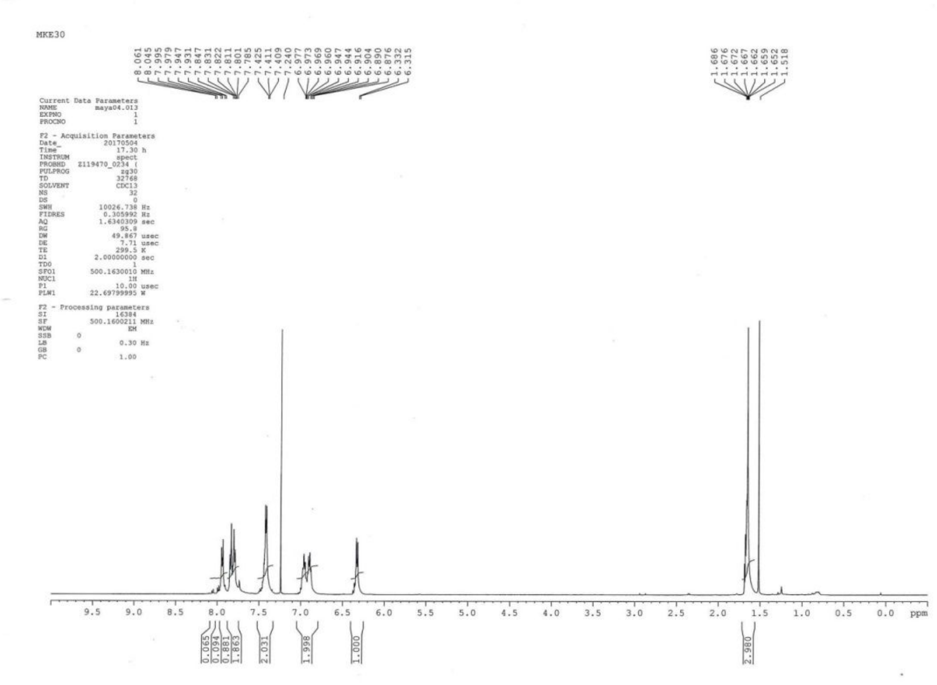

^{1}H NMR of P(Cz-Si) (500 MHz, $CDCl_3$)

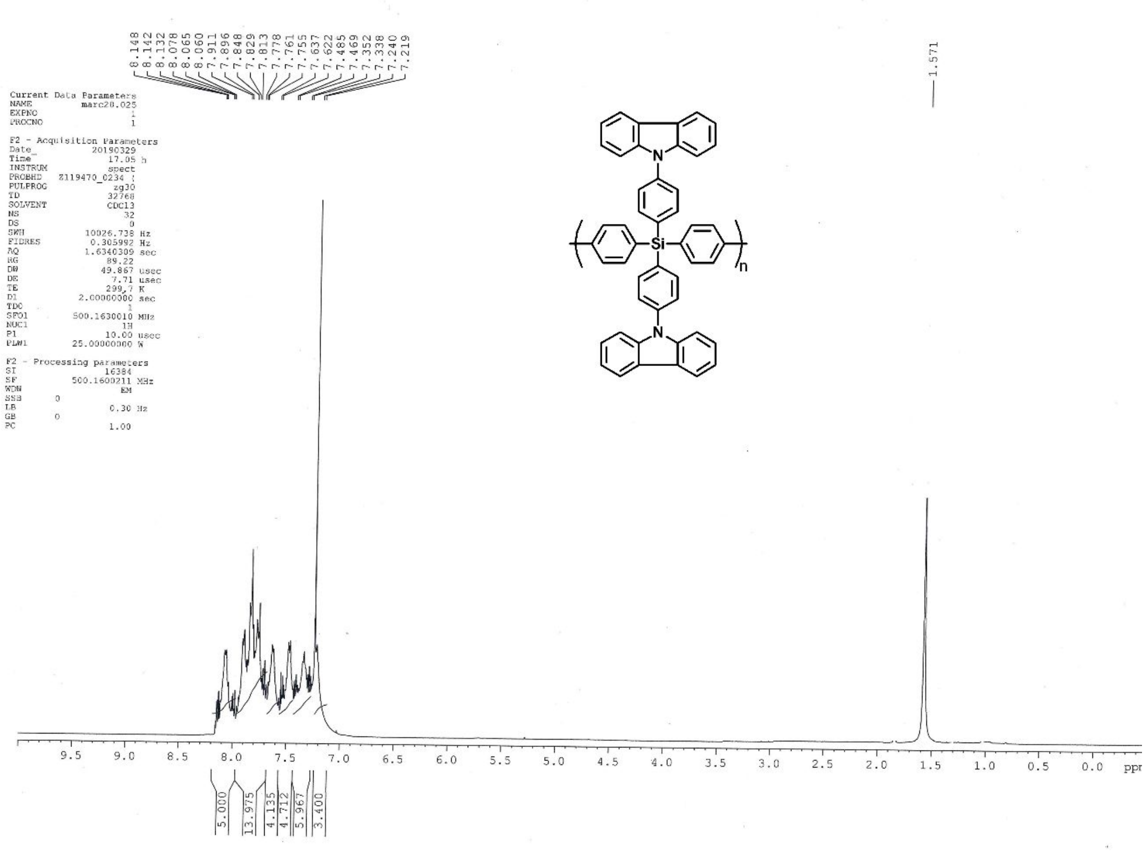

References

(S1) Zhang, B.; Wang, Z., Building Ultramicropores within Organic Polymers Based on a Thermosetting Cyanate Ester Resin. *Chem. Commun.* **2009**, 5027.
(S2) Chang, Y. T.; Sharma, S.; Hung, M. K.; Lee, Y. H.; Chen, S. A., Bipolar and Unipolar Silylene-Diphenylene sigma-pi Conjugated Polymer Route for Highly Efficient Electrophosphorescence. *Sci Rep* **2016,** *6*, 38404.
(S3) Sun, D.; Yang, Z.; Sun, X.; Li, H.; Ren, Z.; Liu, J.; Ma, D.; Yan, S., Synthesis of Triphenylamine Based Polysiloxane as a Blue Phosphorescent host. *Polym. Chem.* **2014,** *5*, 5046.
(S4) Sun, D.; Fu, Q.; Ren, Z.; Li, W.; Li, H.; Ma, D.; Yan, S., Carbazole-Based Polysiloxane Hosts for Highly Efficient Solution-Processed Blue Electrophosphorescent Devices. *J. Mater. Chem. C* **2013,** *1*, 5344.
(S5) Chang, Y. T.; Sharma, S.; Hung, M. K.; Lee, Y. H.; Chen, S. A., Bipolar and Unipolar Silylene-Diphenylene sigma-pi Conjugated Polymer Route for Highly Efficient Electrophosphorescence. *Sci. Rep.* **2016,** *6*, 38404.
(S6) Hung, M. K.; Tsai, K. W.; Sharma, S.; Wu, J. Y.; Chen, S. A., Acridan-Grafted Poly(biphenyl germanium) with High Triplet Energy, Low Polarizability, and an External Heavy-Atom Effect for Highly Efficient Sky-Blue TADF Electroluminescence. *Angew Chem. Int. Ed. Engl.* **2019,** *58*, 11317.
(S7) Tamai, Y.; Ohkita, H.; Benten, H.; Ito, S., Triplet Exciton Dynamics in Fluorene−Amine Copolymer Films. *Chem. Mater.* **2014,** *26*, 2733.
(S8) Wu, T.-L.; Huang, M.-J.; Lin, C.-C.; Huang, P.-Y.; Chou, T.-Y.; Chen-Cheng, R.-W.; Lin, H.-W.; Liu, R.-S.; Cheng, C.-H., Diboron Compound-Based Organic Light-Emitting Diodes with High Efficiency and Reduced Efficiency Roll-Off. *Nat. Photonics* **2018,** *12*, 235.
(S9) Zhang, Q.; Kuwabara, H.; Potscavage, W. J., Jr.; Huang, S.; Hatae, Y.; Shibata, T.; Adachi, C., Anthraquinone-based intramolecular charge-transfer compounds: computational molecular design, thermally activated delayed fluorescence, and highly efficient red electroluminescence. *J. Am. Chem. Soc.* **2014,** *136*, 18070.
(S10) Nakanotani, H.; Masui, K.; Nishide, J.; Shibata, T.; Adachi, C., Promising Operational Stability of High-Efficiency Organic Light-Emitting Diodes Based on Thermally Activated Delayed Fluorescence. *Sci. Rep.* **2013,** *3*, 2127.
(S11) Kim, B. S.; Lee, J. Y., Engineering of Mixed Host for High External Quantum Efficiency above 25% in Green Thermally Activated Delayed Fluorescence Device. *Adv. Funct. Mater.* **2014,** *24*, 3970.
(S12) Han, C.; Zhang, Z.; Ding, D.; Xu, H., Dipole-Dipole Interaction Management for Efficient Blue Thermally Activated Delayed Fluorescence Diodes. *Chem* **2018,** *4*, 2154.
(S13) Kalinowski, J.; Giro, G.; Cocchi, M.; Fattori, V.; Di Marco, P., Unusual Disparity in Electroluminescence and Photoluminescence Spectra of Vacuum-Evaporated Films of 1,1-Bis

((di-4-tolylamino) phenyl) Cyclohexane. *Appl. Phys. Lett.* **2000,** *76*, 2352.
